# Conserved MicroRNA Act Boldly During Sprout Development and Quality Formation in Pingyang Tezaocha (*Camellia sinensis*)

**DOI:** 10.3389/fgene.2019.00237

**Published:** 2019-03-28

**Authors:** Lei Zhao, Changsong Chen, Yu Wang, Jiazhi Shen, Zhaotang Ding

**Affiliations:** ^1^Qingdao Key Laboratory of Genetic Improvement and Breeding in Horticultural Plants, College of Horticulture, Qingdao Agricultural University, Qingdao, China; ^2^Department of Plant Science and Landscape Architecture, University of Maryland, College Park, MD, United States; ^3^Tea Research Institute, Fujian Academy of Agricultural Sciences, Fu'an, China; ^4^College of Horticulture, Nanjing Agricultural University, Nanjing, China

**Keywords:** conserved miRNA, sprouts development, quality formation, transcription factors, *Camellia sinensis* (L.) O. Kuntze

## Abstract

Tea tree [*Camellia sinensis* (L.) O. Kuntze] is an important leaf (sometimes tender stem)-using commercial plant with many medicinal uses. The development of newly sprouts would directly affect the yield and quality of tea product, especially significant for Pingyang Tezaocha (PYTZ) which takes up a large percent in the early spring tea market. MicroRNA (miRNA), particularly the conserved miRNAs, often position in the center of subtle and complex gene regulatory systems, precisely control the biological processes together with other factors in a spatio-temporal pattern. Here, quality-determined metabolites catechins, theanine and caffeine in PYTZ sprouts including buds (sBud), different development stages of leaves (sL1, sL2) and stems (sS1, sS2) were quantified. A total of 15 miRNA libraries of the same tissue with three repetitions for each were constructed to explore vital miRNAs during the biological processes of development and quality formation. We analyzed the whole miRNA profiles during the sprout development and defined conserved miRNA families in the tea plant. The differentially expressed miRNAs related to the expression profiles buds, leaves, and stems development stages were described. Twenty one miRNAs and eight miRNA-TF pairs that most likely to participate in regulating development, and at least two miRNA-TF-metabolite triplets that participate in both development and quality formation had been filtered. Our results indicated that conserved miRNA act boldly during important biological processes, they are (i) more likely to be linked with morphological function in primary metabolism during sprout development, and (ii) hold an important position in secondary metabolism during quality formation in tea plant, also (iii) coordinate with transcription factors in forming networks of complex multicellular organism regulation.

## Introduction

Originally produced in China, green tea nowadays is the star among the top list beverages, attribute to its good taste, health benefits, and mysterious process, which brings considerable economic benefit in planting and exporting countries such as China, India, Kenya, and Sri Lanka. Based on reports from the China Tea Marketing Association 2017 (http://www.stats.gov.cn/), approximately 10.3 million tons of fresh tea leaves were harvested to produce various tea products. As young leaves and tender stems from the tea tree are processed to prepare “tea”, the developmental characters are supposed to have a direct and significant bearing on the yield and the quality of tea product.

MicroRNAs (miRNAs), are endogenous single-stranded non-coding small RNAs, that could both regulate their target messenger RNAs (mRNAs) at chromatin state and could also perfectly or imperfectly bind to their targets for further translation suppression by cleaving at some complementary site (Rubio-Somoza and Weigel, 2011; Zheng et al., 2015). The plant miRNA families were thus placed to be at the central position within gene expression programs, always with small numbers per cell and large amounts of transcripts (Voinnet, 2009), yet have powerful effect in developmental regulation, morphogenesis, stress responses (Axtell and Bowman, 2008; De Lima et al., 2012; Jones-Rhoades, 2012; Yang et al., 2013). Still, seldom gene regulation could be completed without consideration of the transcription factors. Many transcription factors in the plant kingdoms are highly conserved even stride over large evolutionary distances, and for some of them, they could still share similar developmental roles in diverse species (Zhao et al., 2013; Xu et al., 2016). Hypothesis demonstrated that the miRNA binding sites evolve faster than the transcription factor binding sites, as the ways to repress a gene is relatively much more than to activate one (Chen and Rajewsky, 2007).

However, some miRNA seems extremely well conserved (Lu et al., 2006). A large portion of the conserved miRNAs and their conventional target transcription factors as well as F-box proteins play pivotal roles in governing plastic behavior during development, such as phase change and plant architecture (Kidner, 2010; Rubio-Somoza and Weigel, 2011), making miRNA-TF mRNA pairs more fascinating. At least 7 kinds of miRNAs were widely reported to regulate in the three stages of leaf development and leaf morphology. At the initiation stage, a division of leaf primordia are commonly considered to be the key stage in the leaf development process, which comes from a group of cells localized on the flanks of the shoot apical meristem (SAM) loses their indeterminacy (Micol and Hake, 2003). During this stage, miR390/ARF pathway has been described in the regulation of leaf polarity (Braybrook and Kuhlemeier, 2010). miR165/166 regulates the leaf polarity by targeting the HD-ZIP genes and thus control the adaxial cell fate (Rubio-Somoza and Weigel, 2011; Sun, 2012). Recent discovery revealed that the leaf dorsoventral polarity (adaxial-abaxial) signals which may cause mechanical heterogeneity of the cell wall, is linking to the methyl-esterification of cell-wall pectins in tomato and *Arabidopsis* (Qi et al., 2017). The shape and architecture of leaf need the orchestration of auxin, *KNOX* genes and miRNA regulation. *KNOX* genes could be down-regulated by CUC transcriptional regulators, which are important for organ boundaries building (Takada and Tasaka, 2002; Chen, 2009), floral patterning, and leaf morphogenesis (Micol and Hake, 2003; Engstrom et al., 2004). NAC (NAM, CUC1/2-like) is one branch of CUC gene family regulated by miR164. MiR164/GOB (a CUC2 ortholog gene), well-studied in tomato, is necessary for controlling leaf polarity and determining the serration or smooth of the leaf boundaries (Berger et al., 2009). MiR319, encoding by three loci including miR-JAW (miR319a) in *Arabidopsis*, regulates five *TEOSINTE BRANCHED/CYCLOIDEA/PCF* (TCP) family members (Palatnik et al., 2003, 2007), which could also lead to the regulation of CUC genes. Overexpression of miR319 or loss function of these five *TCP* genes would result in crinkly leaves (Palatnik et al., 2003; Liu et al., 2018). TCP regulated growth and senescence via jasmonic acid synthesis pathway (Schommer et al., 2008). The cell number and cell size, which reported to be precisely spatial and temporal controlled (Usami et al., 2009), are mainly regulated by an SQUAMOSA PROMOTER BINDING PROTEIN PROTEIN-LIKE (SPL)-dependent pathway (Ferreira e Silva et al., 2014; Xu et al., 2016). In *Arabidopsis*, miR156 targets 11 of the 17 SPL genes, among which SPL3, 4, and 5 accelerates the juvenile-to-adult phase change, SPL9 and SPL15 regulate plastochron length (Wang et al., 2008; Wu et al., 2009; Xu et al., 2016). MiR396 plays an important role in plant leaf growth and development, most likely by repressing Growth-Regulating Factor (GRF) genes in *Arabidopsis*. Transgenic miR396-overexpressing plants have narrow-leaf phenotypes due to a reduction in cell number (Liu et al., 2009).

During the long cultivation history for more than 2,000 years in China, numerous elite tea varieties have been bred for different characteristics like early germination, high yield, good performance under environmental stress, and distinctive aroma or flavor. *Camellia sinensis* (L.) O. Kuntze “Pingyang Tezaocha” (PYTZ), an elite cultivar with short internodes selected in Zhejiang Province in the late century, is now popularized in the north tea area in China attributes to its high yield for about 3tons green tea products per hectare [Data from e-China tea from Tea Research Institute of China Academy of Agriculture Sciences AS (TRI, CAAS)] (http://www.e-chinatea.cn/other_shujuku.aspx) (Zhao et al., 2017). What's more, its early germination in April helps taking up a large percent in the early spring tea market annually (Yang, 2015). In tea plant, phenolic compounds is one of the most important secondary metabolites, accounting for 18% to 36% dry weight in the fresh leaves and tender stem (Jiang et al., 2013), is also the main flavor components and functional ingredients that had been intensely studied in the past decades for its effective and extensive pharmacological activities (Zhao et al., 2013). Accordingly, the accumulation of some phenolic-like nongalloylated catechins like epigallocatechin (EGC) and epicatechin (EC) (Zhao et al., 2017), quinic acid and flavonol glycosides are gradually increasing along with the developing stages (Jiang et al., 2013). What's more, some key enzyme genes involved in the biosynthetic pathway of phenolic compounds in different organs and leaves at different developmental stages also have the similar expression patterns, such like CsDHQ/DHS2 (DHQ/DHS, 3-dehydroquinate synthase), CsCHS1 (CHS, chalcone synthase), CsUGT78E1 (UGT, uridine diphosphate Glycosyltransferase), Cs4CL1 (4CL,4-coumaroyl-CoA ligase), CsF3′H1 (F3′H, flavonoid 3′-hydroxylase), and some TF genes like Sg4 of CsMYB family (Jiang et al., 2013; Li et al., 2017a), CsMYB5-1 and bHLH24-3 (Jiang et al., 2013). Beyond catechins in tea plant, theanine and caffeine are the other two characteristic constituents determine tea quality (Xia et al., 2017). No matter it is primary or secondary, there is no doubt that metabolisms synchronize along with plant growth and development, are under the precise entire spatio-temporal network control (Chen and Rajewsky, 2007).

Here, the content of three kinds of main taste compounds catechins, theanine, and caffeine in different tissues of spring sprouts including bud, two stages of leaves, and stems of PYTZ were quantified by High-Performance Liquid Chromatography-Mass Spectrometry (HPLC-MS/MS). MiRNA libraries of the same tissues were constructed by Illumina HiSeq technology in order to explore how miRNA works between development and quality formation. Key miRNAs involved in regulating sprout development have been speculated based on computational expression. Conserved miRNA families in tea plant were obtained and mainly studied. To what extent the conserved miRNAs might be linked with morphogenesis function during sprout development was further investigated through other six morphologically-different tea cultivars. Regulations in metabolic pathways of conserved miRNA together with their target genes, especially transcription factor genes that would finally determine tea quality have been studied and discussed. The consistency of performance between development and quality need more cross understanding and balance in the subsequent process of screening tea cultivars.

## Materials and Methods

### Plant Material and RNA Isolation

**Figure 1:**
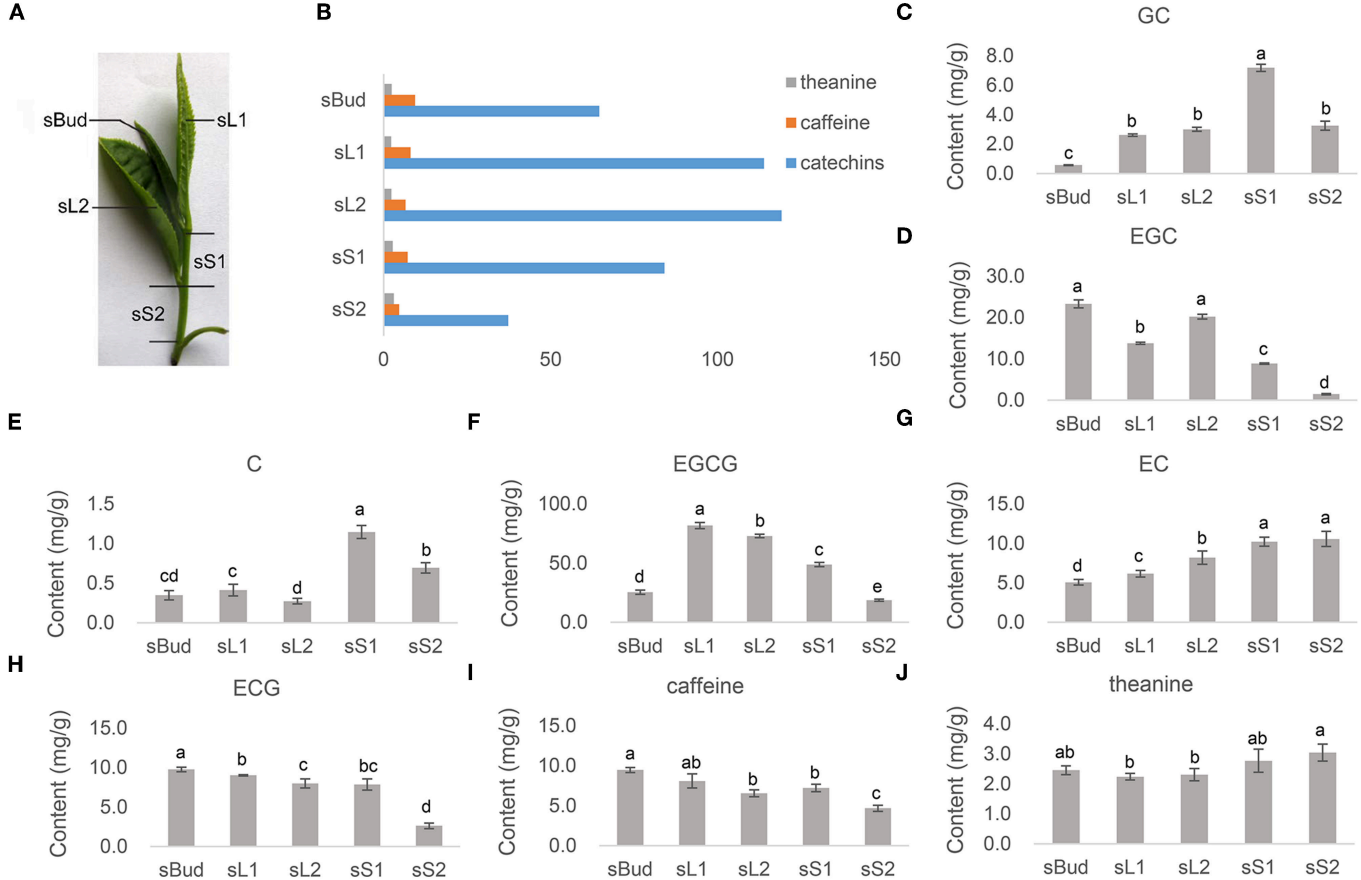
The contents of characteristic metabolites. **(A)** Samples of bud, leaves, and stems were collected orderly downwards from the top, named by sBud, sL1, sL2, sS1, and sS2, from buds, leaves, and stems, correspondingly. **(B)** The content of total catechins, caffeine, and theanine. **(C–J)** The content of GC, EGC, C, EGCG, EC, ECG, caffeine, and theanine in each tissue detected by HPLC-MS/MS. Standard catechins were (-)-gallocatechin (GC), (-)-epigallocatechin (EGC), (+)-catechin (C), (-)-epigallocatechin gallate (EGCG), (-)-epicatechin (EC), and (-)-epicatechin gallate (ECG). (Above each column, the different low case letters mean significant difference, *p* < 0.05, while labeled with the same ones mean insignificance, *p* > 0.05. *p*-values were calculated with Student's *t*-test).

The four-year-old tea plant cultivar *Camellia sinensis* (L.) O. Kuntze “Pingyang Tezaocha” (PYTZ) were planted in the Germplasm of Qingdao Tea Repository at the Tea Research Institute located in Qingdao (35°N119°E, Qingdao city, China) under natural light condition. To ensure the successiveness of gene expression during the development of newborn branch, we collected the samples of bud, leaves, and stems orderly downwards from the top (Figure 1A) (Shen et al., 2019). For normalization, the buds about 3 cm long (sBud), and the first leaf below the bud (sL1) about 3.5cm long, the second leaf with higher maturity below the bud (sL2) 4.5cm long, the stem between the first leaf and the second leaf (sS1) with about 1.5cm long, and the more mature stem between the second leaf and the fish leaf (sS2) with 2.1cm long were measured and collected. For collecting samples of RNA, healthy buds, leaves and stems at different developmental stages were collected and frozen immediately in liquid nitrogen and stored in −80°C freezers before use (Fan et al., 2015). Three biological replicates were collected and pooled from at least five individuals, and each biological replicate contained more than five buds, leaves and stems. The total RNA for each sample was extracted using TRIzol reagent (Invitrogen, Burlington, ON, Canada). The quality, purity, concentration, and integrity of the total RNA was checked using 1% agarose gel electrophoresis, NanoDrop Photometer Spectrophotometer (IMPLEN, Westlake Village, CA, USA), Qubit RNA Assay Kit in Qubit 2.0 Fluorometer (Life Technologies, Carlsbad, CA, USA), and RNA Nano 6000 Assay Kit of the Bioanalyser 2100 system (Agilent Technologies, Santa Clara, CA, USA), respectively. RNA samples with a 260/280 ratio between 1.8 and 2.0, 260/230 ratio between 2.0 and 2.5, and RNA integrity number more than 8.0, were used for sequencing and quantitative PCR analysis described below.

### Extraction and Quantification of Catechins, Caffeine, and Theanine

The extraction of catechins and caffeine followed by a previously described method with minor modifications (Jiang et al., 2013; Wang et al., 2018): 0.2 g of each fresh samples (sBud, sL1, sL2, sS1, and sS2) were ground in liquid nitrogen and extracted with an extraction solution (80% methanol and 20% water), followed by vortexing and sonicating for 30 min at a low temperature. Then, the samples were centrifuged at 3,500 g for 15 min, and the residues were re-extracted twice as mentioned above until the final volume of the pooled supernatants was 2 mL. The supernatants were then extracted three times with chloroform and three times with ethyl acetate. The pooled supernatant was concentrated to remove the ethyl acetate at a low temperature with a vacuum pump. Finally, the product was dissolved in 200 μL methanol for quantification. The theanine was extracted as reported by the method of Jeon et al. (2017) with some modifications. One gram of each finely ground sample was mixed with 100 mL boiling distilled water and brewing for 10 min (with the help of magnetic stirrer). All the obtained extract were filtered by 0.45 μ nylon membrane (after cooling down) and approximately 1 ml of the sample solution were centrifuged at 13,000 rpm for 10 min prior to HPLC analysis.

The isolation and detection of quality-related metabolites catechins, caffeine, and theanine in the sprouts of PYTZ were performed by high performance liquid chromatography-mass spectrometry (HPLC-MS/MS). HPLC analyses were performed on an Agilent 1298 LC system (Agilent, Santa Clara, CA, USA), and MS/MS detection was carried out using an Agilent 6460 Series Triple Quadrupole instrument (Agilent). Caffeine, theanine and six major tea standards: catechins, (-)-epigallocatechin gallate (EGCG), (-)-epigallocatechin (EGC), (-)-epicatechin gallate (ECG), (-)-epicatechin (EC), (-)-gallocatechin (GC), and (+)-catechin (C) were purchased from Sigma (St Louis, MO, USA). An Agilent 20RBAX RRHD Eclipse Plus C18 column (particle size: 1.8 mm, length: 100 mm, and internal diameter: 2.1 mm) was used at a flow rate of 1 mL min^−1^. For catechins and caffeine, the mobile phase consisted of 0.4% acetic acid in water and 100% acetonitrile; and the gradient of latter increased linearly from 0 to 10% (v/v) within 5 min, and to 35% at 20 min, to 10% at 21 min, to 1% at 25 min. For theanine, the mobile phase consisted of HPLC water and acetonitrile; and the gradient of former remained at 100% within 10 min, and decreased linearly from 100% to 20% (v/v) to 12 min, and kept at 20% to 20 min, to 100% at 22 min, and kept at 100% to 40 min. Mass spectra were acquired simultaneously using electrospray ionization in the positive and negative ionization modes over the range of m/z 100 to 2000. A drying gas flow of 6 L min^−1^, drying gas temperature of 350°C, nebulizer pressure of 45 psi, and capillary voltages of 3,500 V were used. The compounds were identified qualitatively using LC-MS by comparing the retention times (t_R_), wavelengths of maximum absorbance (λ_max_), protonated/deprotonated molecules ([M+H]^+^/[M–H]^−^), and major fragment ions with those of the authentic standards and published literature (Jiang et al., 2013; Jeon et al., 2017; Wang et al., 2018).

### Library Construction and Small RNA Sequencing

For sRNA library construction, 3 μg of total RNA per sample was used for the RNA sample preparations. Sequencing libraries were generated using NEBNext Multiplex Small RNA Library Prep Set for Illumina (NEB, USA). The library preparations were sequenced on an Illumina HiSeq™ 2500 sequencer, by Gene Denovo Biotechnology Co. (Guangzhou, China). The generated 50 bp single-end reads were then filtered out the impure sequences (adaptor sequences and the low quality reads) and removed cellular structural RNAs such as rRNA, snoRNA, snRNA, and tRNA based on the alignment with small RNAs in GeneBank database (Release 209.0) and Rfam database (11.0). The clean reads were mapped to the tea tree genome without mismatch to analyze their expression and distribution (NCBI Sequence Read Archive Database under accession PRJNA381277). Tags that mapped to exons or introns and repeat sequences were also removed.

### Identification of Known miRNAs and Novel miRNA

Since tea miRNA dataset was not included in the miRBase, the clean tags were subjected to a Blastn search against miRBase 21.0, to identify and annotate known miRNAs from all other plant miRNAs, allowing two mismatches. All the known miRNAs were further checked for the existence through 72 plant species, to figure out their conservative property. The unannotated tags were aligned with tea tree genome to identify novel miRNA candidates according to their genome positions and hairpin structures predicted by software Mireap (https://github.com/liqb/mireap, version 0.20).

### miRNA Expression Profiles and Prediction of Target mRNAs

The expression levels of both known miRNA and novel miRNA from each sample were calculated and normalized to transcripts per million (TPM) (Wu et al., 2017b). The formula is TPM = Actual miRNA counts / Total counts of clean tags^*^10^6^. Meanwhile, the correlation coefficient between every two replicas was calculated to evaluate repeatability between samples. Differential expression analysis across samples was performed using the DEGseq (2010) R package. miRNAs with *p* < 0.05 and log2-fold change ≥ 2 in comparison were set as the threshold for significantly differentially expressed miRNAs (DEM). Candidate target genes were predicted by using software PatMatch (Version 1.2) blasting against tea tree genome, abiding by some rigorous parameters as follows: No more than four mismatches between sRNA/target (G-U bases count as 0.5 mismatches); For the miRNA/target duplex (5′ of miRNA), (a) no more than two adjacent mismatches, (b) no adjacent mismatches in positions 2–12, (c) no mismatches in positions 10–11, (d) no more than 2.5 mismatches in positions 1–12, and the minimum free energy (MFE) of the miRNA/target duplex should be no < 60% compared to the MFE of the miRNA bound to its perfect complement (Yan et al., 2005; Wu et al., 2017a).

### Functional Enrichment Analysis of Target mRNAs

Gene Ontology (GO) enrichment analysis and KEGG pathway analysis were performed to the target mRNAs of DEM in order to comprehensively figure out their biological functions. All DEM target genes were mapped to GO terms in the Gene Ontology database (http://www.geneontology.org/), then the enriched significant GO terms (taking FDR ≤ 0.05 as a threshold, derived from calculated *p*-value) comparing to tea tree genome background were categorized into three levels, “biological process,” “cellular component” and “molecular function”. KEGG is the major public pathway-related database (Kanehisa et al., 2008) for further understand how genes interact with each other to play roles in certain biological functions. The calculating formula is the same as that in GO analysis. KEGG pathway enrichment analysis identify significantly enriched metabolic pathways or signal transduction pathways (Liu et al., 2014). Some online platforms or commercial services that based on same or different mathematical algorithms could help us to reconstruct gene networks, for example STRING (https://string-db.org/), Pathway Commons (https://www.pathwaycommons.org/) (Luna et al., 2016), ANDSystem (Ivanisenko et al., 2019), and so on (Saik et al., 2018). Functional enrichment of both target genes of miRNAs in single samples and DEM in a compare group were carried out in our analysis. Here, STRING was used to show the enrichment networks.

Trend analysis was aiming at the expression of all miRNAs performing in continuous tissues samples (Bud/L1/L2 and Bud/S1/S2) to cluster genes with similar expression patterns. Trend analysis was carried out by software Short Time-series Expression Miner (Ernst and Bar-Joseph, 2006) (under parameters -pro 20 -ratio 1.0). GO and KEGG pathway enrichment analysis was then be done to target genes of miRNAs in each trend, and the *p*-value was obtained by hypothesis testing. Those GO term and KEGG pathway were defined as significant ones satisfying Q value ≤ 0.05. Q value was that *p*-value corrected by FDR (Benjamin and Hochberg, 1995).

### Quantitative PCR for miRNAs and mRNAs

The expression profiles of mature miRNAs and the potential target mRNAs were further validated by quantitative PCR. Synthesis of the first strand cDNA was performed with Mir-X™ miRNA First-Strand Synthesis Kit (Cat. No. 638313, Clontech Laboratories, Inc., CA, USA), with 5.8S rRNA served as an internal control. The first strand cDNA of mRNA were synthesized by using PrimeScript™ RT reagent Kit with gDNA Eraser (Perfect Real Time) (Code No. RR047A, Takara, Tokyo, Japan), with *glyceraldehyde-3-phosphate dehydrogenase* (*GAPDH*) gene for normalization. The primers of miRNAs and mRNA were listed in Supplementary Table 1. Quantitative PCR was carried out with SYBR *Premix Ex Taq*™ IIKit (Tli RNase H Plus) (Code No. RR820A, Takara, Tokyo, Japan), on a LightCycler 480 instrument (Roche Molecular Systems, Inc., Indianapolis, IN, USA). The amplification program of miRNA was performed under the following parameters: 95°C for 10 min, 40 cycles at 95°C for 15 s, 60°C for 1 min (Zheng et al., 2015). The amplification program of mRNA was performed at 94°C for 10 s, 58°C for 10 s and 72°C for 10 s (Li et al., 2017b). Triplicates of each reaction were performed, and 5.8S rRNA and *GAPDH* were used as endogenous control separately. CT values obtained through quantitative PCR were analyzed using 2^−ΔΔ*CT*^ methods to calculate relative fold change values.

In addition, fresh spring sprouts were plucked from seven tea varieties from one experimental tea garden of Tea Research Institute, Fujian Academy of Agricultural Sciences in Fu'an, China (27°10′N, 119°35′E): *Camellia sinensis* “Jinfenghuang” (JFH), *Camellia sinensis* “Pingyang Tezaocha” (PYTZ), *Camellia sinensis* “Zhengdayin” (ZDY), *Camellia sinensis* “Dayewulong” (DYWL), *Camellia sinensis* “Huangdan” (HD), *Camellia sinensis* “Jiukeng 6” (JK), *Camellia sinensis* “Queshe” (QS). The bud, leaves, and stems were also sampled at the same position mentioned above for quantitative PCR analysis. The result of relative expressions was presented as a heatmap by using TBtools (Chen et al., 2018).

### Transcription Factor Prediction and miRNA-mRNA-Metabolite Network Construction

All the mRNA genes target by DEM predicted above were blasted against the transcription factor (TF) database from the plant (http://planttfdb.cbi.pku.edu.cn/, version 4.0) to annotate potential TFs. The resulting target TF genes were classified in each tissue for analysis. The resulting TF genes were further blasted against the tea tree genome (Xia et al., 2017) (NCBI Sequence Read Archive Database No. PRJNA381277). The methods for TF blasting and expression analysis were followed by Zhao et al. (2017). Different expression profiles were finally grouped into profile 1, 2, and so on. For the network analysis was based on Savoi's method (Savoi et al., 2016), the mRNA and metabolite association were obtained based on Pearson correlation coefficient between the contents of each metabolite and the expression levels of mRNA, and filtered the pairs when the absolute value of cor was larger than 0.9 and *p*-value was smaller than 0.05. The miRNA and mRNA association were obtained by Spearman's correlation coefficient according to their expression levels calculated by TPM and filtered the pairs when cor-value was no larger than −0.5, and the *p*-value was smaller than 0.05. The network was visualized by Cytoscape (V3.6.0) (Praneenararat et al., 2012).

### Availability of Supporting Data

Clean Illumina sequencing reads of 15 small RNA of PYTZ sprout have been deposited in the NCBI Sequence Read Archive Database under accession PRJNA510482.

## Results

### The Contents of Quality-Related Metabolites in Tea Sprouts

The buds, the first leaves, the first stems, sometimes with the second leaves together, are the most common raw materials for producing green tea. The contents of the most quality-related metabolites in tea sprouts that contributing flavors and health-promoting functions were quantified by HPLC-MS/MS (Figures 1B–J). Among the three kinds of characteristic metabolites, the content of total catechin accounts for the most majority proportion, while distributing significantly different among tender leaves and stems (Figure 1B). The galloylated catechins such like EGCG (Figure 1F) and ECG (Figure 1H), take up 67.4% in total catechin concentration obtained by summation of the individual components ranged from 37.3 to 119.2 mg/g (Figure 1B). Both EGCG and ECG had a relatively low concentration in sS2. The level of GC (Figure 1C), C (Figure 1E), and EC (Figure 1G), were found to be low in bud and leaves, especially for C. The concentrations of caffeine ranging from 4.7 to 9.7 mg/g, accumulated to its highest levels in sBud and lowest in sS2 (Figure 1I). Theanine, however, showed its highest accumulation in stems in PYTZ sprouts (Figure 1J).

### Overview of microRNA Profile and Its Mapping to Tea Tree Genome

**Figure 2:**
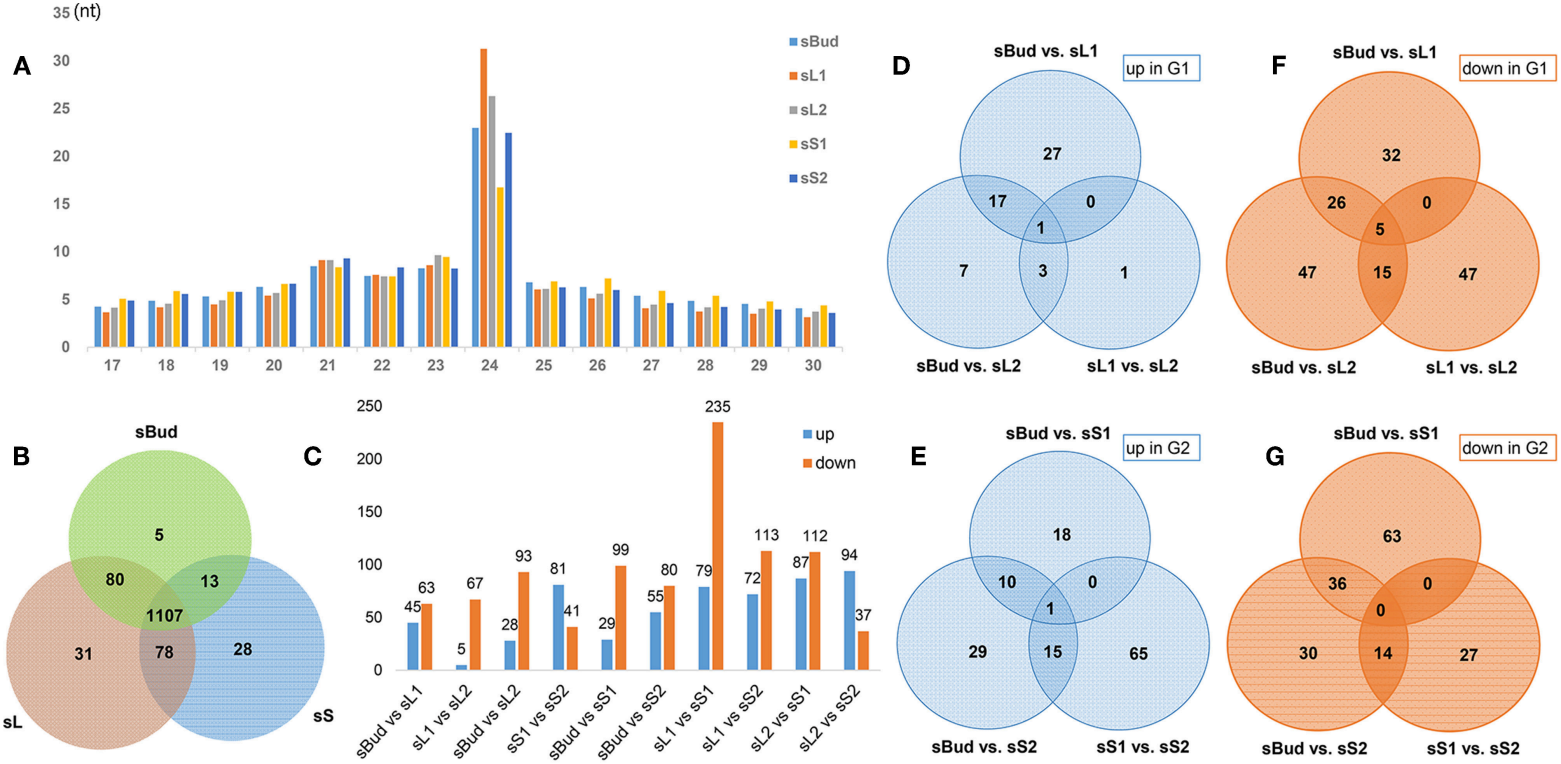
Overview of PYTZ microRNA libraries. **(A)** Size-class distribution of sRNAs in the sBud, sL1, sL2, sS1, and sS2 libraries. Designations the same as on previous figure. **(B)** Number statics of tissue-specific miRNAs. **(C)** Number statics of differentially expressed miRNAs (DEM) between each two sample groups. **(D–G)** The number of up/down-regulated DEM between different tissues in G1/G2.

To figure out what role the miRNA play during the formation of characteristic metabolites along with the development of the PYTZ sprout, 15 sRNA-Seq libraries including buds, leaves, and stems were separately sequenced on Illumina HiSeq™ 2500 platform generating a total of 292,653,360 raw reads. After removing dirty reads containing adapters and low quality bases, in average, the clean tags of 14,080,519 for sBud (bud), 12,776,739 for sL1 (the 1st leaf), 11,830,623 for sL2 (the 2nd leaf), 11,484,341 for sS1 (the younger stem), and 9,822,915 for sS2 (the older stem) were retained. The filtering data of each procedure were listed in Supplementary Table 2. Most clean tags had the length of 21-24nt, in which the 24 nt sRNAs were the most abundant (Figure 2A). The proportion of different length tags has no obvious difference among the five sample groups, and generally showed the trends of increased and then decreased bounded by the 24 nt sRNAs. Notably, the number of 24 nt sRNAs in sS1 was the lowest, while in sL1 was the highest.

About 76.97% clean tags were perfectly mapped to tea tree genome (NCBI Sequence Read Archive Database No. PRJNA381277), which indicated a credible quality of sequencing, and the rates of a genomic match were similar across these samples. We removed the tags mapped to exons located in positive-sense strands which might be fragments from mRNA degradation. (For statistics of mapping to tea tree genome, see Supplementary Table 3). The tags mapped to repeat sequences were also excluded. The clean tags were then aligned with the Rfam database (11.0) and the percentage of annotation was summarized in Supplementary Table 4. The average of rRNA, snRNA, snoRNA, and tRNA in the samples took up of 18.11, 0.19, 0.56, and 1.01%.

### Known miRNAs Identification and Novel microRNAs Prediction in PYTZ

To identify known miRNAs in tea, all the unannotated unique tags were blast-searched against plant miRNAs in miRBase (Release 21.0, June 2014). Overall, a total of 1,928,678 miRNA clean tags were identified from 15 libraries (Table 1), and 156 known miRNAs were identified (Supplementary Table 5). Most of the identified known miRNAs (81.97%) belonged to the 21 nt length miRNA families, the remaining ones belonged to 18–24 nt miRNAs families (Supplementary Table 5). For the 156 known miRNAs, 122 precursors and 99 kinds of characteristic hairpin structures of the known miRNAs were identified. The length of the precursors varied from 71 to 288 nt, with an average of 144 nt, and the average minimum free energy (MFE) was −57.23 kcal/mol, ranging from −22.3 to −90.2 kcal/mol (Supplementary Table 6). The abundance of miRNA including the novel miRNA showed a high distribution in leaf than in stem (ratio value in Table 1), with that in S1 the lowest.

**Table 1:** Numbers and ration of clean tags for conserved miRNA and novel miRNA.

**Sample**	**Total**	**Known miRNA**			**Novel miRNA**		
		**Mirna num**	**Tags uniq**	**Tags total/ratio**	**Mirna num**	**Tags uniq**	**Tags total/ratio**
sBud-1	13025496	90	2281	145547(1.12%)	986	1205	35371(0.27%)
sBud-2	13672142	87	2150	96863(0.71%)	780	1003	22599(0.17%)
sBud-3	15543919	86	2172	79234(0.51%)	669	876	23593(0.15%)
sL1-1	13217393	100	2571	237953(1.80%)	1000	1244	47097(0.36%)
sL1-2	12086276	98	3140	205017(1.70%)	1008	1276	36970(0.31%)
sL1-3	13026547	91	1967	127242(0.98%)	854	1066	30010(0.23%)
sL2-1	11146588	94	2523	151824(1.36%)	820	1037	26207(0.24%)
sL2-2	13556636	90	2518	162349(1.20%)	737	872	21904(0.16%)
sL2-3	10788646	90	2485	201288(1.87%)	783	1005	27762(0.26%)
sS1-1	10821241	73	1763	48215(0.45%)	508	693	11804(0.11%)
sS1-2	11025165	76	1753	48019(0.44%)	483	669	10145(0.09%)
sS1-3	12606617	81	1902	74064(0.59%)	659	852	18301(0.15%)
sS2-1	9125465	81	2079	114250(1.25%)	745	982	25010(0.27%)
sS2-2	11303334	90	2451	130149(1.15%)	847	1025	26705(0.24%)
sS2-3	9039947	79	2144	106664(1.18%)	781	945	21290(0.24%)
total	179985412	1306	33899	1928678(16.31%)	11660	14750	384768(3.25%)

For the four nucleic acids, the frequency of cytosine (C) (32.09%) and uracil (U) (29.65%) is higher than guanine (G) (19.26%) and adenosine (A) (19.00%). In the five samples, U had a high appearance at the 1st, 17th, 22th, and 23rd positions, with an average of 84.34, 61.80, 54.57, and 52.48%, respectively (Supplementary Figure 1). C occupied a very high percentage (87.44%) at 19th position. The analysis showed that A had a relatively high proportion at 9th position in the stem (sS1 and sS2) and 17th position in leaf (sL1 and sL2), in contrast, A was seldom present at 2nd, 13th, 18th, and 20th positions in the five tissues. For the first nucleotide bias analysis, U had the absolute predominance in miRNAs with the length of 20, 21, and 22 nt (Supplementary Figure 2).

The remaining reads which couldn't get mapped to known miRNAs were used to identify novel miRNAs. 384,768 novel miRNA tags were identified from 15 libraries (Table 1), and 1186 novel miRNA tags were identified by predicting the hairpin structures of their precursor sequences (Supplementary Table 7). The length of the novel miRNAs ranged from 18 to 27 nt, different with known miRNAs, the 22 nt length miRNA families were the most abundant (46.71%), followed by 21nt (41.23%). These novel miRNA were involved in 1130 hairpin miRNA precursors. The length of these precursors varied from 65 to 373 nt, with an average of 178 nt. The average minimum free energy (MFE) was −56.26 kcal/mol, ranging from −18.1 to −292.3 kcal/mol (Supplementary Table 7). The numbers of novel miRNAs were most in sL1 and lowest in sS1, the trend was the same with known miRNAs.

### Whole miRNA Expression Characters in PYTZ

To figure out the whole miRNAs express patterns in the spring sprouts of PYTZ, we need to evaluate the reliability of parallel experimental results as well as operational stability. The expression level of all miRNAs including known miRNAs and novel miRNAs from 15 libraries was normalized to generate TPM, which further used to compute the related coefficients. The strong correlation between every two biological replicates for interlibrary of all five sample groups brought out that the sequencing results are highly reliable (Supplementary Figure 3). The correlations between sL1 and sL2, sS1, and sS2 were substantially higher than other inter-groups, suggesting closely associated integral processes in the separate development of leaf and stem.

**Figure 3:**
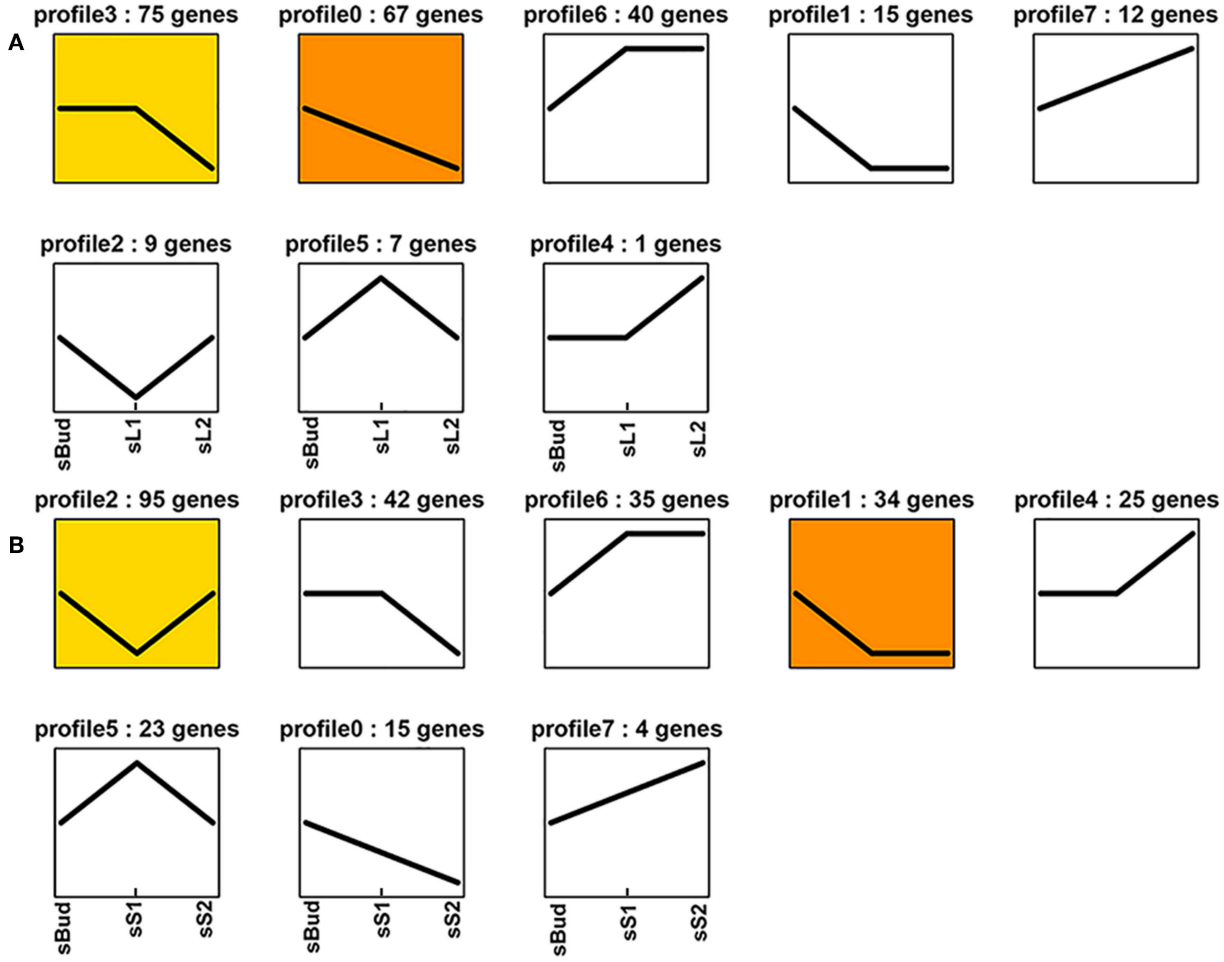
Expression profile trends of the whole miRNA in G1 **(A)** and G2 **(B)**.

To lock the target miRNAs which might be responsible for the tea shoot development, we firstly define sBud/ sL1/sL2 as Group 1 (G1), sBud/ sS1/sS2 as Group 2 (G2) to see the whole miRNA change characters. In G1, a total of 226 miRNAs showed different expression among the three tissues and classified into 8 profiles according to their trends. Overall, the down-expression trend miRNAs took up a larger percentage (69.5%), which belonged to profile 3 (75 miRNAs), profile 0 (67 miRNAs), and profile 1 (15 miRNAs). The up-expression trend miRNAs which had the most abundant expression in sL2 belonged to profile 6 (40 miRNAs), profile 7 (12 miRNAs), and profile 4 (1 miRNA). There were other 9 and 7 miRNAs which had the highest and lowest expression level in sL1, separately (Figure 3A).

In G2, a total of 273 miRNAs showed different expression among the three tissues and had been classified into 8 profiles according to their trends. Overall, 91 miRNAs showed the down-expression trend belonging to profile 3 (42 miRNAs), profile 1 (34 miRNAs), and profile 0 (15 miRNAs). 64 miRNAs showed the up-expression trend belonging to profile 6 (35 miRNAs), profile 4 (25 miRNAs), and profile 7 (4 miRNA). It is noteworthy that there were 95 miRNAs and 23 miRNAs showed the lowest and highest expression level in sS1, separately (Figure 3B).

### Conserved miRNAs Families and Tissue-Specific miRNAs

The 156 known miRNAs belonging to 125 families, among which 27 families were well-conserved that present in more than 10 plant species out of 72 plant species (Table 2). miR156 was the most popular one, which was found in 51 plant species, followed by miR396 and miR166, which were conserved in 47 and 45 plant species, respectively.

**Table 2:** 27 conserved miRNA families from PYTZ searched in other 72 plant species.

**Species**	**M IR 156**	**M IR 396**	**M IR 166**	**M IR 171**	**M IR 160**	**M IR 167**	**M IR 164**	**M IR 172**	**M IR 319**	**M IR 159**	**M IR 169**	**M IR 408**	**M IR 390**	**M IR 395**	**M IR 398**	**M IR 168**	**M IR 399**	**M IR 162**	**M IR 393**	**M IR 394**	**M IR 482**	**M IR 403**	**M IR 530**	**M IR 2111**	**M IR 477**	**M IR 535**	**M IR 2118**
*Brachypodium distachyon*	+	+	+	+	+	+	+	+	+	+	+	+	+	+	+	+	+	+	+	+	–	–	+	–	–	–	+
*Glycine max*	+	+	+	+	+	+	+	+	+	+	+	+	+	+	+	+	+	+	+	+	+	+	+	+	–	–	+
*Arabidopsis thaliana*	+	+	+	+	+	+	+	+	+	+	+	+	+	+	+	+	+	+	+	+	–	+	–	+	–	–	–
*Oryza sativa*	+	+	+	+	+	+	+	+	+	+	+	+	+	+	+	+	+	+	+	+	–	–	+	–	–	+	+
*Arabidopsis lyrata*	+	+	+	+	+	+	+	+	+	+	+	+	+	+	+	+	+	+	+	+	–	+	–	+	–	–	–
*Malus domestica*	+	+	+	+	+	+	+	+	+	+	+	+	+	+	+	+	+	+	+	+	+	+	–	+	+	+	+
*Prunus persica*	+	+	+	+	+	+	+	+	+	+	+	–	+	+	+	+	+	+	+	+	+	+	+	+	+	+	–
*Vitis vinifera*	+	+	+	+	+	+	+	+	+	+	+	+	+	+	+	+	+	+	+	+	+	+	–	+	+	+	–
*Populus trichocarpa*	+	+	+	+	+	+	+	+	+	+	+	+	+	+	+	+	+	+	+	+	+	+	+	+	+	–	–
*Solanum tuberosum*	+	+	+	+	+	+	+	+	+	–	+	+	+	+	+	–	+	+	+	–	+	–	+	–	+	–	–
*Citrus sinensis*	+	+	+	+	+	+	+	+	+	+	+	+	+	+	+	–	+	+	+	+	+	+	+	–	+	+	–
*Cucumis melo*	+	+	+	+	+	+	+	+	+	+	+	+	+	+	+	+	+	+	+	+	–	–	+	+	+	–	–
*Manihot esculenta*	+	+	+	+	+	+	+	+	+	+	+	+	+	+	–	+	+	+	+	+	+	+	+	+	+	+	–
*Amborella trichopoda*	+	+	+	+	+	+	+	+	+	+	+	–	+	+	+	+	–	–	+	+	–	–	–	+	+	+	–
*Medicago truncatula*	+	+	+	+	+	+	+	+	+	+	+	+	+	+	+	+	+	+	+	–	+	–	+	+	–	–	+
*Nicotiana tabacum*	+	+	+	+	+	+	+	+	+	+	+	+	+	+	+	+	+	+	–	+	+	–	–	–	+	–	–
*Aegilops tauschii*	+	+	+	+	+	+	+	+	+	–	+	+	+	+	+	+	+	–	+	+	–	–	–	–	–	–	+
*Zea mays*	+	+	+	+	+	+	+	+	+	+	+	+	+	+	+	+	+	+	+	+	+	–	–	–	–	–	+
*Sorghum bicolor*	+	+	+	+	+	+	+	+	+	+	+	+	+	+	+	+	+	+	+	+	–	–	–	–	–	–	+
*Theobroma cacao*	+	+	+	+	+	+	+	+	+	–	+	–	+	+	+	+	+	+	+	+	–	+	+	+	–	+	–
*Carica papaya*	+	+	+	+	+	+	+	+	+	+	+	+	+	+	+	–	–	+	+	+	–	–	–	–	+	+	–
*Linum usitatissimum*	+	+	+	+	+	+	+	+	+	+	+	+	+	+	+	+	+	+	+	+	–	–	+	–	–	–	–
*Ricinus communis*	+	+	+	+	+	+	+	+	+	+	+	+	+	+	+	+	+	+	+	–	–	+	–	–	–	+	–
*Solanum lycopersicum*	+	+	+	+	+	+	+	+	+	+	+	–	+	+	–	+	+	+	–	+	+	+	–	–	+	–	–
*Triticum aestivum*	+	+	–	+	+	+	+	–	+	+	+	+	–	+	+	–	+	–	–	–	–	–	+	–	–	–	–
*Aquilegia caerulea*	+	+	+	+	+	+	–	+	+	+	+	+	–	+	+	+	+	–	–	–	+	–	+	–	+	+	–
*Brassica napus*	+	+	+	+	+	+	+	+	–	+	+	–	+	+	–	+	+	+	+	+	–	+	–	+	–	–	–
*Brassica rapa*	+	+	–	+	+	+	+	+	+	+	–	+	+	+	+	+	–	+	–	–	–	+	–	+	–	–	–
*Cynara cardunculus*	+	+	–	+	+	+	+	+	+	–	+	+	+	+	+	+	+	–	+	+	–	–	–	–	–	–	–
*Physcomitrella patens*	+	–	+	+	+	+	–	–	+	–	–	+	+	+	–	–	–	–	–	–	–	–	–	–	+	+	–
*Gossypium hirsutum*	+	+	+	–	+	+	+	+	–	–	+	–	+	–	+	–	+	+	+	+	+	–	–	–	–	–	–
*Hevea brasiliensis*	+	+	+	–	–	–	–	–	+	+	–	+	–	–	+	–	–	–	–	–	+	–	–	–	–	–	+
*Vigna unguiculata*	+	–	–	–	+	–	+	+	+	–	+	+	–	+	–	+	+	+	–	–	+	–	–	–	–	–	+
*Gossypium raimondii*	–	–	+	–	–	+	+	+	–	–	–	–	–	–	+	–	+	–	–	–	+	–	+	–	+	–	–
*Salvia sclarea*	+	+	+	+	–	–	+	+	–	–	+	–	–	+	+	–	+	–	–	+	–	–	–	–	–	–	–
*Pinus taeda*	+	+	+	+	–	–	–	–	+	+	–	+	+	–	+	–	–	–	–	–	+	–	–	–	–	–	–
*Festuca arundinacea*	+	+	+	+	+	–	+	–	–	+	+	–	–	–	–	–	–	–	–	–	–	–	–	–	–	–	–
*Saccharum* sp.	+	+	+	–	–	+	–	–	–	+	+	+	–	–	–	+	–	–	–	–	–	–	–	–	–	–	–
*Arachis hypogaea*	+	–	–	–	+	+	–	–	–	+	–	+	–	–	+	–	–	–	–	+	–	–	–	–	–	–	–
*Helianthus tuberosus*	+	–	–	+	+	–	–	–	–	+	–	–	–	–	–	–	–	+	+	–	–	+	+	–	–	–	–
*Hordeum vulgare*	+	–	+	+	–	–	–	–	–	+	+	–	–	–	–	+	+	–	–	–	–	–	–	–	–	–	–
*Lotus japonicus*	–	+	–	+	–	+	–	+	–	–	–	+	+	–	–	–	–	–	–	–	–	–	–	+	–	–	–
*Pinus densata*	–	+	+	+	–	–	–	–	–	+	+	–	+	–	–	–	–	+	–	–	+	–	–	–	–	–	–
*Selaginella moellendorffii*	+	+	+	+	+	–	–	–	+	+	–	+	–	–	–	–	–	–	–	–	–	–	–	–	–	–	–
*Digitalis purpurea*	+	+	+	–	+	+	–	+	–	–	–	+	–	–	–	–	–	–	–	–	–	–	–	–	–	–	–
*Picea abies*	–	+	+	–	+	–	–	–	–	–	–	–	–	+	–	–	–	–	–	–	+	–	–	–	–	+	–
*Acacia auriculiformis*	–	+	–	–	+	–	–	+	+	–	–	–	–	–	–	+	–	+	–	–	–	–	–	–	–	–	–
*Citrus trifoliata*	+	–	+	+	–	+	+	–	+	–	–	–	–	–	–	–	–	–	–	–	–	–	–	–	–	–	–
*Phaseolus vulgaris*	–	–	+	–	–	–	–	–	+	+	–	–	–	–	–	–	+	–	–	–	+	–	–	–	–	–	+
*Saccharum officinarum*	+	+	–	–	–	+	–	–	–	+	–	+	–	–	–	+	–	–	–	–	–	–	–	–	–	–	–
*Brassica oleracea*	–	–	–	+	–	–	–	+	–	–	–	–	–	–	+	–	–	–	–	–	–	–	–	–	–	–	–
*Citrus clementina*	–	+	–	+	–	+	–	–	–	–	–	–	–	–	–	+	–	–	–	–	–	–	–	–	–	–	–
*Citrus reticulata*	–	–	+	+	–	–	–	–	–	–	–	–	–	–	–	+	–	–	–	–	–	–	–	–	–	–	–
*Cunninghamia lanceolata*	–	–	+	–	–	–	+	–	–	–	–	–	–	–	–	–	–	+	–	–	–	–	–	–	–	–	–
*Glycine soja*	–	–	–	–	–	+	–	–	–	–	–	–	–	–	–	–	–	–	–	–	+	–	–	–	–	–	–
*Helianthus annuus*	+	–	–	–	+	–	–	–	–	–	–	–	–	–	–	–	–	–	–	–	–	–	–	–	–	–	–
*Helianthus paradoxus*	+	–	+	+	–	–	–	–	–	–	–	–	–	–	–	–	–	–	–	–	–	–	–	–	–	–	–
*Helianthus petiolaris*	–	–	+	–	–	–	–	–	–	–	–	–	–	–	–	–	–	+	–	–	–	+	–	–	–	–	–
*Rehmannia glutinosa*	–	–	–	–	–	–	+	–	–	–	–	–	–	–	–	–	–	–	–	–	–	–	–	–	–	–	–
*Acacia mangium*	–	+	–	–	–	–	–	–	+	–	–	–	–	–	–	–	–	–	–	–	–	–	–	–	–	–	–
*Avicennia marina*	+	+	–	–	–	–	–	–	–	–	–	–	–	–	–	–	–	–	–	–	–	–	–	–	–	–	–
*Bruguiera cylindrica*	+	+	–	–	–	–	–	–	–	–	–	–	–	–	–	–	–	–	–	–	–	–	–	–	–	–	–
*Bruguiera gymnorhiza*	+	+	–	–	–	–	–	–	–	–	–	–	–	–	–	–	–	–	–	–	–	–	–	–	–	–	–
*Helianthus argophyllus*	+	–	–	–	–	–	–	–	–	–	–	–	–	–	–	–	–	–	–	–	–	+	–	–	–	–	–
*Helianthus ciliaris*	+	–	–	–	–	–	+	–	–	–	–	–	–	–	–	–	–	–	–	–	–	–	–	–	–	–	–
*Panax ginseng*	–	–	–	–	–	–	–	–	–	–	–	–	–	–	–	–	–	–	–	–	+	–	–	–	–	–	+
*Chlamydomonas reinhardtii*	–	–	–	–	–	–	–	–	–	–	–	–	–	–	–	–	–	–	–	–	–	–	–	–	–	–	–
*Elaeis guineensis*	–	–	–	–	–	–	–	+	–	–	–	–	–	–	–	–	–	–	–	–	–	–	–	–	–	–	–
*Gossypium herbaceum*	–	–	–	–	–	–	–	–	–	–	+	–	–	–	–	–	–	–	–	–	–	–	–	–	–	–	–
*Helianthus exilis*	–	–	–	–	–	–	–	–	–	–	–	–	+	–	–	–	–	–	–	–	–	–	–	–	–	–	–
*Populus euphratica*	–	–	–	–	–	–	–	–	–	–	–	–	–	–	–	–	–	–	–	–	–	–	–	–	–	–	–
*Triticum turgidum*	–	–	–	–	+	–	–	–	–	–	–	–	–	–	–	–	–	–	–	–	–	–	–	–	–	–	–
Sum	51	47	45	43	41	40	37	37	37	36	36	34	33	33	33	32	32	31	26	26	23	17	16	15	15	13	12

The expression of miRNA usually tells more about the code of regulating new shoot elongation and development. In order to filter the tissue-specific miRNAs in PYTZ, we merged the DEM from sL1 and sL2 into sL, sS1 and sS2 into sS, and removed duplicates, separately. The Venn diagram (Figure 2B) showed that most miRNA were existed in all tissues (82.49%) or at least in one tissue, regardless of their relatively high or medium expression abundance. Interestingly, some miRNA could only have their expression in specific tissues. Five miRNAs were bud-specific that could only be expressed in the bud, 31 were leaf-specific, and 28 were stem-specific, which had been summarized in Table 3.

**Table 3:** Tissue specific miRNAs of PYTZ.

**Tissue specific**	**miRNAs of PYTZ**
Bud	miR1128-x, miR781-y, miR8590-y, miR870-y, miR9759-y
Leaf	miR1042-x, miR1045-x, miR1057-y, miR1063-x, miR1515-x, miR164-y, miR1866-y, miR2083-y, miR2111-y, miR2275-y, miR393-x, miR5061-y, miR5181-y, miR5523-y, miR5538-x, miR5653-y, miR6173-y, miR6281-x, miR7528-y, miR7711-x, miR7725-x, miR845-z, miR858-y, miR8665-y, miR9569-x, novel-m0703-5p, novel-m0722-5p, novel-m0945-3p, novel-m0953-5p, novel-m1089-3p, novel-m1125-5p
Stem	miR1127-x, miR1865-x, miR2275-x, miR3512-y, miR3630-x, miR4388-y, miR474-x, miR474-y, miR5021-x, miR5385-x, miR5834-x, miR6485-x, miR7713-x, miR7717-x, miR7762-y, miR8007-y, miR8681-y, miR9863-x, novel-m0024-3p, novel-m0095-3p, novel-m0155-3p, novel-m0331-5p, novel-m0392-5p, novel-m0828-3p, novel-m0906-3p, novel-m1037-3p, novel-m1040-3p, novel-m1041-5p

### Differentially Expressed miRNAs (DEM)

The differentially expressed miRNAs (DEM) were pairwise compared among sBud, sL and sS, with their expression values higher than a 2-fold change and p ≤ 0.05, aiming to find out key miRNAs during the development. The numbers of DEM between tissues were summarized in Figures 2C–G. It's worth noting that according to the developmental order, there was a sharp down-trend numbers of DEM (235) and 79 up-trend DEM in sL1 than in sS1 (referred to as sL1 vs. sS1), compared to 67 down-trend and 5 up-trend DEM in sL1 vs. sL2, reminding that there are distinct regulatory changes and thus a metabolites accumulate differences bounded between L1 and S1 (Figure 2C). DEM with ups and downs both in G1 (Figures 2D,F) and G2 (Figures 2E,G) were also classified. The continuously changing ones were seemed to be possibly interesting regulators, such as 5 DEM (miR390-x, novel-m0578-5p, novel-m0634-5p, novel-m0503-3p, novel-m0531-5p) that had downtrend expression in G1 (Figure 2F), miR396-x uptrend in G1 (Figure 2D) and novel-m0331-5p (Figure 2E) uptrend in G2.

### GO Enrichment and KEGG Pathway Analyses of DEM

miRNA sequences were searched against tea tree genomic sequences using the plant miRNA potential target finder to predict target mRNAs. The annotation of the target unigene of DEMs was conducted based on GO enrichment and KEGG analyses. In this study, a total of 5501 potential unigenes were predicted to be targeted by 934 miRNAs, including 138 conserved and 796 novel miRNAs. Among the miRNA, miR5385-x targeted the most unigenes (770), followed by miR5658-x (533), and miR8577-x (223). 280 miRNAs targeted one unigene, while most miRNAs could target multiple sites. Similarly, one unigene was also targeted by several miRNAs, and there were 1351 unigenes could be regulated by more than one miRNA, 58 of which were targeted by no <10 conserved miRNA. (The complete list of target genes of all miRNA were listed in Supplementary Table 8).

Gene Ontology (GO) enrichment analysis offered a strictly defined concept to describe properties of the target genes and recognize the main biological functions in a dynamic-updated controlled vocabulary. Within biological process categories, represented GO terms associated with these target genes in all tissues (Supplementary Figure 4) were related to “metabolic process” the most, followed by “cellular process” and “single-organismal process”. Within cellular components categories, the unigenes were similarly represented, mainly in “cell,” “cell part,” “membrane,” “organelle” and their parts. Within the molecular function categories, the top two GO terms were “catalytic activity” and “binding”.

### Key DEM Involved in Growth and Development

**Figure 4:**
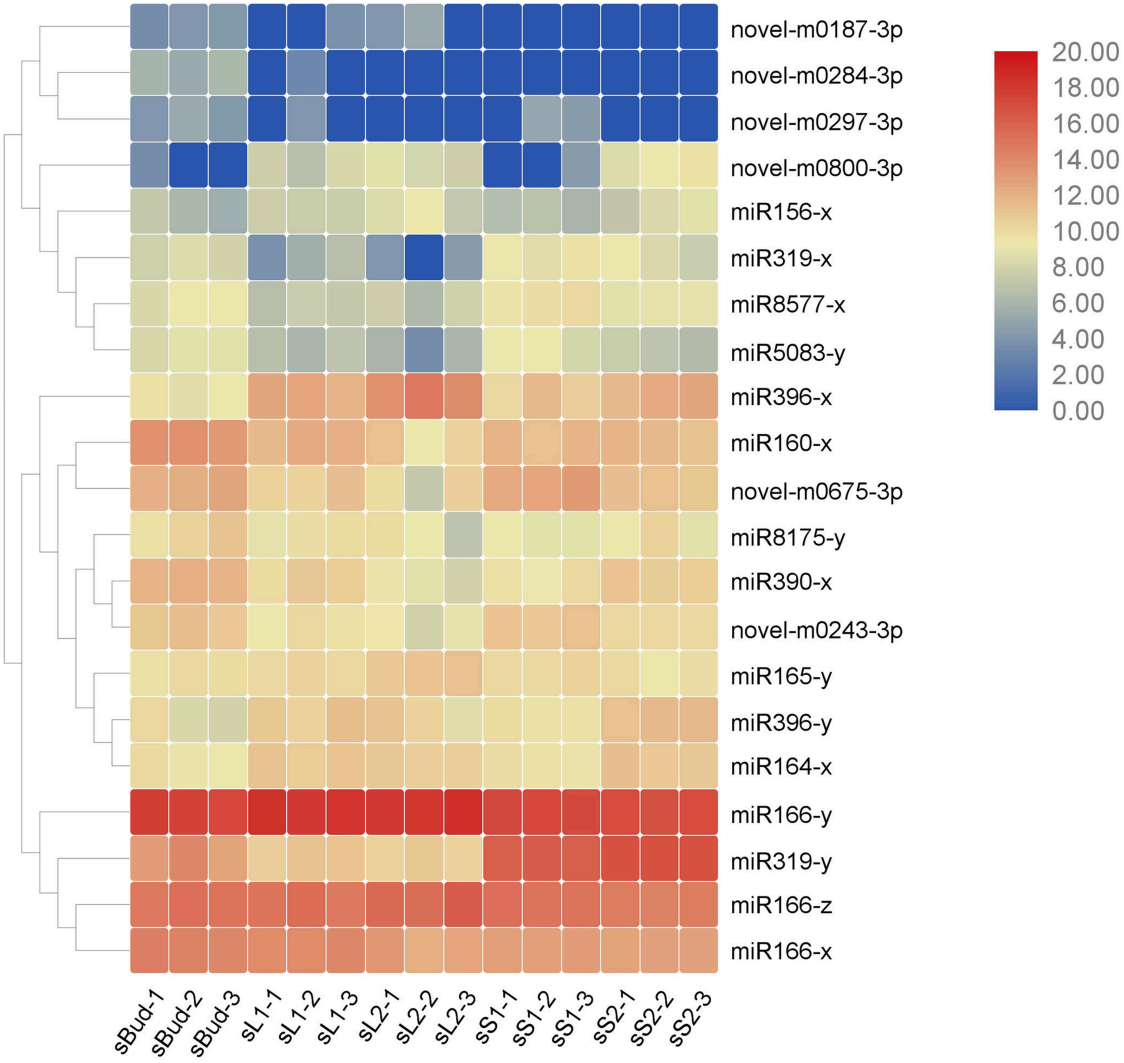
Heat map of the 21 miRNAs by the value of TPM in the 15 samples. The three major clusters on the left showed evolutionary relationships of the miRNAs, with closely related miRNAs neighboring clustered. Similar expression patterns could be found within one cluster.

We focused on the expressions of miRNAs to filter the possible ones that participate in growth and development, and we found that all of the reported growth and development associate miRNAs belong to the up- or down- trends pattern, except miR172. So we firstly narrowed down miRNAs with similar expression trends in the fore-mentioned G1 and G2 (in which the down-trends including profile 3, 0 and 1; the up-trends including profile 6, 7 and 4), and then further screened by GO and KEGG pathway analysis. Twenty one miRNAs, including 6 novel miRNAs were screened out to be potential developmentally important miRNAs in PYTZ (Table 4). Mature sequences of these miRNAs and their target genes in tea genome were also listed in Table 4. The heat map of the 21 miRNAs which represent their transcription levels calculated by TPM in the samples were displayed in Figure 4. Not each miRNA has the same expression pattern in G1 compared with that in G2: 3 miRNAs only changed in G1, with 1 uptrend and 2 downtrends; 4 miRNAs only changed in G2, with 2 uptrends and 2 downtrends; 11 miRNAs had the same trends in both G1 and G2, with 3 uptrends and 8 downtrends. Interestingly, miR319-y had a downtrend expression pattern in G1 and uptrend in G2. Some other miRNAs hadn't been included in these trends might not because of their expression trends or levels, but the difference of expressions among development stages was not significant (*P* < 0.05, log2-fold change ≥2).

**Table 4:** Potential developmentally important miRNAs in *Pingyang tezaocha*.

**miRNA**	**Expression pattern**	**Mature miRNA sequence (5^**′**^-3^**′**^)**	**Target mRNA ID in tea genome**
miR156-x	G2 up	CUGACAGAAGAGAGUGAGCAC	CSA007311 CSA009012 $\underline{\text{CSA}011373}$ CSA013149 CSA017838 $\underline{\text{CSA}019508}$ CSA020439 $\underline{\text{CSA}023442}$ $\underline{\text{CSA}031667}$
miR160-x	G1/G2 down	UGCCUGGCUCCCUGUAUGCCA	CSA021765 CSA026035 CSA026847
miR164-x	G1/G2 up	UGGAGAAGCAGGGCACGUGCA	CSA013362 CSA027185 CSA033493
miR165-y	G1 up	UCGGACCAGGCUUCAUCCCCU	$\underline{\text{CSA}023057}$ $\underline{\text{CSA}030874}$
miR166-x	G1/G2 down	GGAAUGUUGGCUGGCUCGAUG	CSA001544 CSA017082 CSA028252 CSA028940 CSA031362 CSA031363
miR166-y	-	UCGGACCAGGCUUCAUUCCCC	CSA030874
miR166-z	-	UCGGACCAGGCUUCAUUCCCU	CSA030874
miR319-x	G1down	GAGCUUCCUUCUGUCCACUU	CSA003974 CSA005021 CSA007451 CSA014848 CSA017867 CSA018024 CSA018464 CSA021711 CSA036427
miR319-y	G1down G2 up	UUGGACUGAAGGGAGCUCCCU	CSA002826 CSA013833 CSA031080 $\underline{\text{CSA}036087}$
miR390-x	G1/G2down	AAGCUCAGGAGGGAUAGCGCC	CSA001776 CSA001956 CSA006028 CSA007412 CSA011969 CSA016385 CSA022179 CSA026282 CSA030630 CSA036196
miR396-x	G1/G2 up	UUCCACAGCUUUCUUGAACUU	CSA003330
miR396-y	G2 up	GCUCAAGAAAGCUGUGGGAAG	CSA008398
miR5083-y	G1/G2 down	CUACAAUUAUCUGAUCAAA	CSA036373
miR8175-y	G2 down	UCCCCGGCAACGGCGCCA	CSA013921 CSA018936
miR8577-x	G1 down	UGAGAUGAUGAUCAUGAU	CSA008171 $\underline{\text{CSA}030921}$
novel-m0675-3p	G1/G2 down	GAAUAUGAUGAAUUUGAAUG	CSA006098
novel-m0297-3p	G1/G2 down	ACCCCUAACCCCAACACCCAAUC	CSA012411
novel-m0243-3p	G1/G2 down	GAUCAGGAUGAAGCAACAUU	CSA027591 CSA034664
novel-m0284-3p	G1/G2 down	UUGGGCUGGGCAGAAAUUGGGC	CSA016454
novel-m0800-3p	G1/G2 up	GUUCAGUGAAGCUGUGGAAAG	CSA010612 CSA028516
novel-m0187-3p	G2 down	AUUUCCCUUUCCAAAUUCCUU	CSA012066 CSA024775

*The underlined genes are the targeted mRNA belonged to TF genes. The gray shaded ones are the genes have reciprocal expression profiles with responding miRNAs*.

### Potential Transcription Factor Target Genes

**Figure 5:**
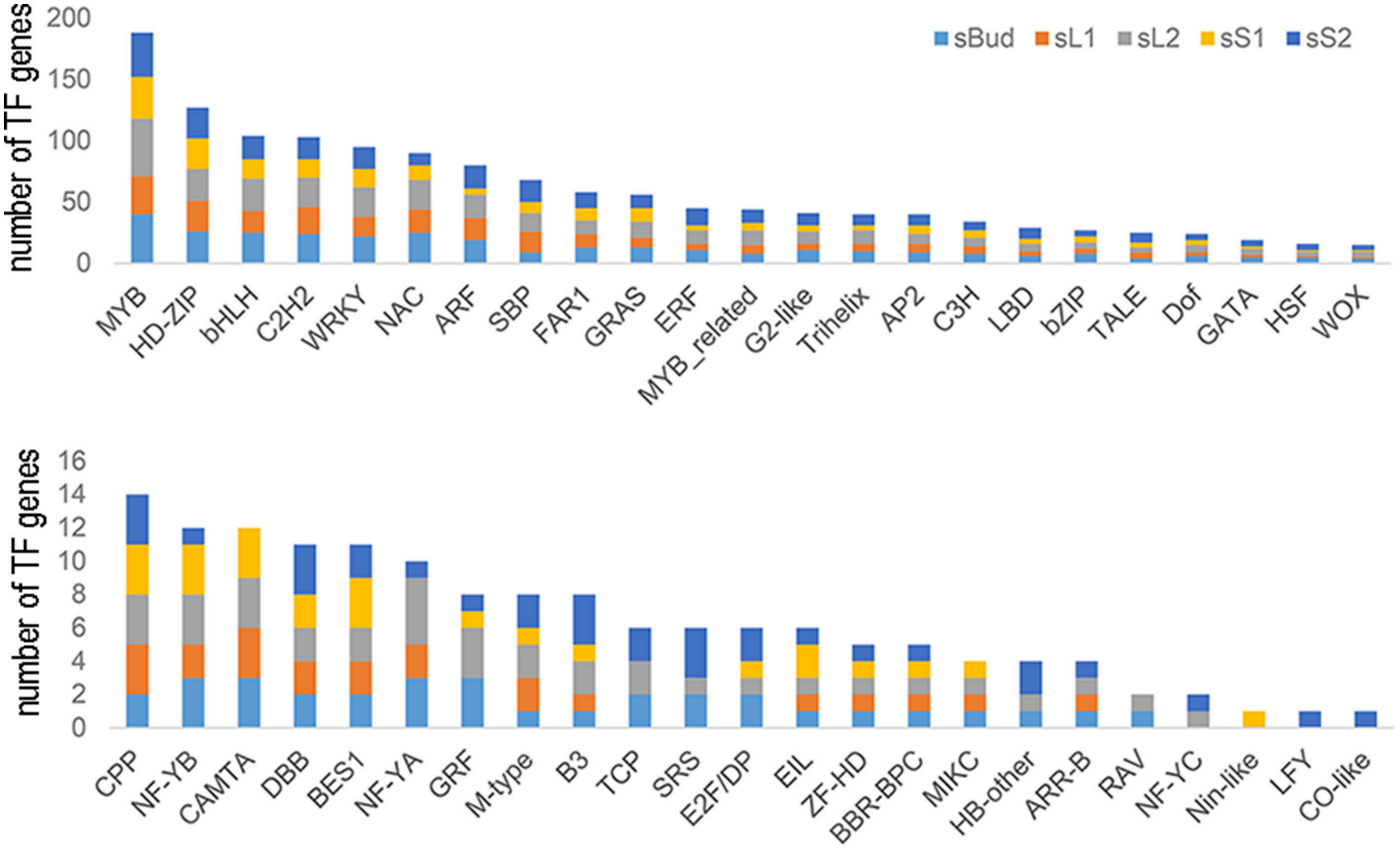
Types and numbers of transcription factors (TFs) genes targeted by miRNAs in each tissue.

As transcription factors were intensely studied in their numerous important roles during plant growth and development in many species (Ramachandran et al., 1994; Zhang et al., 2009; Chen et al., 2010), we are here supposed to analysis transcription factor genes for identifying key TFs performing this function. All the 5,501 predicted target genes were blasted against Plant Transcription Factor Database (http://planttfdb.cbi.pku.edu.cn/), resulting in a total of 46 kinds of transcription factors involving 352 mRNAs were detected (The full list of identified TFs was provided in Supplementary Table 9). Then, types and numbers of TFs genes targeted by miRNAs in each tissue (sBud, sL1, sL2, sS1, and sS2) were analyzed and summarized in Figure 5. On the whole, the numbers of transcription factors genes targeted by miRNAs in sL2 were the most, and that in sS1 was the least. MYB showed the widest involvement, followed by HD-ZIP and bHLH (basic Helix-Loop-Helix) transcription factors. Some kinds of transcription factors couldn't be targeted in all tissues, such as that the CAMTA family of calmodulin binding TF genes in sS2 and GRF (Growth Regulating Factor) in sL1.

**Figure 6:**
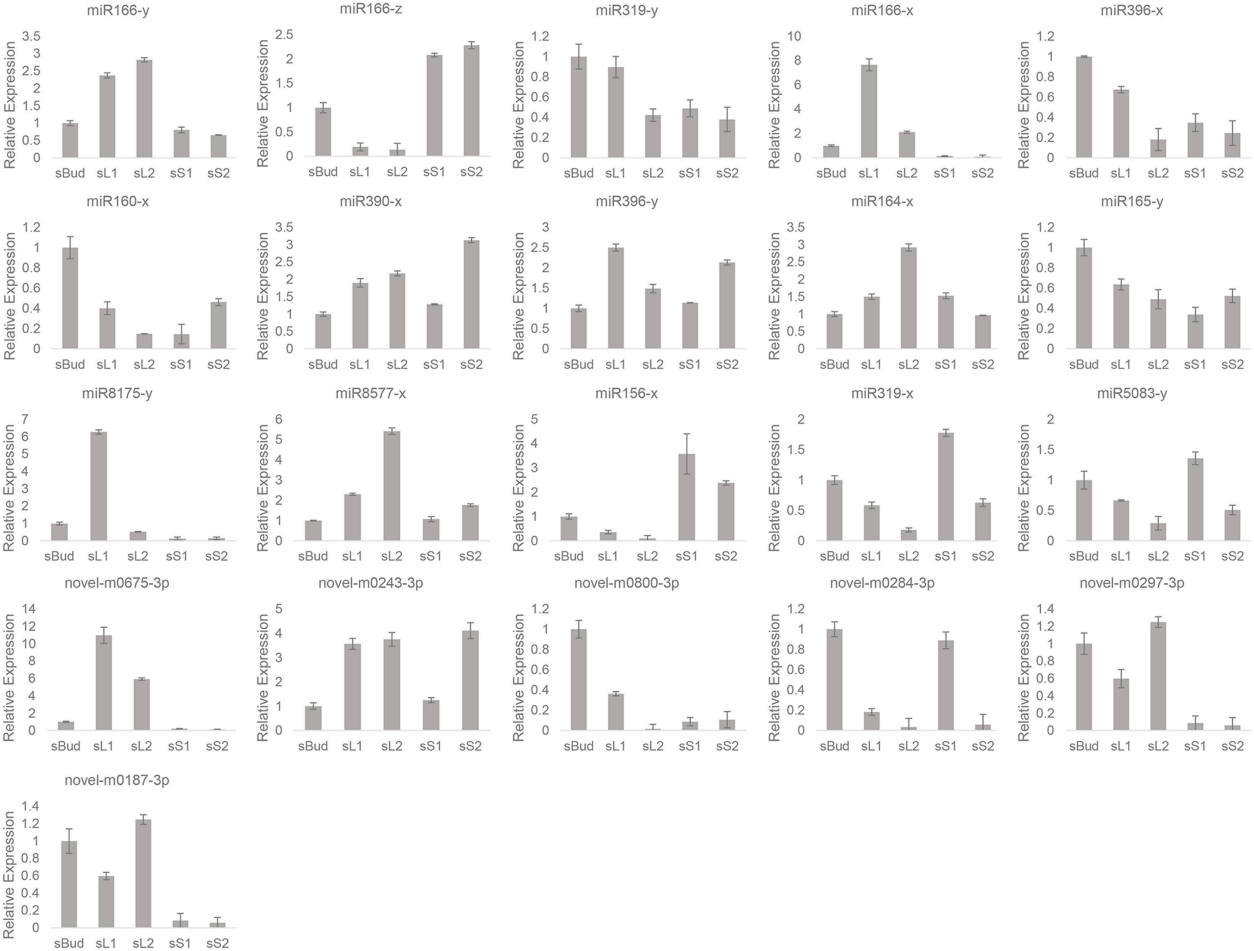
Relative expressions of the 21 miRNA by quantitative PCR.

**Figure 7:**
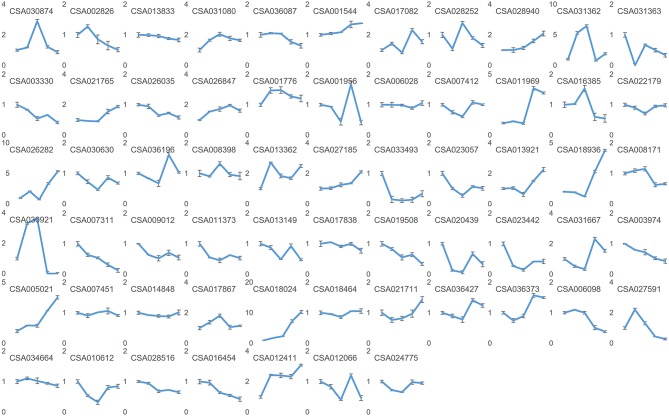
Relative expressions of the predicted target mRNA genes by quantitative PCR. Nodes on axis X for each inset image from left to the right are: sBud, sL1, sL2, sS1, and sS2. Designations the same as on previous figure.

Quantitative PCR was further performed to validate the mentioned 21 growth and development associate miRNAs (Figure 6) and their predicted target mRNAs (Figure 7), among which 14 of them were TF genes (underlined in Table 4). Universally, plant miRNAs might be involved in many complicated and diverse functions in the complex regulatory networks, the fundamental role of miRNAs is to suppress the expression of target genes (Tang and Chu, 2017). Herein, we got eight miRNA-TF genes pairs with reciprocal expression profiles (gray shaded mRNAs in Table 4), and the complementary correspondence of miRNA toward the target sites were shown in Supplementary Figure 5. The miRNA-TF genes pairs were miR156-x-CSA011373 (SBP), miR156x-CSA019508 (SBP), miR156x-CSA023442 (SBP), miR156x-CSA031667 (SBP), miR165y-CSA023057 (Class III HD-Zip), miR165y-CSA030874 (HD-Zip), miR319y-CSA036087 (MYB), and miR8577x-CSA030921 (bHLH).

### Expression Profiles of Reported Morphological miRNA in Different Tea Varieties

**Figure 8:**
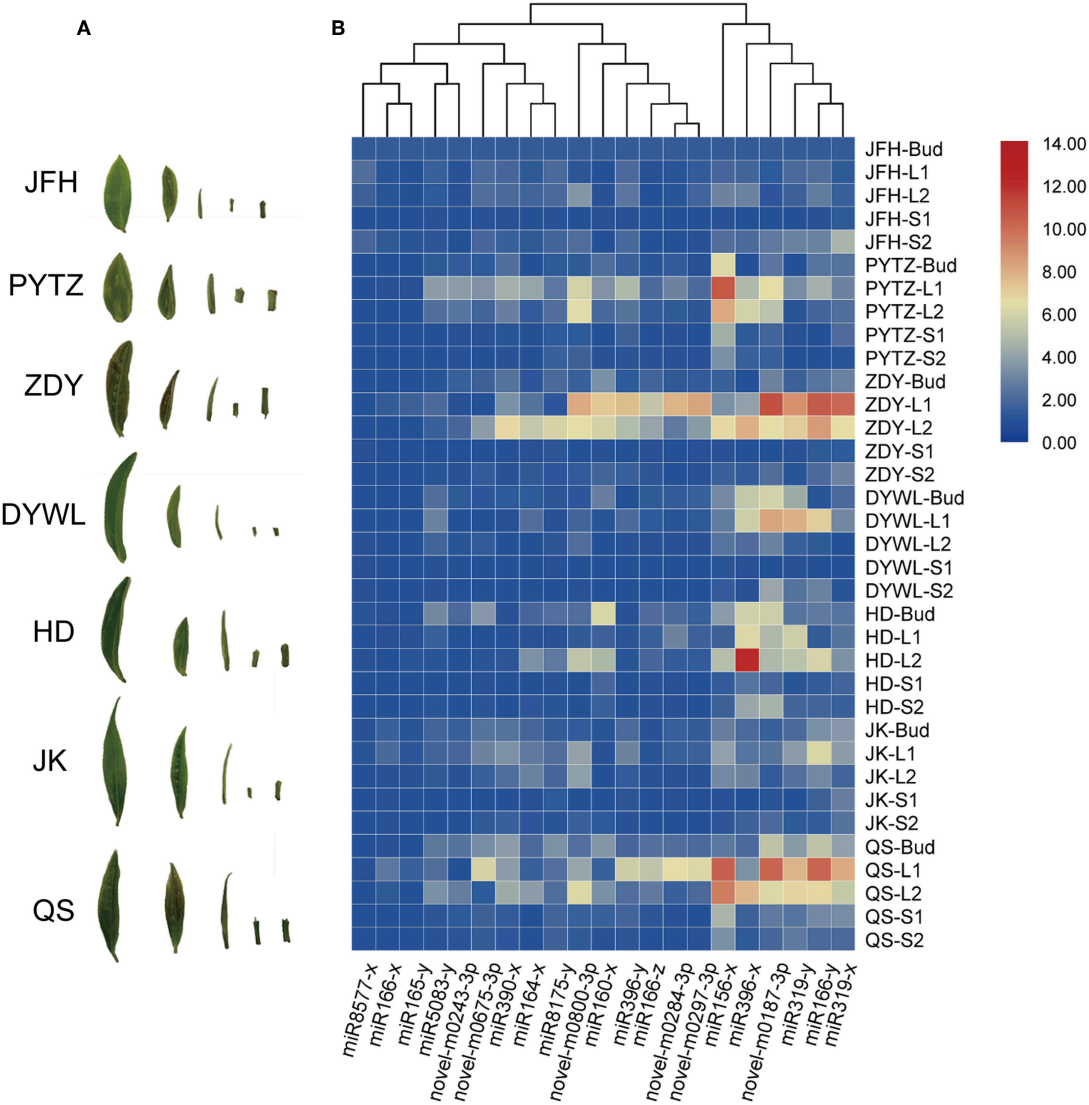
Heat map of the relative expression levels of 21 miRNAs in different tissues by Real-time PCR. **(A)** Samples from tea tree cultivars corresponding to **(B)**. Heat map of the relative expression levels of 21 miRNAs in different tissues from the *Camellia sinensis* “Jinfenghuang” (JFH), *Camellia sinensis* “Pingyang Tezaocha” (PYTZ), *Camellia sinensis* “Zhengdayin” (ZDY), *Camellia sinensis* “Dayewulong” (DYWL), *Camellia sinensis* “Huangdan” (HD), *Camellia sinensis* “Jiukeng 6” (JK), *Camellia sinensis* “Queshe” (QS).

Originated in Yunnan and Tibet region of China, the tea tree has been evolved over thousands of years and now at least 246 cultivars have been selected breeding with significant differences in morphology and physiology. Such as the focused cultivar in this study, PYTZ, has oblong leaf shape, blunt tip, tight dentate margins, and shorter internode. These characteristics are one of the indicators of screening and distinguishing cultivars. Plant growth and development are accompanied by morphogenesis that some regulators including small RNAs and TFs may participate in both biological processes simultaneously. In order to do some basal research linking developmental associate miRNA toward morphology, the expression profiles of reported morphological miRNAs were performed in other six tea varieties with obvious differences in leaf morphology. The focused 21 miRNAs were again checked by quantitative PCR for their expression levels in several representative cultivars. Bud, the 1st leaf, the 2nd leaf, the younger stem, and the older stem were also sampled from each variety in each group for quantitative PCR analysis (Figure 8). For each miRNA, the relative expression in JFH was set as the reference so as to get better understanding of the expression levels among varieties. In general, most of the miRNAs had similar expression patterns in ZYD, PYTZ, DYWL, and QS, with high expression levels in the 1st leaf and then the 2nd leaf. And for other miRNAs were likely to have high expression levels in the bud, such like miR160x in HD and miR156y in JFH.

### Network Analysis on miRNA, Target mRNA, and Quality-Related Metabolites

**Figure 9:**
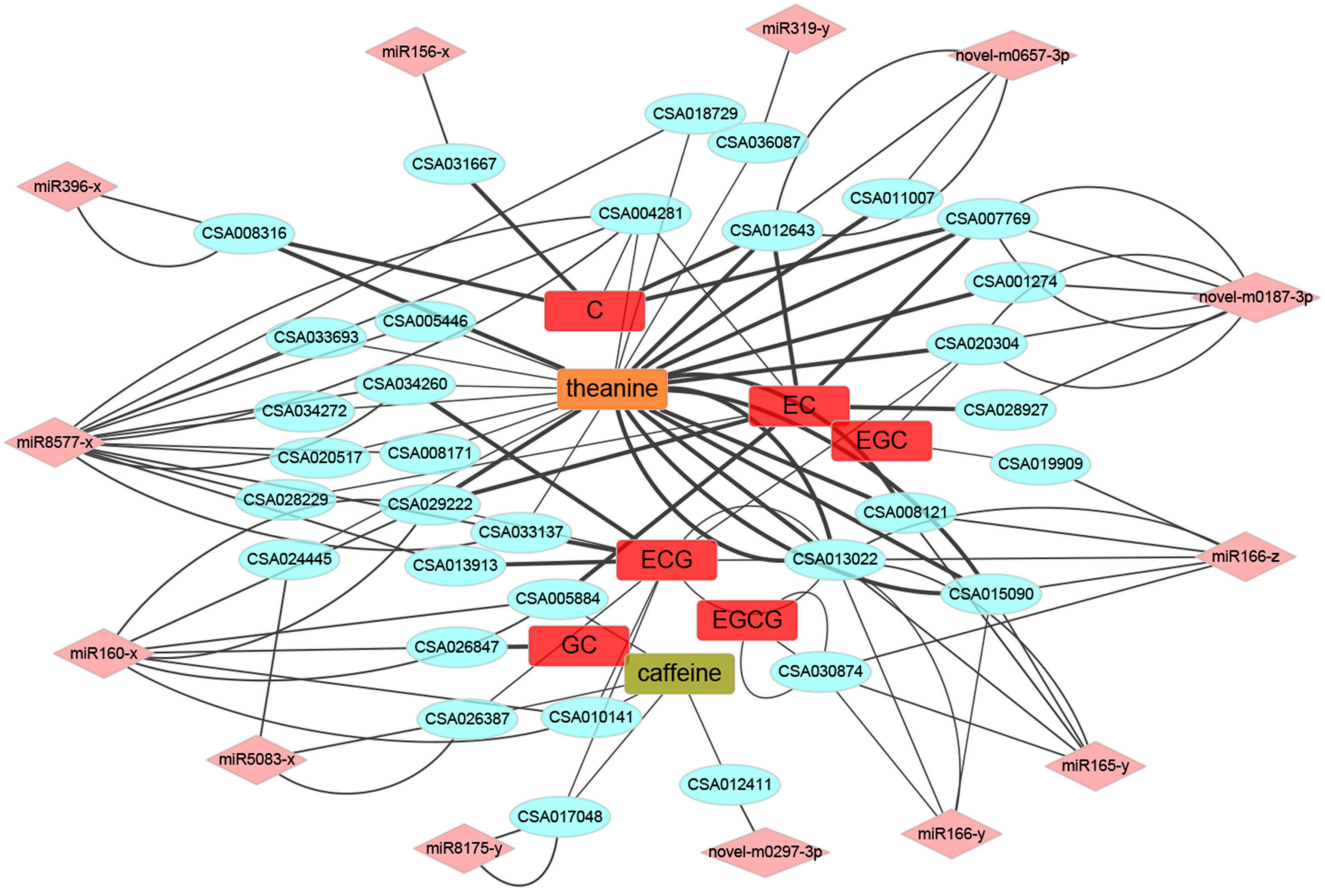
The miRNA-mRNA-metabolites association networks. The round rectangle placed in the center were several metabolites, the ellipses at the interlayer were potential target mRNAs, and the diamond at the outermost layer were miRNAs. Line thickness represented the strength of the relationship.

Correlation analyses were conducted to figure out the extent of the 21 conserved miRNA mentioned above participated in the quality formation progress (Figure 9). Surprisingly, compared to catechins and caffeine, theanine had a quite strong relationship and relatively large numbers with target mRNAs, which could be definitely set as the center regulated metabolite, at least during the development of PYTZ sprouts. For the galloylated catechins, ECG was regulated by much more multiple mRNAs than EGCG. The kinds of mRNA participated in regulating the nongalloylated catechins were approximately equal. As for the conserved miRNA, miR8577-x, miR160-x, and novel-m0187-3p were the most social ones. However, the neighborhood relationships for miR156-x and miR319-y seemed rather simple.

## Discussion

### Different miRNAs Were Involved in Different Tissues and Stages During the Sprout Development in PYTZ

How a plant builds leaves from a few cells that grow, divide, and differentiate to form into the complex organ has been well-studied, the same with the research of mechanical regulation (Braybrook and Kuhlemeier, 2010; Chen, 2012; Qi et al., 2017). miRNA, typically multigene families, allowing for subtlety and complexity of control in different regulatory processes, are described as factors in many aspects of plant development (Kidner, 2010).

As tea is the leaf-using crash plant, the performance of leaf development has more practical significance. Thus, we took the perspective of looking at the function of miRNA in different tissues and development stages separately. In the comparison of G1 and G2 mentioned above, miRNAs that participate in leaf and stem development are different, hinting that different miRNAs need coordinate working in leaf and stem developmental process, separately. For example, both novel-m0155-3p and novel-m0331-5p were stem-specific miRNAs and have high expression levels in sS2 than in sS1, which had strong possibilities responsible for stem elongation. And likewise, one kind of miRNA may function differently in different tissues development. In the 78 miRNAs that both exist in leaf and stem (Figure 2B), 28 of them have different including miR5049y and other 27 novel miRNAs could be found both in leaf and stem, in which 23 of them share one similar expression pattern (Supplementary Figure 6) (*P* < 0.05, log2-fold change ≥ 1.5), 27 of them with the expression levels in sL1 higher than in sL2 (Supplementary Figures 6A–C), 24 of them with the expression levels in sS2 higher than in sS1 (Supplementary Figures 6A,D). The non-conserved miR5049, included in profile1 (Supplementary Figure 6A), had been reported to be drought stress response miRNA in the root of drought-tolerant cultivar wheat (Akdogan et al., 2016).

Interestingly, the number of down-trend miRNAs (157 from profile 3, profile 0 and profile 1) are much more than up-trend ones in G1 (53 from profile 6, profile 7 and profile 4) (Figure 3A) and in G2 (91 vs. 64) (Figure 3B), this is the same case for the abundance of these miRNAs. Discarding the ones with TPM lower than 100, the abundance of down-trend miRNAs occupied 79.04% and 54.30% in G1 and G2, separately (Supplementary Table 10). The percentage indicated that during the development, especially in leaf, mRNAs regulated by miRNA have a large percentage in increasing tendency. This trend is consistent with the expression levels from tea leaf transcriptome that 72% of genes were up-regulated in the second leaf stage compared to the first leaf stage (Guo et al., 2017). The coherence of expression levels of the regulator and the content of secondary metabolites is particularly impressive, a point we return to below.

### Evolutionarily Conserved miRNAs Were Closely Connected to Morphogenesis Functions During the Sprout Development

miRNA is usually be concerned whether to be conserved or not, which typically depends on their degree of presentation in all or at least most of the species, and thus the division could be influenced by sampling and the phylogenetic diversity of available species that miRNAs have been characterized and annotated (Baldrich et al., 2018). In the 21 developmental associated miRNAs filtered out in this study (Table 4), except for 6 novel miRNAs, 11 of the known miRNAs are conserved miRNAs that could be found in at least 33 plant species out of 72 plant species (Table 2). Notably, none of the miRNAs from tea could be found in *Chlamydomonas reinhardtii*, which is a conventionally model for a photosynthetic cell in studying photosynthesis (Funes et al., 2007), abiotic stress (Hema et al., 2007), circadian clock (Ral et al., 2006) and so on. This result to some extent is an echo of the conservation of miRNA within one kingdom, and no miRNA had been found conserved in green algae and land plants (Baldrich et al., 2018). Interestingly, there was no tea miRNA found in *Populus euphratica* either, which is an ideal model system of woody plants for research into the abiotic stress resistance (Li et al., 2009), such like drought (Li et al., 2011) and salt (Li et al., 2013). Previously studies on *Populus euphratica* had been reported that only 9 out of 21 miRNAs families (miR156; miR163; miR172; miR398; miR393; miR171; miR408; miR169; miR472) were conserved in other plants, with other 12 miRNA family candidates show none homologies in *Populus, Arabidopsis*, and *Oryza* (Li et al., 2009) and can thus be considered as quite ancient and independent evolution species.

The seven tea cultivars studied above were famous cultivars that frequently used in producing fermented or non-fermented tea in China. Typically in the tea processing industry, tea-processing suitability and tea quality are basically determined by the main characteristic metabolic compounds, which directly linked up with the development and morphogenesis of the tea sprouts (Xia et al., 2017). In the 11 known conserved tea miRNA, miR156 was the most popular one, which was found in 51 plant species, followed by miR396 and miR166, which were conserved in 47 and 45 plant species, respectively. Similar with their high abundance (miR166, miR319, miR396, miR160, and miR390 were listed in the top five kinds of miRNAs), which means that conservation is not only represent low sequence variation across diverse plant species, but also to be the large and older miRNA families with abundant copy and target number (Chavez Montes et al., 2014), in order to grantee their tightly constrained roles in function and less gene loss in the regulatory network (Shi et al., 2017). Mature miRNAs in plant often have multiple target genes with similar complementary sequences, among which these evolutionarily conserved miRNAs and their predominantly target genes characteristically play essential roles in developmental regulation, morphogenesis, stress responses (Axtell and Bowman, 2008; Yang et al., 2013). Even more noteworthy is, the tissue-specific miRNAs (Table 2) may contribute to the development of the specific tissue, which doesn't mean they are the dominated ones and conserved ones, either. For example, miR319 had been reported to increase the number of longitudinal small veins thus might account for the leaf blade width (Yang et al., 2013) and miR159 was involved in stem elongation (Tsuji et al., 2006), but they are both conserved miRNAs and have expression in bud, leaves, and stems. Only miR164, miR393 and miR2111 were leaf-specific miRNAs and conserved miRNAs as well. Not all of the conserved miRNAs have similar expression patterns in the investigated cultivars (Figure 8), which might be the result of the flexible of the “fine-tuners,” to enhance the ability of a fast response to evolution (Muleo, 2012). The conservation in sequence doesn't always represent functional conservation (Ason et al., 2006). Though the evidence of miRNA in the plant is less than that in the animal, it is widely accepted that plants miRNA genes are evolved independently as they do in the animal kingdoms. It is thus believed that the larger the miRNA family is, which means the more multiple paralogous copies of one miRNA in plants, brings more flexible of evolution rate and more possibility in diversification though, the more essential function in development it may be involved. More research combined the contents of metabolites with the function of conserved miRNAs in species-level phenotypic differences needs to be further studied.

### Development Associate miRNAs Might Play Crucial Roles in the Quality Formation Together With Their Potential Target Transcription Factor Genes

Higher plants evolved precise and robust spatio-temporal patterns of gene regulatory systems, among which transcription factors and miRNAs are two of the best studied regulatory mechanisms separately at transcriptional and post-transcriptional level (Chen and Rajewsky, 2007). TFs and miRNAs generally do not work isolation, but instead, together with co-regulators in the same layer or not, to form large networks of cooperating and interacting in complex multicellular organisms (Dawid, 2006). But they are usually positioned at the center of regulating many aspects of developmental plasticity along with the life cycle (Rubio-Somoza and Weigel, 2011). We perform network and enrichment analysis to the 352 TF genes targeted by miRNA in STRING (Szklarczyk et al., 2017), and marked the developmental relative process (Supplementary Figure 7). Most of them were involved were clustered in some certain pathway in regulating gene expression and primary metabolic process, which could be easily understood that TFs were involved in the primary metabolic process because of the fundamental maintenance of living for plant themselves. In shrinking the research objectives, we further perform GO analysis to the DEM in G1 and G2 above, many pathways were enriched in developmental and morphological process (Supplementary Figure 8), especially in the G1 up trend expression profiles, which also confirmed us the effective way of filtering key miRNAs. The eight miRNA-TF mRNA pairs verified by their reciprocal expression relationships were much more likely to participate in development regulation, which doesn't imply only this eight pairs of miRNA-TFs were involved.

Metabolism was along with the development. It has been reported that plant miRNA were widely involved in quality formation regulation (Wu et al., 2014; Liu et al., 2017b). As the three kinds of characteristic metabolites which finally determine the quality of tea (Xia et al., 2017; Wei et al., 2018), catechins mainly confer astringent taste, theanine contributes to the umami and sweet tastes, and caffeine offers a bitter taste (Wei et al., 2018). We did GO pathway enrichment analysis to the target mRNA genes of DEM between every two samples from Bud, sL1, sL2, sS1, and sS2, with the evolved participants showed in Table 5. The correlation of the chemical analysis on catechins, theanine, caffeine and the soluble matter would finally affect the sensory evaluation of green tea taste. In our study, we found that theanine turned out to be even more active in the network (Figure 9). Target mRNAs which belonging to TF genes were further picked out and constructed a more metabolic directivity one due to their correlation (Supplementary Figure 9). TF genes like CSA013022 (HD ZIP) and CSA029222 (ARF) had a strong positive relationship with the biosynthesis of theanine, while the later also positively regulated EC, referring to miR165-y, miR166-z, and miR160-x, respectively. CSA031667, a TF gene belonging to SBP family, had a positive correlation with C, and be controlled by miR156-x. When taking the eight miRNA-TF pairs mentioned above into consideration together, there were at least two triplets that participate in both development and quality formation: miR156-x-CSA031667 (SBP)-C and miR319-y-CSA036087 (MYB)-theanine. Molecular mechanisms of sprout development and accumulation of metabolites would be gradually uncovered after the release of tea tree genome (Xia et al., 2017; Wei et al., 2018) and tea organic transcriptomes (Zheng et al., 2015, 2016; Guo et al., 2017; Liu et al., 2017a). More connections would be further studied toward small RNAs to improve breeding efficiency of developing better cultivars with higher quality.

**Table 5:** GO pathway enrichment analysis to the target mRNA genes of DEM between each two samples from Bud, sL1, sL2, sS1, and sS2.

**Characteristic pathway**	**Metabolic pathway**	**Annotated genes**	**Related mRNA**	**miRNA**
Flavonoid pathway	Phenylalanine metabolism	Amidase	CSA014307	miR8577-x
		Omega-amidase	CSA016772	novel-m0263-3p
	Phenylpropanoid biosynthesis	Cinnamyl-alcohol dehydrogenase (CAD)	CSA019604	novel-m0018-3p, novel-m0119-3p, novel-m0279-3p, novel-m0498-3p, novel-m0608-3p, novel-m0674-3p, novel-m0888-3p, novel-m0904-5p, novel-m0977-3p, novel-m1032-3p, novel-m1080-3p, novel-m1106-3p
			CSA005610	miR1511-y, miR5139-x, miR8155-y, novel-m0999-3p
			CSA009196	miR6118-y
		Peroxidase	CSA026001	miR5658-x, novel-m0675-3p
		Beta-glucosidase	CSA014637	novel-m0349-3p, novel-m0360-3p, novel-m0428-3p
		Shikimate O-hydroxycinnamoyltransferase (SHT)	CSA011696	miR8577-x
	Flavonoid biosynthesis	Naringenin 3-dioxygenase (F3H)	CSA004930	miR8577-x
		Chalcone isomerase (CHI)	CSA006623	novel-m0466-3p, novel-m0809-3p
	Anthocyanin biosynthesis	Anthocyanidin 3-O-glucoside 5-O-glucosyltransferase (UGT75C1)	CSA036671	miR1865-x, novel-m0674-3p, novel-m1032-3p
		UGAT	CSA013643	miR4995-x, miR6483-y
Caffeine Pathway	Caffeine Metabolism	Urate oxidase (UOX)	CSA020658	novel-m0021-5p, novel-m0508-3p, novel-m0698-3p, novel-m0834-3p, novel-m0964-3p, novel-m0964-3p
		Xanthine dehydrogenase/oxidase	CSA006612	miR5059-x
Amino acid Pathway	Citrate cycle (TCA cycle)	Isocitrate dehydrogenase (NAD+)	CSA018911	miR5083-y
		Pyruvate dehydrogenase E2 component (dihydrolipoamide acetyltransferase)	CSA034852	miR8175-y
		2-oxoglutarate dehydrogenase E1 component	CSA032631	novel-m0381-3p
		2-oxoglutarate dehydrogenase E2 component (dihydrolipoamide succinyltransferase)	CSA019997	novel-m0184-5p
		ATP citrate (pro-S)-lyase	CSA033096 CSA027464 CSA034373	miR5059-x
		Succinyl-CoA synthetase beta subunit	CSA027414	novel-m0018-3p, novel-m0119-3p, novel-m0279-3p, novel-m0498-3p, novel-m0608-3p, novel-m0674-3p, novel-m0888-3p, novel-m0904-5p, novel-m0977-3p, novel-m1032-3p, novel-m1080-3p, novel-m1106-3p
		Aconitate hydratase	CSA024268 CSA025056 CSA020729	novel-m0945-3p
		Fumarate hydratase, class II	CSA004158	novel-m0574-5p
			CSA036624	miR5054-y
		Succinyl-CoA synthetase beta subunit	CSA027414	novel-m0018-3p, novel-m0119-3p, novel-m0279-3p, novel-m0498-3p, novel-m0608-3p, novel-m0674-3p, novel-m0888-3p, novel-m0904-5p, novel-m0977-3p, novel-m1032-3p, novel-m1080-3p, novel-m1106-3p
	Alanine, aspartate and glutamate metabolism	Glutamate dehydrogenase (NAD(P)+)	CSA030852 CSA002178 CSA019055	novel-m0541-3p
		Alanine transaminase	CSA034511	novel-m0410-3p, novel-m0435-3p, novel-m0549-3p, novel-m0553-5p, novel-m0554-5p, novel-m0752-5p, novel-m0769-3p, novel-m0863-3p, novel-m0938-3p, novel-m1097-3p, novel-m1123-5p
		4-aminobutyrate—pyruvate transaminase	CSA023854	novel-m0074-3p, novel-m0075-3p
		Calcium-binding protein CML	CSA036463	novel-m1108-5p
			CSA009111	
		Amidophosphoribosyl transferase	CSA032114	miR5059-x, miR6281-x
		3-deoxy-7-phosphoheptulonate synthase	CSA021087	novel-m1042-3p
			CSA011007	novel-m0657-3p, novel-m1042-3p
		Arogenate/prephenate dehydratase	CSA006958	miR5054-y, novel-m0388-3p

## Data Availability

The datasets generated for this study can be found in Sprouts development of tea plants, PRJNA510482.

## Ethics Statement

The authors declare that we have complied with all relevant ethical regulations.

## Author Contributions

ZD conceived the study. LZ, CC, JS, and YW performed the experiment and analyzed the data. LZ wrote the paper. All authors read and approved the final manuscript.

### Conflict of Interest Statement

The authors declare that the research was conducted in the absence of any commercial or financial relationships that could be construed as a potential conflict of interest.

## Supplementary Material

Supplementary Figure 1 The nucleic acids frequency of known miRNAs at each position in PYTZ. sBud **(A)**, sL1 **(B)**, sL2 **(C)**, sS1 **(D)**, and sS2 **(E)**. The frequency of cytosine (C) (32.09%) and uracil (U) (29.65%) are higher than guanine (G) (19.26%) and adenosine (A) (19.00%). U had a high appearance at the 1st, 17th, 22th, and 23th positions, with an average of 84.34, 61.80, 54.57, and 52.48% respectively.

Supplementary Figure 2 The nucleotide bias of 18nt-30nt length known miRNAs at the 1st position in PYTZ. sBud **(A)**, sL1 **(B)**, sL2 **(C)**, sS1 **(D)**, and sS2 **(E)**.

Supplementary Figure 3 Related coefficients of the 15 miRNA libraries across the five sample groups with three replicates of each group.

Supplementary Figure 4 Level 2 GO terms of all target genes from PTZY sprouts.

Supplementary Figure 5 Complementary correspondence of developmental miRNA toward the target sites.

Supplementary Figure 6 **(A–E)** The expression profiles of miRNA that exist both in sL (leaf) and sS (stem).

Supplementary Figure 7 Relative process of the targeted TF genes by STRING analysis. (STRING: https://string-db.org/).

Supplementary Figure 8 Level 3 GO analysis of DEM in G1 and G2.

Supplementary Figure 9 The miRNA-TF-metabolites networks. The round rectangle placed in the center were metabolites, the ellipses at the interlayer were potential target mRNAs that belong to TF genes, and the diamond at the outermost layer were miRNAs. Line thickness represented the strength of the relationship. The red line represented the positive correlation efficient and the blue line meant negative.

Supplementary Table 1 The primers sequences of miRNA and mRNA genes used in Real-time PCR.

Supplementary Table 2 The filtering data of 15 sRNA-Seq libraries from *Camellia sinensis* cv. Pingyang Tezaocha.

Supplementary Table 3 Statistics of sRNA-Seq libraries mapping to tea tree genome.

Supplementary Table 4 Statistics of sRNA-Seq libraries mapping to Rfam.

Supplementary Table 5 The lengths, sequences, and expressions of 156 known miRNAs.

Supplementary Table 6 122 precursors and 99 kinds of characteristic hairpin structures, with their length and energy information.

Supplementary Table 7 1186 novel miRNAs and their 1130 hairpin structures, with their length and energy information.

Supplementary Table 8 The complete list of target genes of all miRNA.

Supplementary Table 9 The annotation and the corresponding miRNAs of the total 352 target transcription factor genes.

Supplementary Table 10 Down-trend and up-trend miRNAs in G1 and G2 with TPM more than 100.
