# Supplementary figures and images for: Conserved MicroRNA Act Boldly During Sprout Development and Quality Formation in Pingyang Tezaocha (Camellia sinensis)
Source: Front Genet. 2019 Mar 28;10:237. doi: 10.3389/fgene.2019.00237 (PMC6455055; doi:10.3389/fgene.2019.00237)

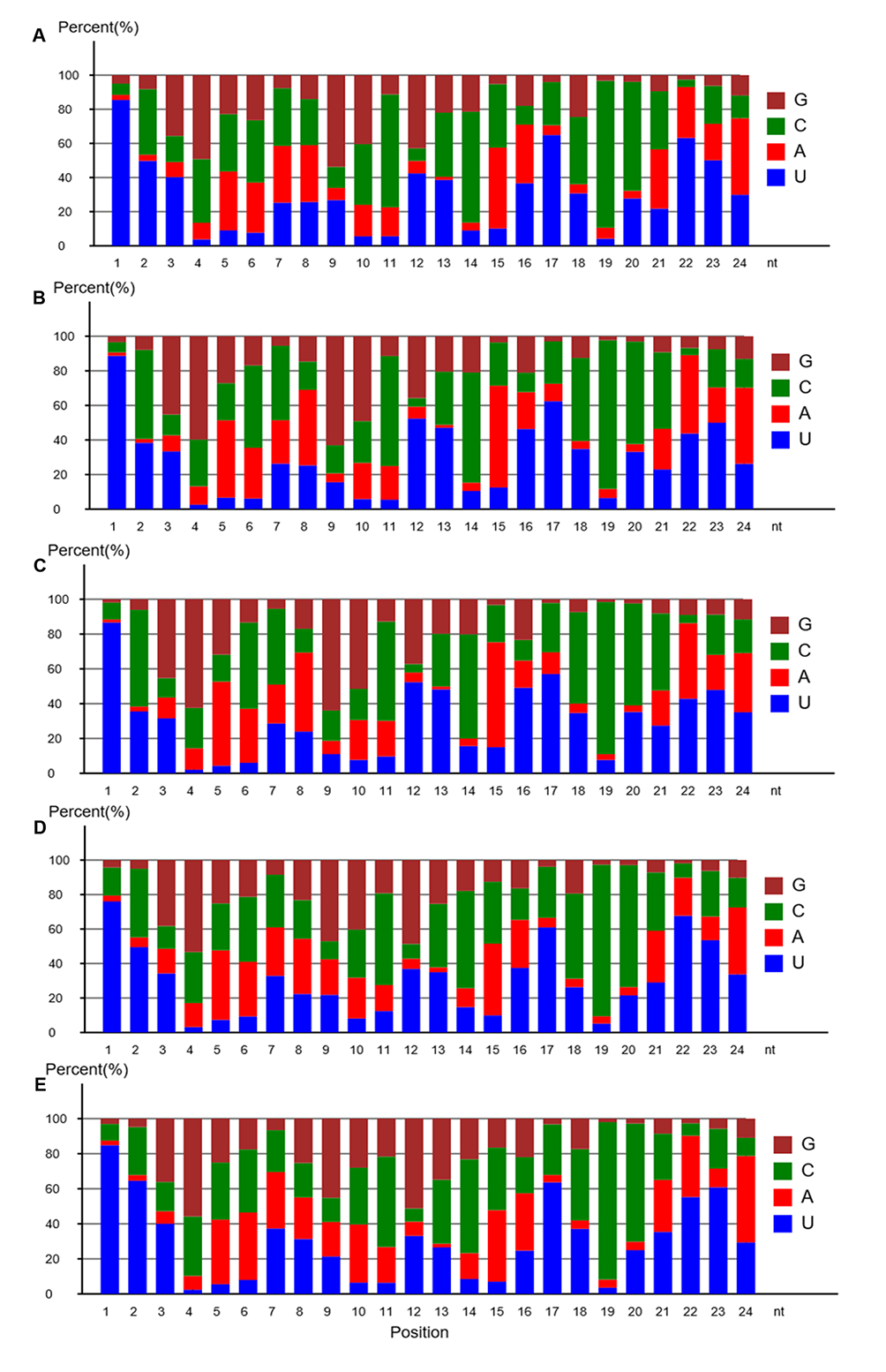

Supplement: Supplementary Figure 1 — The nucleic acids frequency of known miRNAs at each position in PYTZ. sBud (A), sL1 (B), sL2 (C), sS1 (D), and sS2 (E). The frequency of cytosine (C) (32.09%) and uracil (U) (29.65%) are higher than guanine (G) (19.26%) and adenosine (A) (19.00%). U had a high appearance at the 1st, 17th, 22th, and 23th positions, with an average of 84.34, 61.80, 54.57, and 52.48% respectively. [file Image_1.TIF]

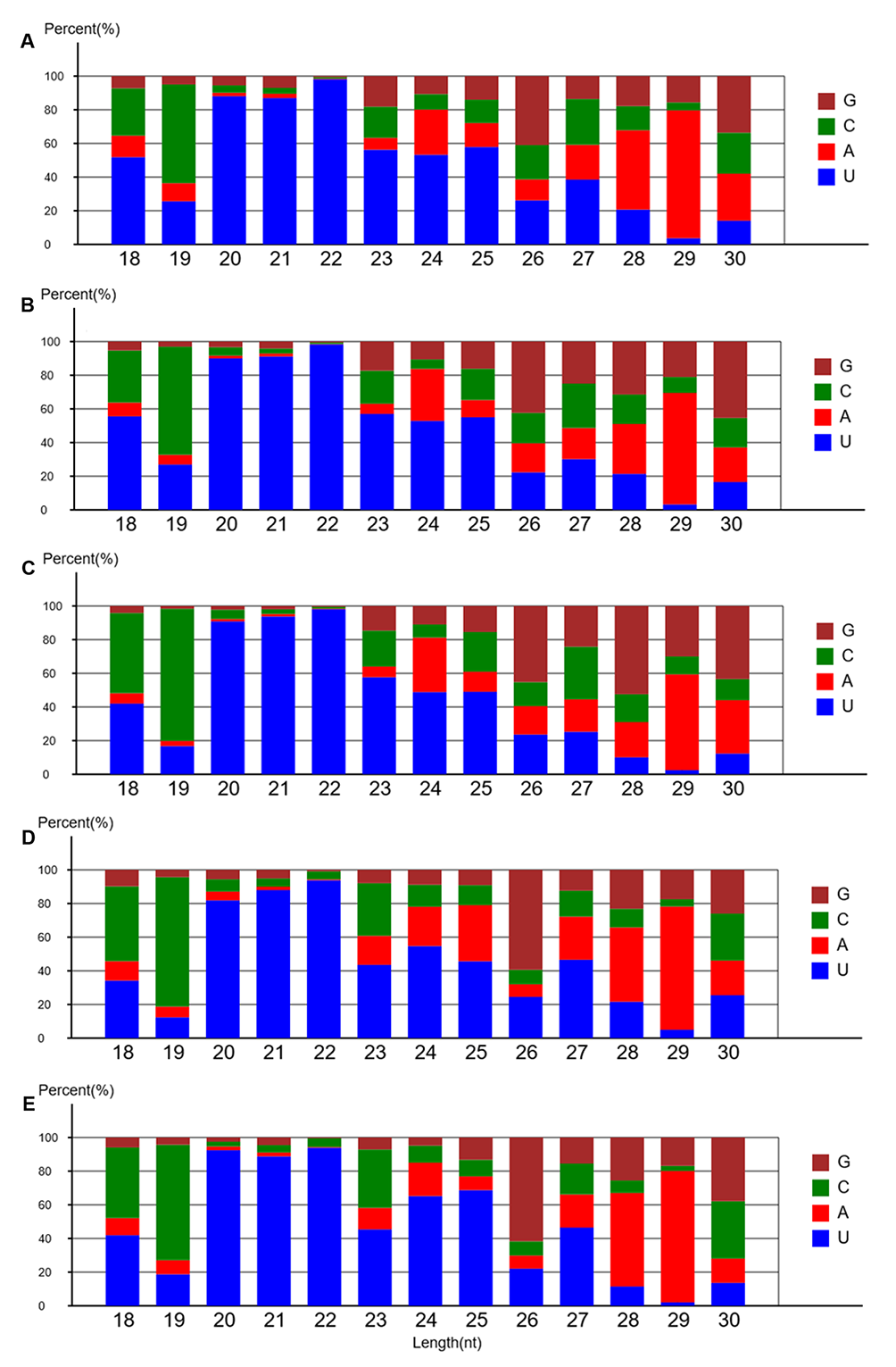

Supplement: Supplementary Figure 2 — The nucleotide bias of 18nt-30nt length known miRNAs at the 1st position in PYTZ. sBud (A), sL1 (B), sL2 (C), sS1 (D), and sS2 (E). [file Image_2.TIF]

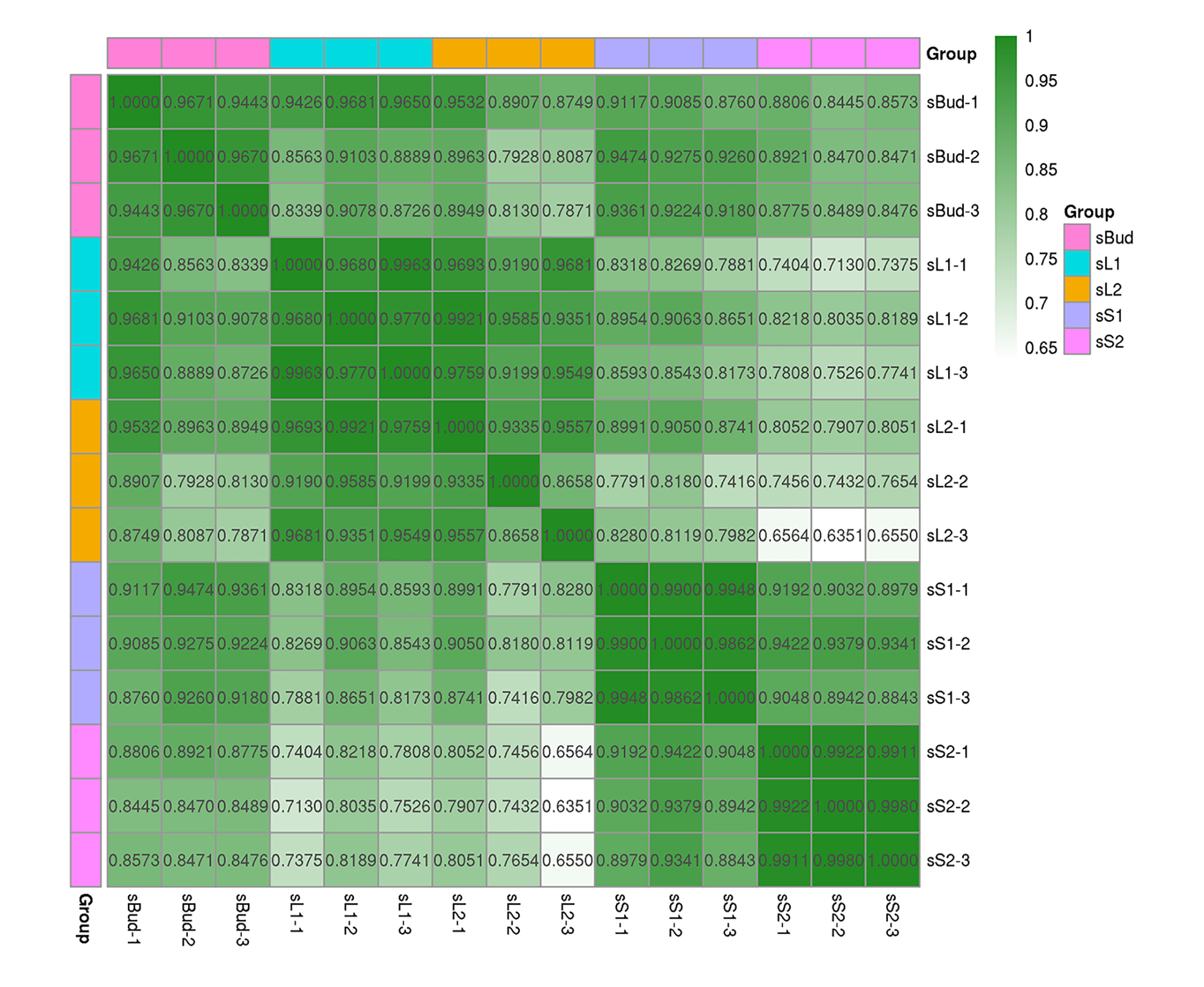

Supplement: Supplementary Figure 3 — Related coefficients of the 15 miRNA libraries across the five sample groups with three replicates of each group. [file Image_3.TIF]

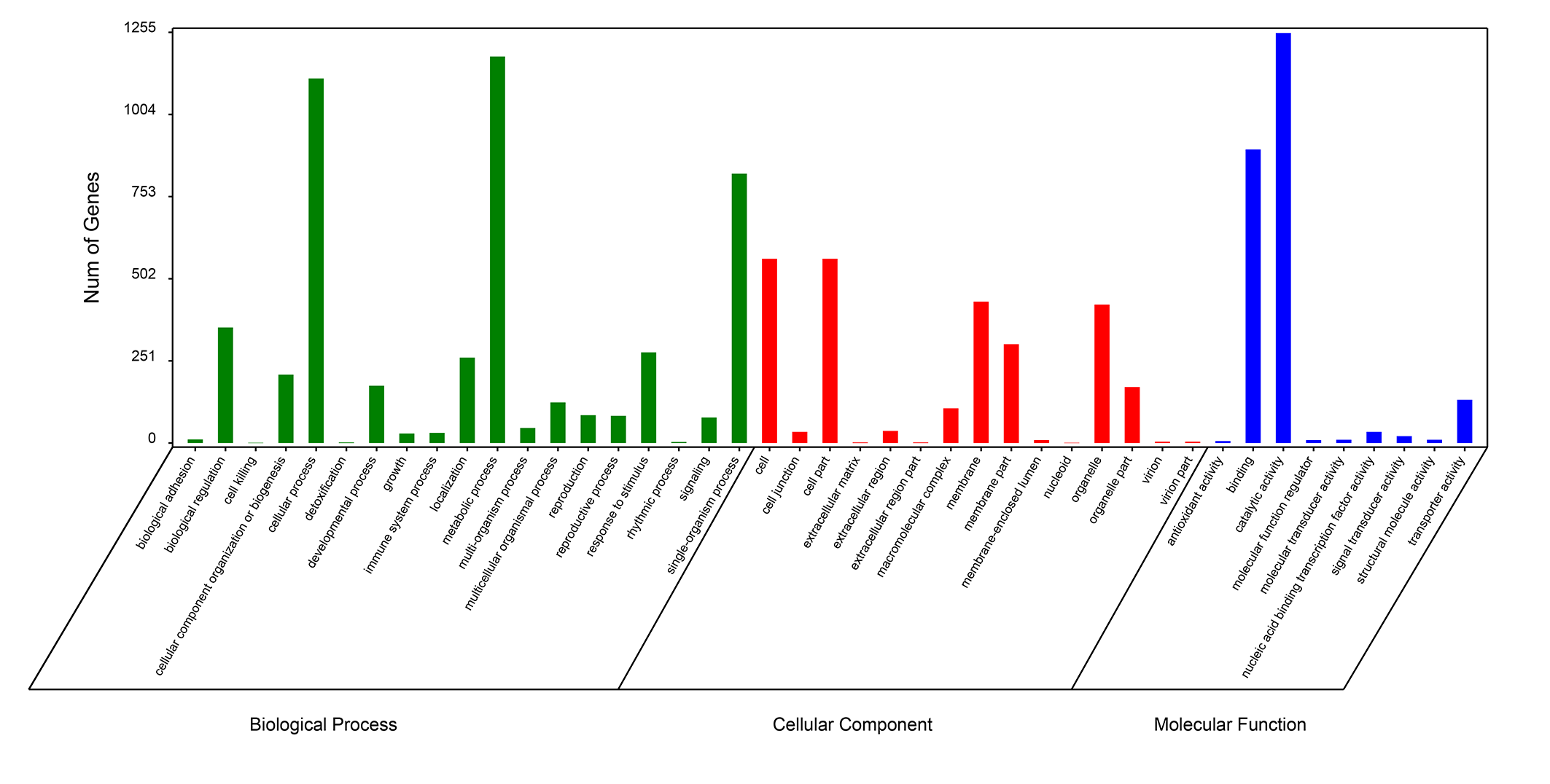

Supplement: Supplementary Figure 4 — Level 2 GO terms of all target genes from PTZY sprouts. [file Image_4.TIF]

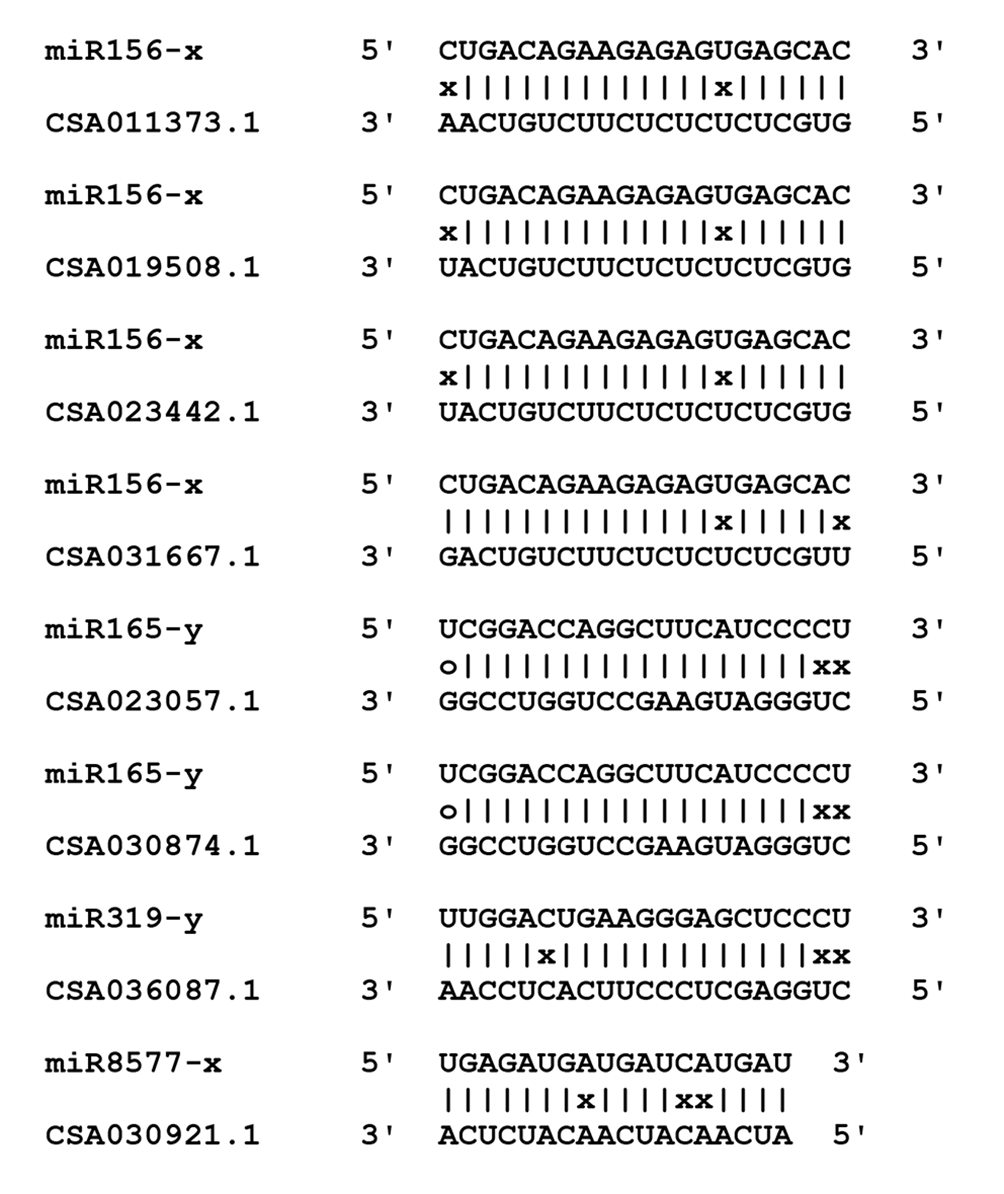

Supplement: Supplementary Figure 5 — Complementary correspondence of developmental miRNA toward the target sites. [file Image_5.TIF]

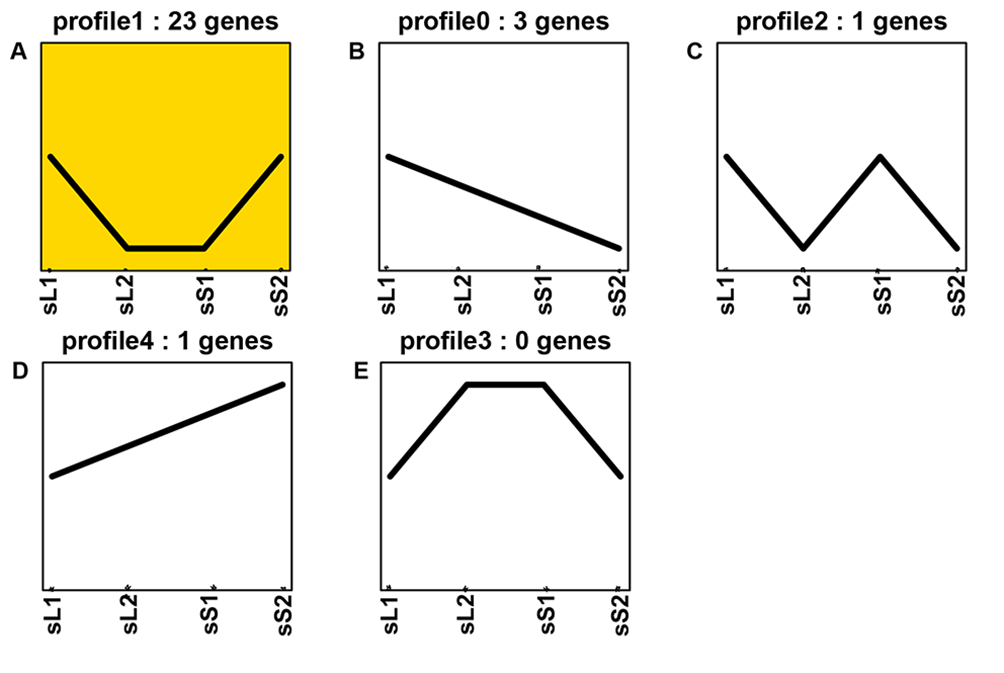

Supplement: Supplementary Figure 6 — (A–E) The expression profiles of miRNA that exist both in sL (leaf) and sS (stem). [file Image_6.TIF]

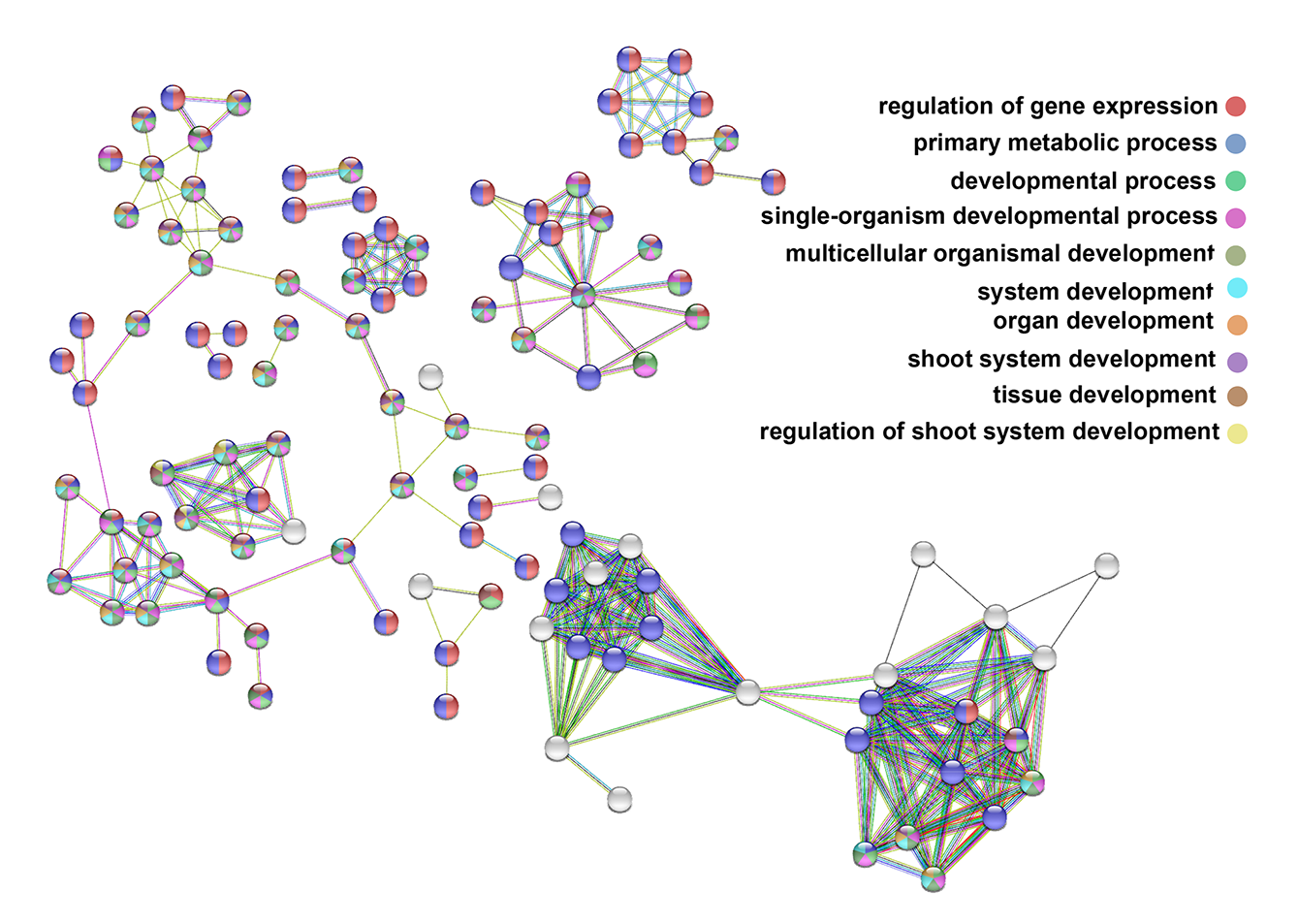

Supplement: Supplementary Figure 7 — Relative process of the targeted TF genes by STRING analysis. (STRING: https://string-db.org/). [file Image_7.TIF]

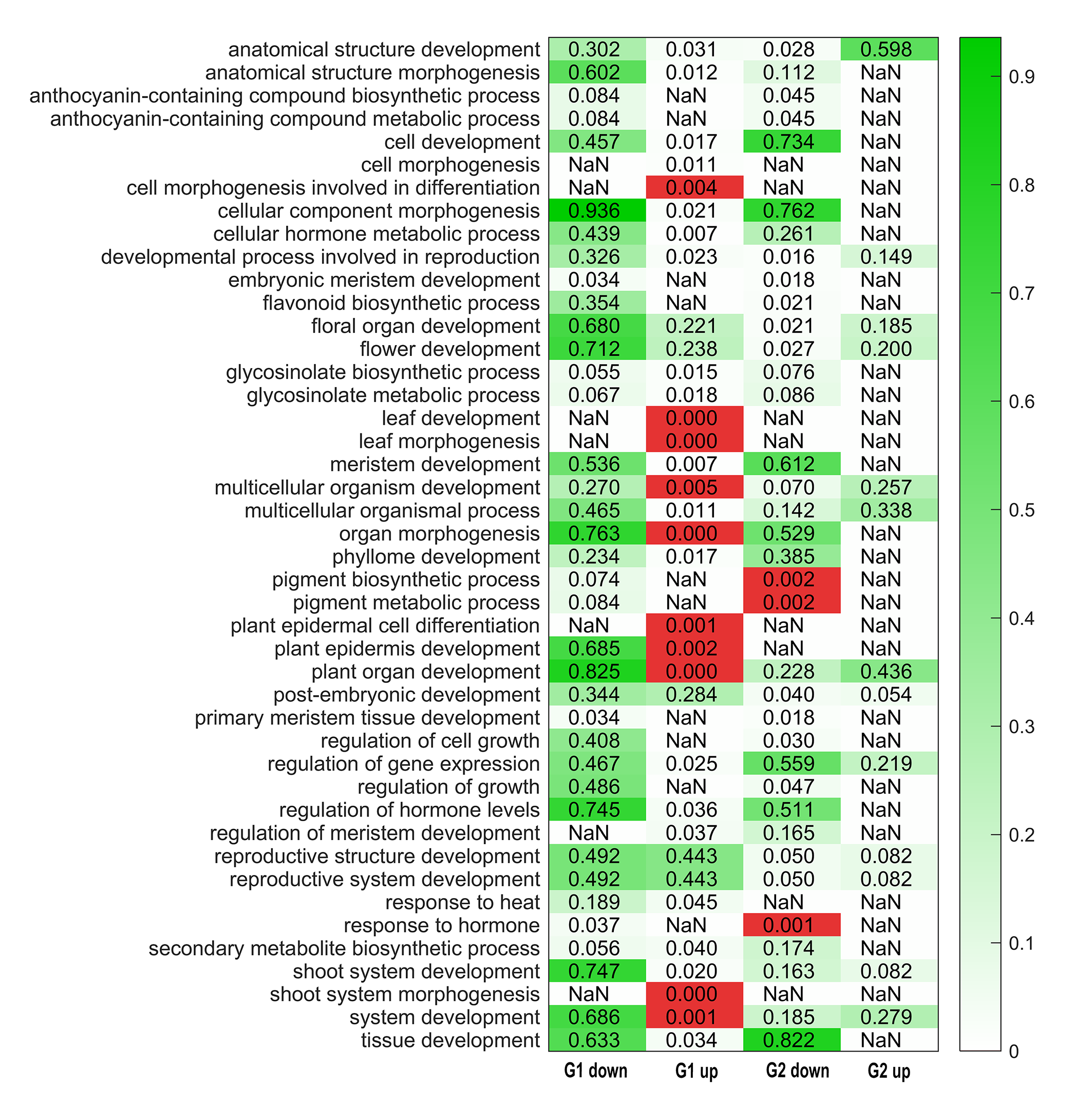

Supplement: Supplementary Figure 8 — Level 3 GO analysis of DEM in G1 and G2. [file Image_8.TIF]

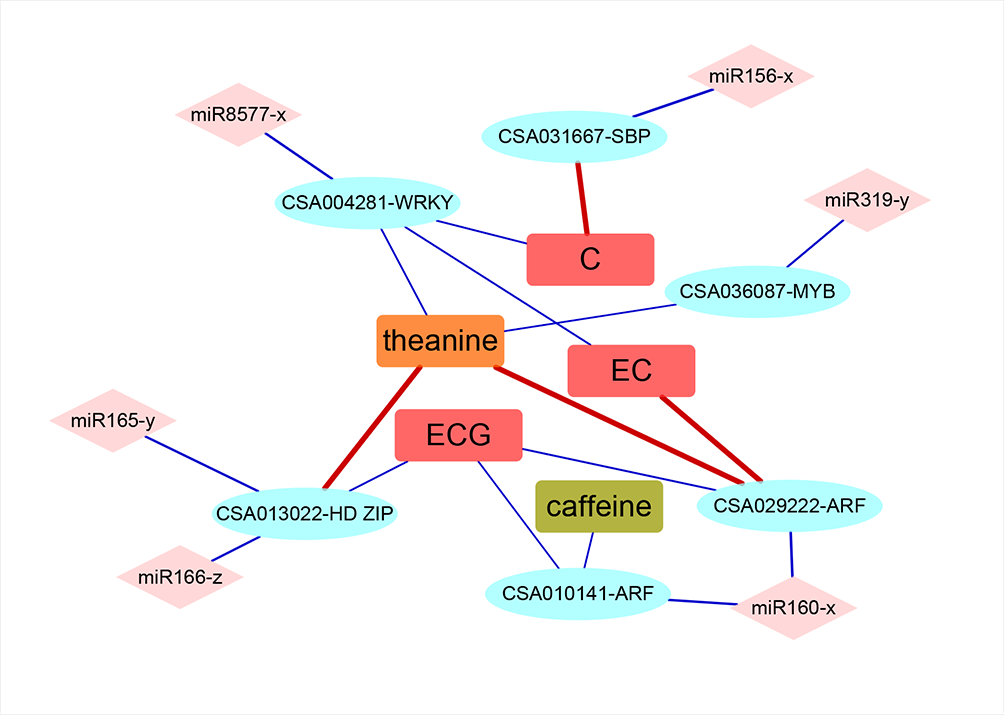

Supplement: Supplementary Figure 9 — The miRNA-TF-metabolites networks. The round rectangle placed in the center were metabolites, the ellipses at the interlayer were potential target mRNAs that belong to TF genes, and the diamond at the outermost layer were miRNAs. Line thickness represented the strength of the relationship. The red line represented the positive correlation efficient and the blue line meant negative. [file Image_9.TIF]
