# Supplementary material for: Conserved MicroRNA Act Boldly During Sprout Development and Quality Formation in Pingyang Tezaocha (Camellia sinensis)
Source: Front Genet. 2019 Mar 28;10:237. doi: 10.3389/fgene.2019.00237 (PMC6455055; doi:10.3389/fgene.2019.00237)
Supplement: Supplementary Table 1 — The primers sequences of miRNA and mRNA genes used in Real-time PCR. [file Table_1.DOCX]

Supplementary Table 1 The primers sequences of miRNA and mRNA genes used in Real-time PCR.

| miRNA / mRNA | Primers sequence (5’-3’) |
| --- | --- |
| miR156-x | CTGACAGAAGAGAGTGAGCAC |
| miR160-x | TGCCTGGCTCCCTGTATGCCA |
| miR164-x | TGGAGAAGCAGGGCACGTG |
| miR165-y | TCGGACCAGGCTTCATCCCCT |
| miR166-x | GGAATGTTGGCTGGCTCGAT |
| miR166-y | TCGGACCAGGCTTCATTCC |
| miR166-z | TCGGACCAGGCTTCATTCCCT |
| miR319-x | GAGCTTCCTTCTGTCCACTT |
| miR319-y | TTGGACTGAAGGGAGCTCCCT |
| miR390-x | AAGCTCAGGAGGGATAGCG |
| miR396-x | GTTCCACAGCTTTCTTGAACTT |
| miR396-y | GCTCAAGAAAGCTGTGGGAAG |
| miR5083-y | CGGCTACAATTATCTGATCAAA |
| miR8175-y | CCGGCAACGGCGCCAAA |
| miR8577-x | CGGTGAGATGATGATCATGAT |
| novel-m0675-3p | CGGGAATATGATGAATTTGAATG |
| novel-m0297-3p | CCTAACCCCAACACCCAATC |
| novel-m0243-3p | GATCAGGATGAAGCAACATT |
| novel-m0284-3p | TTGGGCTGGGCAGAAATTGG |
| novel-m0800-3p | GTTCAGTGAAGCTGTGGAAAG |
| novel-m0187-3p | CGGATTTCCCTTTCCAAATTCCTT |
| 5.8s | F: CTCGGCAACGGATATCTCG |
|  | R: CTAATGGCTTGGGGCG |
| CSA007311 | F: CCAAAATCCCTGTTCCCTC |
|  | R: ACAGCCTGACATCCCTATCC |
| CSA009012 | F: GCAGAAAATCGCACTCC |
|  | R: CCACCAACTTCGACCAT |
| CSA011373 | F: GGAGGTAATGAAAGCATCCAGG |
|  | R: CAATCAACGGTTCACCAGAG |
| CSA013149 | F: GTCCGAAGGAATCTATGCTC |
|  | R: GAATGGGGTGGTCAGGTA |
| CSA017838 | F: GCATCCAAAAGAACGACG |
|  | R: GGCAAATGGTGGTTTACCTG |
| CSA019508 | F: GAACAACCTCCCCGACCTCT |
|  | R: TTCTGGCTGTGGCTACTGG |
| CSA020439 | F: AGGTGCTGTTCCCACATG |
|  | R: TGCTTGGTTGCTGCTTC |
| CSA023442 | F: GATTTTCCCCTCACCGAC |
|  | R: GTGAACGCCACCCAACT |
| CSA031667 | F: TTTCTGCGAACTCCTCTACC |
|  | R: ACCTTGGGCGTTTCCTT |
| CSA021765 | F: TCCTATGAGAAGCAACGAGC |
|  | R: TGGCGAAAGAAGCAGGT |
| CSA026035 | F: CACCCGATAGTCAGAGCA |
|  | R: GAACGAACCCAAACCAG |
| CSA026847 | F: GGCAGTTCACAGACCGA |
|  | R: CAAAGCCATTCTCCACAG |
| CSA013362 | F: TGCCTTCCAATAACCAGC |
|  | R: GATTCCACCCAACCGAC |
| CSA027185 | F: GTGAACGGATTCGGACG |
|  | R: GTGGGTGATTTGTAGGGTGT |
| CSA033493 | F: CTGGGTAGAGGTTGATGCC |
|  | R: CGGGTGTTCCAAAAGAGTG |
| CSA023057 | F: TCTGCCCTTGAGGTTGG |
|  | R: TCGCACATACAGACGAGCC |
| CSA030874 | F: AGCAAGTGGGGAGGTAGT |
|  | R: TAGGATGCCGCCAAGTA |
| CSA001544 | F: GTTGGGGCATTGGCTGTA |
|  | R: ATGTTGGCTGGCTCGGT |
| CSA017082 | F: GCCCAAGAACTTATCCG |
|  | R: ATCAGGTGCCTTTACCG |
| CSA017802 | F: CACGAGGACCAAGACGA |
|  | R: TGCCGCTGACTGTGAAT |
| CSA023955 | F: CTGGACGGCTTGAACAC |
|  | R: GCTTCGGCTTTTACCTG |
| CSA028252 | F: TCGCCACCTTCTATGACT |
|  | R: AGGGACAATCGGCTATCT |
| CSA028940 | F: GGTTCCCCTGGGTTTCT |
|  | R: CGTTCTGCCATGACTCGA |
| CSA031362 | F: CTTCCGCCCAAAAGTAG |
|  | R: ATAGCCCATTGTCCCTCT |
| CSA031363 | F: CACGAGGACCAAGACGA |
|  | R: GCCGCTGACTGTGAATC |
| CSA030874 | F: TCGTCCATCTTGGTTCC |
|  | R: AGCATTTCAGCCCTCAC |
| CSA003974 | F: ATGCCCAAAACCTCGTG |
|  | R: ATCCCTGAAGGCTGCTC |
| CSA005021 | F: CACCGCTGAGATGGACA |
|  | R: TGTTTGGGGCACGAAGG |
| CSA007451 | F: AATGGGCTCTGGTGGTG |
|  | R: CTATGCCGCTGCTTTCA |
| CSA014848 | F: GGAGGTGGTTCAGGTTTG |
|  | R: TGCTGGGACTTACGATGT |
| CSA017867 | F: ATTTCTTCCCCATCTCGC |
|  | R: GGATTCTTGCTGACCTCGTA |
| CSA018024 | F: TACTGGAAGGCGTAGCAT |
|  | R: GTAACCTCGCTGACCGT |
| CSA018464 | F: AGTGCCAAACGACGCTC |
|  | R: CACTGCCAGGAAAACCC |
| CSA021711 | F: GGAGGGAAGCACCTATCA |
|  | R: GGGTTTGTCGCCAGTTT |
| CSA036427 | F: CCATTTGGTGCCGTCTT |
|  | R: GTTGGGTGATCTTGGTCAGT |
| CSA002826 | F: GGAGTTGCTCACTACAGCC |
|  | R: GTAATCTCGTAGAAGCCCAC |
| CSA013833 | F: CACCAGCCACAACAGAAA |
|  | R: TGAGCAAGCACAGTCGG |
| CSA031080 | F: ATGAAGCTGGGTGGAGTG |
|  | R: TGAGTGGACCGAAGGAAG |
| CSA036087 | F: TGGGGTGAATGCTCCTT |
|  | R: GGTTGTTCCGTTGTGGG |
| CSA001776 | F: ACTTGGCATGTACTCGGTC |
|  | R: CCAGAAAGCATTGGGCTA |
| CSA001956 | F: GCATTGTTGGTCGGGATT |
|  | R: TTGCGGTTGCCATCGTC |
| CSA006028 | F: TCAGGAGGCATAGTGGC |
|  | R: AGGAGTTGGGGAGCATT |
| CSA007412 | F: GAGGACGAAGTCACGGGTTT |
|  | R: GTGGTAGCAGGAATGATACTAGAGG |
| CSA011969 | F: GGCGATGGCGAGTCTTGTT |
|  | R: GCAGTTATGCCTTCTGGTATGG |
| CSA016385 | F: TCTCAGTCTTACAAGGAGGC |
|  | R: GACCGAATAGCAGCGAC |
| CSA022179 | F: CTGGAGGAAGGTCAGTTAGT |
|  | R: GCACAGGTCGAGCGTTT |
| CSA026282 | F: TAGGAAAGTGGCATCGC |
|  | R: AACCTTCTCGGAAACCC |
| CSA030630 | F: TATGAACAGAGGACCAACGG |
|  | R: CCAACGACATCGCTTACAAC |
| CSA036196 | F: GTGCTGTGGATGCGAACA |
|  | R: CATTGGCGTGGGAGGAT |
| CSA003330 | F: GTGCTTTTAGGGTGGGA |
|  | R: TTTGTGCTGGAGACGGT |
| CSA036373 | F: ATGCTTCTCGTCGTTTTCGG |
|  | R: CCTGCTACCCCTTTTACCCTTA |
| CSA013921 | F: GTTGGATTCAGTGGTAGGG |
|  | R: CCTCGGTGTAGTAATCGTCA |
| CSA018936 | F: CGCTATGACCTCGCCACTT |
|  | R: TCGGGATCGGCCTATTT |
| CSA008171 | F: TTCATCAAACCCGAGCAG |
|  | R: CCGCCTTTTGCCACTAA |
| CSA030921 | F: GTTCATACGTCCAAAGGG |
|  | R: TTCCACATCTGCCAAGC |
| CSA006098 | F: AAGAGGTCGGAGGAGACA |
|  | R: AGGCAGTGAAGCCAGTTA |
| CSA012411 | F: TGTGAACTCGGAGGGTAG |
|  | R: ATCAGCATACTTGGACGG |
| CSA027591 | F: ATTTCCCGCCATAGTTCG |
|  | R: CCTCCAGCGTCTTTCACA |
| CSA034664 | F: ATGGATGCTGTGGAACG |
|  | R: CATGGGAAGGTCACGAAC |
| CSA016454 | F: AGAAAATGGCACCAGTCC |
|  | R: TGCGGATACCTGTGAGAA |
| CSA010612 | F: GGATGGTGAACGATGAGG |
|  | R: AGTACAGCAGCCCGTAGTC |
| CSA028516 | F: AACTGGGTGCCGTTGTC |
|  | R: ATCGCAGCCACAGATTAC |
| CSA012066 | F: CCCACCTTTTCCATTTCC |
|  | R: GAAGCCATTGCGTTTGAG |
| CSA024775 | F: AAAGGGGAGTTGCTCTGA |
|  | R: GCATCTCGGCAGTTAGTT |
| CSA011373 | F: CCAGGAATGCTTTTGAGG |
|  | R:CAGGAATCGCGGAGAAC |
| CSA020439 | F: GGGCGAGGCTTTTCAGT |
|  | R: TGCTCTTTGCCACCGAC |
| CSA023442 | F: ATGTGAACGCCACCCAA |
|  | R:AGCCCAAATGCCGAACT |
| CSA031667 | F: TTTCTGCGAACTCCTCTACC |
|  | R: ACCTTGGGCGTTTCCTT |
| GAPDH | F: TTGGCATCGTTGAGGGTCT |
|  | R: CAGTGGGAACACGGAAAGC |
