# Supplementary material for: Conserved MicroRNA Act Boldly During Sprout Development and Quality Formation in Pingyang Tezaocha (Camellia sinensis)
Source: Front Genet. 2019 Mar 28;10:237. doi: 10.3389/fgene.2019.00237 (PMC6455055; doi:10.3389/fgene.2019.00237)
Supplement: Supplementary Table 2 — The filtering data of 15 sRNA-Seq libraries from Camellia sinensis cv. Pingyang Tezaocha. [file Table_2.DOCX]

|  | raw_reads | high_quality^*^ | 3'adapter_null | insert_null | 5'adapter_contaminants | smaller_than_18nt | polyA | low cutoff | clean_tags |
| --- | --- | --- | --- | --- | --- | --- | --- | --- | --- |
| sBud-1 | 21881850 (100%) | 21567729 (98.5645%) | 613922 (2.8465%) | 1240290 (5.7507%) | 219247 (1.0166%) | 3018141 (13.9938%) | 2341 (0.0109%) | 3448292 (15.9882%) | 13025496 (60.3935%) |
| sBud-2 | 21459760 (100%) | 21141900 (98.5188%) | 479217 (2.2667%) | 688562 (3.2569%) | 206385 (0.9762%) | 3022844 (14.2979%) | 1617 (0.0076%) | 3071133 (14.5263%) | 13672142 (64.6685%) |
| sBud-3 | 22627128 (100%) | 22299013 (98.5499%) | 272005 (1.2198%) | 326849 (1.4658%) | 160513 (0.7198%) | 3108827 (13.9415%) | 1547 (0.0069%) | 2885353 (12.9394%) | 15543919 (69.7068%) |
| sL1-1 | 21558682 (100%) | 21249734 (98.5669%) | 559663 (2.6337%) | 737494 (3.4706%) | 108527 (0.5107%) | 2244181 (10.5610%) | 1880 (0.0088%) | 4380596 (20.6148%) | 13217393 (62.2003%) |
| sL1-2 | 21800219 (100%) | 21483668 (98.5479%) | 370333 (1.7238%) | 778999 (3.6260%) | 234098 (1.0897%) | 4132469 (19.2354%) | 1196 (0.0056%) | 3880297 (18.0616%) | 12086276 (56.2580%) |
| sL1-3 | 20044344 (100%) | 19751046 (98.5368%) | 830019 (4.2024%) | 604489 (3.0605%) | 94180 (0.4768%) | 1870114 (9.4684%) | 2080 (0.0105%) | 3323617 (16.8275%) | 13026547 (65.9537%) |
| sL2-1 | 19736769 (100%) | 19447416 (98.5339%) | 426595 (2.1936%) | 1370069 (7.0450%) | 216916 (1.1154%) | 3319291 (17.0680%) | 1148 (0.0059%) | 2966809 (15.2555%) | 11146588 (57.3166%) |
| sL2-2 | 21444252 (100%) | 21143803 (98.5989%) | 1441323 (6.8168%) | 174892 (0.8272%) | 57457 (0.2717%) | 1539293 (7.2801%) | 1319 (0.0062%) | 4372883 (20.6816%) | 13556636 (64.1164%) |
| sL2-3 | 18679971 (100%) | 18396220 (98.4810%) | 560054 (3.0444%) | 676052 (3.6750%) | 196612 (1.0688%) | 3472220 (18.8746%) | 1147 (0.0062%) | 2701489 (14.6850%) | 10788646 (58.6460%) |
| sS1-1 | 17061217 (100%) | 16598519 (97.2880%) | 585916 (3.5299%) | 375553 (2.2626%) | 171277 (1.0319%) | 2577493 (15.5285%) | 1566 (0.0094%) | 2065473 (12.4437%) | 10821241 (65.1940%) |
| sS1-2 | 17125467 (100%) | 16642947 (97.1824%) | 628419 (3.7759%) | 393581 (2.3649%) | 156337 (0.9394%) | 2262795 (13.5961%) | 1643 (0.0099%) | 2175007 (13.0686%) | 11025165 (66.2453%) |
| sS1-3 | 19894021 (100%) | 19609707 (98.5709%) | 338690 (1.7272%) | 441072 (2.2493%) | 273634 (1.3954%) | 3838030 (19.5721%) | 1509 (0.0077%) | 2110155 (10.7608%) | 12606617 (64.2876%) |
| sS2-1 | 14827106 (100%) | 14605676 (98.5066%) | 318535 (2.1809%) | 362264 (2.4803%) | 145947 (0.9992%) | 2406212 (16.4745%) | 1462 (0.0100%) | 2245791 (15.3762%) | 9125465 (62.4789%) |
| sS2-2 | 19309590 (100%) | 19027956 (98.5415%) | 417774 (2.1956%) | 755464 (3.9703%) | 252378 (1.3264%) | 3675595 (19.3168%) | 1913 (0.0101%) | 2621498 (13.7771%) | 11303334 (59.4038%) |
| sS2-3 | 15202984 (100%) | 14956393 (98.3780%) | 615037 (4.1122%) | 420861 (2.8139%) | 156297 (1.0450%) | 2322074 (15.5256%) | 1896 (0.0127%) | 2400281 (16.0485%) | 9039947 (60.4420%) |

Supplementary Table 2 The filtering data of 15 sRNA-Seq libraries from *Camellia sinensis* cv. Pingyang Tezaocha.

*: reads that after filter out low quality reads which have quality values less than 20 or the reads contain N.
