# Supplementary material for: Conserved MicroRNA Act Boldly During Sprout Development and Quality Formation in Pingyang Tezaocha (Camellia sinensis)
Source: Front Genet. 2019 Mar 28;10:237. doi: 10.3389/fgene.2019.00237 (PMC6455055; doi:10.3389/fgene.2019.00237)
Supplement: Supplementary Table 3 — Statistics of sRNA-Seq libraries mapping to tea tree genome. [file Table_3.DOCX]

| sample | total | mapping | exon_sense | exon_antisense | intron_sense | intron_antisense |
| --- | --- | --- | --- | --- | --- | --- |
| sBud-1 | 13025496 | 10479927(80.46%) | 1767812 (13.57%) | 3739707 (28.71%) | 1023712 (7.86%) | 1775869 (13.63%) |
| sBud-2 | 13672142 | 10645544(18.18%) | 1961266 (14.34%) | 3167294 (23.17%) | 1007749 (7.37%) | 1368657 (10.01%) |
| sBud-3 | 15543919 | 12500708(80.42%) | 2398183 (15.43%) | 3450380 (22.20%) | 1131398 (7.28%) | 1306308 (8.40%) |
| sL1-1 | 13217393 | 10505086(79.48%) | 1715029 (12.98%) | 3998301 (30.25%) | 1128540 (8.54%) | 2362266 (17.87%) |
| sL1-2 | 12086276 | 9609658(79.51%) | 1699029 (14.06%) | 3096087 (25.62%) | 1086692 (8.99%) | 1526271 (12.63%) |
| sL1-3 | 13026547 | 10493892(80.56%) | 1990807 (15.28%) | 3870121 (29.71%) | 1012527 (7.77%) | 1960781 (15.05%) |
| sL2-1 | 11146588 | 8791463(78.87%) | 1583076 (14.20%) | 2936923 (26.35%) | 933666 (8.38%) | 1404214 (12.60%) |
| sL2-2 | 13556636 | 10502614(77.47%) | 2347839 (17.32%) | 3310475 (24.42%) | 1456019 (10.74%) | 1926125 (14.21%) |
| sL2-3 | 10788646 | 7992319(74.08%) | 1203208 (11.15%) | 3571843 (33.11%) | 937677 (8.69%) | 2182428 (20.23%) |
| sS1-1 | 10821241 | 7632765(70.54%) | 1238542 (11.45%) | 2554043 (23.60%) | 756139 (6.99%) | 1134650 (10.49%) |
| sS1-2 | 11025165 | 7658889(69.47%) | 1283536 (11.64%) | 2565890 (23.27%) | 826992 (7.50%) | 1175077 (10.66%) |
| sS1-3 | 12606617 | 9533436(75.62%) | 1513238 (12.00%) | 3279953 (26.02%) | 1072498 (8.51%) | 1591175 (12.62%) |
| sS2-1 | 9125465 | 6997001(76.68%) | 1117845 (12.25%) | 2463142 (26.99%) | 706814 (7.75%) | 1214889 (13.31%) |
| sS2-2 | 11303334 | 8806414(77.91%) | 1560747 (13.81%) | 2490571 (22.03%) | 963082 (8.52%) | 1185010 (10.48%) |
| sS2-3 | 9039947 | 6837820(75.64%) | 1188676 (13.15%) | 2004064 (22.17%) | 792714 (8.77%) | 1011945 (11.19%) |

Supplementary Table 3 Statistics of sRNA-Seq libraries mapping to tea tree genome.
