# Supplementary material for: Conserved MicroRNA Act Boldly During Sprout Development and Quality Formation in Pingyang Tezaocha (Camellia sinensis)
Source: Front Genet. 2019 Mar 28;10:237. doi: 10.3389/fgene.2019.00237 (PMC6455055; doi:10.3389/fgene.2019.00237)
Supplement: Supplementary Table 4 — Statistics of sRNA-Seq libraries mapping to Rfam. [file Table_4.DOCX]

| sample | total | rRNA | snRNA | snoRNA | tRNA |
| --- | --- | --- | --- | --- | --- |
| sBud-1 | 13025496 | 2497232(19.17%) | 23537(0.18%) | 91027(0.70%) | 107946(0.83%) |
| sBud-2 | 13672142 | 2485757(18.18%) | 23745(0.17%) | 94896(0.69%) | 80556(0.59%) |
| sBud-3 | 15543919 | 2772675(17.84%) | 25698(0.17%) | 82283(0.53%) | 58085(0.37%) |
| sL1-1 | 13217393 | 2058045(15.57%) | 19443(0.15%) | 54156(0.41%) | 295330(2.23%) |
| sL1-2 | 12086276 | 1890750(15.64%) | 23543(0.19%) | 66380(0.55%) | 99903(0.83%) |
| sL1-3 | 13026547 | 2433074(18.68%) | 17522(0.13%) | 49649(0.38%) | 202101(1.55%) |
| sL2-1 | 11146588 | 1847586(16.58%) | 16397(0.15%) | 39833(0.36%) | 123006(1.10%) |
| sL2-2 | 13556636 | 2418163(17.84%) | 26346(0.19%) | 18368(0.14%) | 305336(2.25%) |
| sL2-3 | 10788646 | 1765804(16.37%) | 18992(0.18%) | 65507(0.61%) | 182308(1.69%) |
| sS1-1 | 10821241 | 2086044(19.28%) | 18953(0.18%) | 72354(0.67%) | 41719(0.39%) |
| sS1-2 | 11025165 | 2141647(19.43%) | 21306(0.19%) | 70072(0.64%) | 42271(0.38%) |
| sS1-3 | 12606617 | 2496268(19.80%) | 28550(0.23%) | 125159(0.99%) | 60012(0.48%) |
| sS2-1 | 9125465 | 1788673(19.60%) | 17654(0.19%) | 64025(0.70%) | 82252(0.90%) |
| sS2-2 | 11303334 | 2076419(18.37%) | 26011(0.23%) | 64039(0.57%) | 72964(0.65%) |
| sS2-3 | 9039947 | 1748250(19.34%) | 28990(0.32%) | 47295(0.52%) | 79260(0.88%) |

Supplementary Table 4 Statistics of sRNA-Seq libraries mapping to Rfam.
