# Supplementary material for: Conserved MicroRNA Act Boldly During Sprout Development and Quality Formation in Pingyang Tezaocha (Camellia sinensis)
Source: Front Genet. 2019 Mar 28;10:237. doi: 10.3389/fgene.2019.00237 (PMC6455055; doi:10.3389/fgene.2019.00237)
Supplement: Supplementary Table 5 — The lengths, sequences, and expressions of 156 known miRNAs. [file Table_5.DOCX]

Supplementary Table 5 The lengths, sequences, and expressions of 156 known miRNAs.

| ID | length | sequence | total | Count average | | | | |
| --- | --- | --- | --- | --- | --- | --- | --- | --- |
|  |  |  |  | sBud | sL1 | sL2 | sS1 | sS2 |
| miR166-y | 21 | TCGGACCAGGCTTCATTCCCC | 604867 | 28359 | 75034 | 67029 | 11922 | 19278 |
| miR159-y | 21 | TTTGGATTGAAGGGAGCTCTA | 282768 | 14343 | 27905 | 27093 | 5869 | 19046 |
| miR482-y | 22 | TTCCCAATCCCTCCCATTCCTA | 212020 | 12585 | 20524 | 14173 | 4854 | 18538 |
| miR472-y | 22 | TTTCCGACTCCGCCCATGCCAT | 184817 | 18077 | 13522 | 7232 | 7936 | 14838 |
| miR7767-x | 19 | CCAAGATGAGTGCTCTCCC | 110018 | 4113 | 6919 | 13203 | 6640 | 5798 |
| miR166-z | 21 | TCGGACCAGGCTTCATTCCCT | 93690 | 4608 | 8713 | 11576 | 2756 | 3577 |
| miR2118-x | 22 | TTCCCAATCCCTCCCATTCCTA | 90863 | 5490 | 7918 | 5020 | 2449 | 9410 |
| miR319-y | 21 | TTGGACTGAAGGGAGCTCCCT | 73506 | 1459 | 453 | 309 | 5413 | 16868 |
| miR167-x | 22 | TGAAGCTGCCAGCATGATCTAG | 36199 | 1186 | 5648 | 5086 | 15 | 132 |
| miR894-x | 18 | TTCACGTCGGGTTCACCA | 33255 | 2733 | 3176 | 2806 | 964 | 1406 |
| miR166-x | 21 | GGAATGTTGGCTGGCTCGATG | 29446 | 2973 | 3752 | 1472 | 580 | 1039 |
| miR396-x | 21 | TTCCACAGCTTTCTTGAACTT | 18253 | 90 | 1287 | 3863 | 135 | 709 |
| miR168-x | 21 | TCGCTTGGTGCAGGTCGGGAA | 15863 | 1103 | 2007 | 954 | 418 | 805 |
| miR8558-y | 20 | TTTCCGACTCCGCCCATGCC | 11850 | 1124 | 790 | 448 | 562 | 1026 |
| miR160-x | 21 | TGCCTGGCTCCCTGTATGCCA | 10989 | 1706 | 962 | 305 | 249 | 441 |
| miR168-y | 21 | CCCGCCTTGCATCAACTGAAT | 10550 | 642 | 1113 | 515 | 359 | 887 |
| miR162-y | 21 | TCGATAAACCTCTGCATCCAG | 10262 | 431 | 1110 | 974 | 205 | 701 |
| miR394-x | 20 | TTGGCATTCTGTCCACCTCC | 9108 | 280 | 1072 | 1028 | 154 | 502 |
| miR384-x | 20 | TTGGCATTCTGTCCACCTCC | 8836 | 262 | 1045 | 1006 | 149 | 483 |
| miR858-x | 21 | TTCGTTGTCTGTTCGACCTTG | 8778 | 302 | 819 | 973 | 217 | 616 |
| miR2118-y | 24 | TCTTCCCAATCCCTCCCATTCCTA | 7333 | 525 | 654 | 349 | 250 | 666 |
| miR3630-y | 22 | TTTGGGAATCTCTTTGATGCAC | 6027 | 471 | 409 | 269 | 573 | 286 |
| miR5072-x | 18 | TCCCCAGCAGAGTCGCCA | 4139 | 387 | 395 | 268 | 78 | 251 |
| miR390-x | 21 | AAGCTCAGGAGGGATAGCGCC | 3947 | 552 | 345 | 95 | 65 | 258 |
| miR396-y | 21 | GCTCAAGAAAGCTGTGGGAAG | 3920 | 97 | 456 | 257 | 63 | 433 |
| miR164-x | 21 | TGGAGAAGCAGGGCACGTGCA | 3907 | 120 | 487 | 332 | 59 | 305 |
| miR171-x | 21 | TATTGGCCTGGTTCACTCAGA | 3313 | 168 | 327 | 240 | 81 | 289 |
| miR395-y | 21 | CTGAAGTGTTTGGGGGAACTC | 3247 | 49 | 509 | 446 | 21 | 57 |
| miR165-y | 21 | TCGGACCAGGCTTCATCCCCT | 3235 | 132 | 280 | 447 | 88 | 131 |
| miR403-y | 21 | TTAGATTCACGCACAAACTCG | 2146 | 108 | 165 | 185 | 75 | 182 |
| miR398-y | 18 | TGTGTTCTCAGGTCGCCC | 1966 | 154 | 221 | 105 | 63 | 112 |
| miR170-x | 21 | TATTGGCCTGGTTCACTCAGA | 1916 | 151 | 254 | 166 | 22 | 46 |
| miR8175-y | 18 | TCCCCGGCAACGGCGCCA | 1900 | 188 | 175 | 111 | 37 | 122 |
| miR171-y | 21 | TGATTGAGCCGTGCCAATATC | 1817 | 116 | 260 | 186 | 8 | 36 |
| miR6478-x | 20 | CCGACCTTAGCTCAGTTGGC | 1723 | 79 | 148 | 213 | 34 | 101 |
| miR171-z | 21 | TTGAGCCGCGCCAATATCACT | 1707 | 91 | 134 | 54 | 81 | 209 |
| miR7122-x | 22 | TTAAACAGAGAAATCACGGTGG | 1485 | 76 | 138 | 146 | 12 | 124 |
| miR479-x | 22 | CGTGATGTTGGTCCGGCTCATC | 1067 | 2 | 32 | 38 | 58 | 226 |
| miR7972-y | 21 | TTGTCAGGGTTGTGATTCTCC | 963 | 37 | 124 | 117 | 10 | 33 |
| miR8577-x | 18 | TGAGATGATGATCATGAT | 838 | 66 | 37 | 36 | 69 | 71 |
| miR167-y | 23 | GGTCATGCTCTGACAGCCTCACT | 811 | 41 | 98 | 92 | 13 | 27 |
| miR2916-x | 18 | CTCGAAGACGATCAGATG | 777 | 40 | 21 | 32 | 131 | 36 |
| miR535-x | 21 | TGACAACGAGAGAGAGCACGC | 725 | 16 | 75 | 108 | 4 | 39 |
| miR5059-x | 18 | TCCTGGGCAGCATCACCA | 677 | 57 | 73 | 43 | 11 | 41 |
| miR3633-y | 21 | TTCCTAAACCACCCATTCCTT | 633 | 26 | 57 | 52 | 14 | 63 |
| miR156-x | 21 | CTGACAGAAGAGAGTGAGCAC | 564 | 15 | 47 | 76 | 6 | 44 |
| miR319-x | 20 | GAGCTTCCTTCTGTCCACTT | 475 | 38 | 11 | 3 | 48 | 59 |
| miR172-y | 21 | AGAATCTTGATGATGCTGCAT | 469 | 9 | 47 | 88 | 3 | 9 |
| miR5083-y | 19 | CTACAATTATCTGATCAAA | 413 | 52 | 23 | 10 | 33 | 20 |
| miR6118-y | 21 | TTTCCAAGTCCACCCATTCCA | 375 | 22 | 32 | 43 | 2 | 26 |
| miR160-y | 21 | GCGTATGAGGAGCCATGCATA | 364 | 73 | 19 | 4 | 7 | 19 |
| miR5168-y | 21 | TCGGACCAGGCTTCACTCCCT | 359 | 17 | 40 | 45 | 9 | 9 |
| miR8016-y | 24 | ATTTTTGAATGAAAGGCCCATGTG | 307 | 11 | 43 | 22 | 4 | 23 |
| miR6483-y | 20 | TATTGTAGAAATTTTCGGGA | 281 | 17 | 23 | 25 | 16 | 12 |
| miR1863-y | 21 | AATGCTCTGATACCATGTTAA | 276 | 24 | 31 | 19 | 9 | 9 |
| miR530-x | 21 | TGCATTTACACCTGCACCTTG | 245 | 10 | 27 | 35 | 0 | 10 |
| miR482-x | 20 | GGAATGGGTCGATTGGGAAG | 213 | 18 | 20 | 11 | 5 | 16 |
| miR1511-y | 18 | AACCTGGCTCTGATACCA | 208 | 16 | 21 | 18 | 5 | 9 |
| miR5077-x | 21 | TTCTTCACGTCGGGTTCACCA | 189 | 12 | 18 | 15 | 11 | 8 |
| miR398-x | 18 | GGGGACGGACTGGGAACA | 160 | 18 | 10 | 12 | 7 | 6 |
| miR2218-y | 22 | TTTCCGAGTCCACCCATTCCTA | 146 | 10 | 17 | 10 | 0 | 11 |
| miR5139-x | 18 | AACCTGGCTCTGATACCA | 144 | 13 | 15 | 14 | 1 | 5 |
| miR8155-y | 18 | AACCTGGCTCTGATACCA | 141 | 12 | 15 | 14 | 1 | 5 |
| miR408-y | 21 | TGCACTGCCTCTTCCCTGGCT | 129 | 5 | 13 | 18 | 1 | 6 |
| miR6149-x | 18 | ATACGCACCTGAATCGGT | 126 | 5 | 14 | 17 | 2 | 5 |
| miR8022-x | 18 | TTCAAATGAGAACTTTGG | 110 | 7 | 6 | 7 | 8 | 8 |
| miR169-x | 22 | TAGCCAAGGACGACTTGCCTCG | 94 | 4 | 11 | 5 | 3 | 8 |
| miR4414-x | 21 | TGCTGCTGACTCGATGGTTCA | 94 | 16 | 0 | 2 | 5 | 8 |
| miR399-y | 21 | TGCCAAAGGAGAGTTGCCCTG | 90 | 7 | 17 | 6 | 0 | 1 |
| miR8572-x | 18 | GACCCCGTCGTTCGAATC | 89 | 11 | 4 | 5 | 7 | 3 |
| miR6300-y | 20 | GTCGTTGTAGTATAGTGGTA | 86 | 8 | 7 | 8 | 3 | 3 |
| miR169-y | 22 | CGGCAAGTTGTCATTGGCTACA | 84 | 4 | 15 | 9 | 0 | 0 |
| miR6167-y | 19 | TACCCGGGTGGAAGCTTCG | 84 | 7 | 12 | 7 | 1 | 1 |
| miR162-x | 21 | GGACGCAGCGGTTCATCGATC | 81 | 8 | 10 | 4 | 1 | 4 |
| miR845-x | 22 | CGGGCTCTGATACCAATTGTTG | 63 | 4 | 9 | 3 | 2 | 3 |
| miR395-x | 21 | TTCCCCGGAACACTTCATTGG | 60 | 3 | 12 | 5 | 0 | 0 |
| miR5368-y | 23 | AGGGACAGTCTCAGGTAGACAGC | 54 | 3 | 4 | 7 | 3 | 1 |
| miR159-x | 18 | AGCTCCTTGAAGTCCAAT | 53 | 2 | 3 | 3 | 1 | 9 |
| miR845-y | 22 | GAGGCTCTGATACCACTTGTTG | 51 | 5 | 8 | 2 | 2 | 0 |
| miR5054-y | 19 | TTTCCCACGGACGGCGCCA | 34 | 2 | 3 | 4 | 0 | 2 |
| miR5055-x | 21 | TCTCGCAACTGAGCTCGGCGT | 32 | 4 | 2 | 0 | 2 | 2 |
| miR477-x | 21 | ACTCTCCCTCAAGGACTTCTG | 30 | 1 | 4 | 2 | 1 | 3 |
| miR9773-y | 21 | TCTGTTTTTATGTTATTTCGT | 30 | 4 | 3 | 2 | 1 | 0 |
| miR482-z | 24 | TTCTTCCCAATCCCTCCCATTCCA | 29 | 1 | 5 | 1 | 0 | 2 |
| miR393-y | 21 | ATCATGCTATCCCTTTGGATT | 26 | 1 | 2 | 3 | 0 | 2 |
| miR5485-x | 24 | GTGACAAATGGTATCAGAGCAATG | 24 | 2 | 4 | 2 | 0 | 0 |
| miR6171-y | 21 | TGTGGATGGCTGAAGGCATTG | 23 | 0 | 2 | 3 | 0 | 3 |
| miR1122-y | 22 | ACTAATATTATGGGACGGAGGG | 20 | 1 | 5 | 1 | 0 | 1 |
| miR1871-y | 20 | AATGGCTCTGATACCATGTT | 19 | 2 | 2 | 1 | 1 | 1 |
| miR9726-y | 20 | GTAGGCATTATTTTTTTTTT | 19 | 1 | 3 | 0 | 0 | 2 |
| miR2938-y | 18 | TTGATCTTCTGAGAAGGT | 18 | 2 | 1 | 1 | 1 | 1 |
| miR172-x | 21 | GCGGCATCATTAAGATTCACA | 17 | 1 | 3 | 1 | 1 | 0 |
| miR1133-x | 23 | ATATACTCCCTCCGTCCCAAAAT | 14 | 2 | 0 | 1 | 0 | 2 |
| miR1919-x | 19 | TGTCGCAGATGATTTTGGC | 14 | 1 | 3 | 1 | 0 | 0 |
| miR4403-x | 24 | ACACGGACACGGGACACGACACGG | 14 | 2 | 2 | 0 | 0 | 1 |
| miR7782-y | 20 | AAACCTGCTCTGATACCATG | 14 | 0 | 1 | 1 | 2 | 1 |
| miR1515-x | 22 | TCATTTTTGCGTGCAGTGATCC | 13 | 0 | 2 | 3 | 0 | 0 |
| miR7122-y | 20 | CCGTGTTTCTCTGTCTAAAG | 13 | 1 | 2 | 1 | 0 | 1 |
| miR1128-x | 24 | ATACTACTCCCTCCGTCCCAAAAT | 12 | 4 | 0 | 0 | 0 | 0 |
| miR2111-x | 21 | TAATCTGCATCCTGAGGTTTG | 11 | 1 | 1 | 1 | 0 | 1 |
| miR5049-y | 24 | AGACCATTATTTTGGGACGGAGGG | 11 | 0 | 3 | 0 | 0 | 1 |
| miR393-x | 21 | TCCAAAGGGATCGCATTGATC | 9 | 0 | 2 | 1 | 0 | 0 |
| miR157-x | 21 | TTGACAGAAGATAGAGAGCAC | 8 | 0 | 1 | 1 | 0 | 1 |
| miR4995-x | 18 | ATAGGCAGTGGCTTGGTC | 8 | 1 | 0 | 1 | 0 | 1 |
| miR3630-x | 19 | TGCCAGTGAGGATATCAGA | 6 | 0 | 0 | 0 | 2 | 0 |
| miR474-y | 20 | ATGCTGTTGGCTTTGGCTGG | 6 | 0 | 0 | 0 | 0 | 2 |
| miR4994-y | 18 | ATTCTAGAGCTAATACAC | 6 | 0 | 1 | 1 | 1 | 0 |
| miR5658-x | 18 | ATGATGATGATGATGATG | 5 | 1 | 0 | 1 | 0 | 0 |
| miR1171-x | 24 | AGTGGAGTGTAGTGGAGCGGAGTG | 4 | 1 | 0 | 1 | 0 | 0 |
| miR156-y | 20 | ATTGGAGTGAAGGGAGCTCC | 4 | 0 | 0 | 1 | 0 | 1 |
| miR3438-x | 18 | TCAAAGATTAAGCCATGA | 4 | 1 | 0 | 0 | 0 | 1 |
| miR390-y | 21 | CGCTGTCCATCCTGAGTTTCA | 4 | 0 | 0 | 1 | 0 | 1 |
| miR403-x | 21 | AGTTTGTGCGTGAATTTAACA | 4 | 0 | 1 | 0 | 1 | 0 |
| miR8007-y | 18 | CGAAAGATGAAAAGGACC | 4 | 0 | 0 | 0 | 1 | 0 |
| miR858-y | 22 | CTTCGTTGTCTGTTCGACCTTG | 4 | 0 | 0 | 1 | 0 | 0 |
| miR1045-x | 18 | TTGGCTTTTTGACTTTTT | 3 | 0 | 1 | 0 | 0 | 0 |
| miR2275-x | 19 | AAGTGGAGGAAAAGAAACT | 3 | 0 | 0 | 0 | 1 | 0 |
| miR3512-y | 18 | TAGCCAATGATGACAAAT | 3 | 0 | 0 | 0 | 1 | 0 |
| miR5021-x | 18 | ATTGAGAGGAAGGAGAAG | 3 | 0 | 0 | 0 | 1 | 0 |
| miR1042-x | 18 | TGTGCAGGTAGTAGATAG | 2 | 0 | 0 | 1 | 0 | 0 |
| miR1057-y | 18 | TATTCTGGTGTCCTAGCT | 2 | 0 | 0 | 1 | 0 | 0 |
| miR1063-x | 21 | TACATCTTGGTGTAGTGCATC | 2 | 0 | 1 | 0 | 0 | 0 |
| miR1127-x | 18 | AAGTACTCCCTCCGTCCT | 2 | 0 | 0 | 0 | 0 | 1 |
| miR164-y | 18 | TGTGCCCTTCCTCTTCAC | 2 | 0 | 0 | 1 | 0 | 0 |
| miR1865-x | 18 | TAGTGGTGATGATTCTTC | 2 | 0 | 0 | 0 | 1 | 0 |
| miR1866-y | 19 | CTAAAATTCCTGAAAAATT | 2 | 0 | 0 | 1 | 0 | 0 |
| miR2083-y | 18 | GTTGAGGAGTGCAAGAAG | 2 | 0 | 0 | 1 | 0 | 0 |
| miR2111-y | 21 | GTCCTCGGGTTGCAGATTACT | 2 | 0 | 1 | 0 | 0 | 0 |
| miR2275-y | 19 | GTCAGTTTCTTCTAAGATC | 2 | 0 | 1 | 0 | 0 | 0 |
| miR4388-y | 18 | AGGGACCAAATTGACACA | 2 | 0 | 0 | 0 | 0 | 1 |
| miR474-x | 18 | TTGGTGAGTTTGGCTGGG | 2 | 0 | 0 | 0 | 1 | 0 |
| miR5061-y | 23 | TCTGTTCTGTTCTGTTCGGTACC | 2 | 0 | 1 | 0 | 0 | 0 |
| miR5181-y | 20 | TCTTTTGGATTGAAGGGAGT | 2 | 0 | 0 | 1 | 0 | 0 |
| miR5385-x | 19 | TCACCACCACCACCACCGC | 2 | 0 | 0 | 0 | 0 | 1 |
| miR5523-y | 21 | AGGAGGAACGTATTTACTAGT | 2 | 0 | 0 | 1 | 0 | 0 |
| miR5538-x | 24 | ACTGAACTCAATCACTTGCTGCCG | 2 | 0 | 0 | 1 | 0 | 0 |
| miR5653-y | 24 | ATGAGTTGAGTTGAGTTGGGTTGG | 2 | 0 | 1 | 0 | 0 | 0 |
| miR5834-x | 18 | GATGTAGACATTGGTGCT | 2 | 0 | 0 | 0 | 1 | 0 |
| miR6173-y | 21 | TAGCCGTAAACGATGGATACC | 2 | 0 | 1 | 0 | 0 | 0 |
| miR6281-x | 18 | TTTTAGAGAGAGAGAGAG | 2 | 0 | 1 | 0 | 0 | 0 |
| miR6485-x | 18 | CCTAGGATGTAGAAGATC | 2 | 0 | 0 | 0 | 1 | 0 |
| miR7528-y | 18 | AATGCTAATCTGAGGCTC | 2 | 0 | 1 | 0 | 0 | 0 |
| miR7711-x | 20 | AAAAGTGAAATTAACTCATT | 2 | 0 | 0 | 1 | 0 | 0 |
| miR7713-x | 18 | AGTAAATGATGGAACAGC | 2 | 0 | 0 | 0 | 1 | 0 |
| miR7717-x | 18 | TTTCTTGGGCCACTCAGA | 2 | 0 | 0 | 0 | 1 | 0 |
| miR7725-x | 18 | TATTCTCCACCTGATATT | 2 | 0 | 1 | 0 | 0 | 0 |
| miR7762-y | 18 | ATTTTGTCATCAACGGGC | 2 | 0 | 0 | 0 | 1 | 0 |
| miR781-y | 18 | ATCCAGAAAACTCAAACT | 2 | 1 | 0 | 0 | 0 | 0 |
| miR845-z | 22 | AGGCTCTGATACCAATTGTTGG | 2 | 0 | 0 | 1 | 0 | 0 |
| miR8590-y | 18 | TTTGTGGAAAAAAAAATT | 2 | 1 | 0 | 0 | 0 | 0 |
| miR8665-y | 18 | TTATTATTATATAGGTCA | 2 | 0 | 1 | 0 | 0 | 0 |
| miR8681-y | 18 | TGGCATCGTTGAGGGTCT | 2 | 0 | 0 | 0 | 1 | 0 |
| miR870-y | 18 | TTTGGTGTTTCTTTCATC | 2 | 1 | 0 | 0 | 0 | 0 |
| miR9569-x | 24 | ATGAGTTATCATTGGGATTGTGTC | 2 | 0 | 0 | 1 | 0 | 0 |
| miR9759-y | 18 | ATAAGAAAGTAGAATTTT | 2 | 1 | 0 | 0 | 0 | 0 |
| miR9863-x | 19 | TGGTTATGATCTGATTCTC | 2 | 0 | 0 | 0 | 1 | 0 |
