# Supplementary material for: Conserved MicroRNA Act Boldly During Sprout Development and Quality Formation in Pingyang Tezaocha (Camellia sinensis)
Source: Front Genet. 2019 Mar 28;10:237. doi: 10.3389/fgene.2019.00237 (PMC6455055; doi:10.3389/fgene.2019.00237)
Supplement: Supplementary Table 6 — 122 precursors and 99 kinds of characteristic hairpin structures, with their length and energy information. [file Table_6.DOCX]

Supplementary Table 6 122 precursors and 99 kinds of characteristic hairpin structures, with their length and energy information.

| mature_id | Hairpin | | | |
| --- | --- | --- | --- | --- |
|  | id | Length  (nt) | Energy  (kcal/mol) | structure |
| miR157-x | m0001 | 127 | -42 | (((((.((((.(((((((((((((((((((((((.((....(((((.........))))).....)).)))))))))))))).))))))))).))......................))..))))). |
| miR319-y | m0002 | 198 | -85.4 | .((((((((..(((.(((((((((((((((((..((((((.(((.((((((..(((((((..((.(((..((((((((.((..((((((((.....(((....))).....))))))))..)).))))))))..))).))...))))))))))))).)))))))))..))))))))))))))))).))).)))))))) |
| miR403-y | m0003 | 157 | -42.1 | .(((.(((((.(.((((((((((((((((((((((((.......))).............((.((..(((((..............((........))..............)))))..)).))))))))))))))))))))))).).))))).))) |
| miR390-x | m0004 | 97 | -45.8 | ((((.((((.((((((((((.(((((((((.(((((..((.(((((......))))))).))))).))))))))).)))))))))).)))).)))). |
| miR1863-y | m0005 | 75 | -32.6 | .(((..((((((((.(((((((((((((((((.........))))))))))))))))).))))))))....))). |
| miR1871-y | m0006 | 73 | -35.3 | (((..((((((((((.((((((((.((((((((......)))))))).)))))))).))))))))))..))). |
| miR167-x | m0007 | 134 | -57.5 | ((((((..(((((.(((((((((((((((((((...((((((((((.........(...(((....)))...)..........))))..))))))...))))))))))))..))))))).)))))..)))))). |
| miR169-x | m0008 | 274 | -78.1 | .((((((...((((((((((.((.((.(((((...(((((((((((.(((((((((((..((((((.(((((((((((((((...(((.((......)).)))...))))).))))))(((((((((((.(((((((..............)))))))..)))))))))))....)))).))))))..))))))).....((((.((((......))))))))...)))))))))..)))))).))))))).))))))))))))...)))))). |
| miR164-x | m0009 | 101 | -52.3 | .(((((.(((((((((((..((((((((((((((....((....(((((......)))))....))))).)))))))))))..))))))))))).))))). |
| miR160-x | m0010 | 100 | -47.8 | (((..(.((((((.(((((((.((((((((((((((.(((.(((((((....))))))).))).)))))))))))))).))))))).)))))))..))). |
| miR168-y | m0011 | 71 | -22.3 | .(((((..(((((((.(.(((((.((((((.((......)).).))))).)))))).))))))).).)))) |
| miR408-y | m0012 | 122 | -58.7 | .(((((((((.(((((((((.(((.(((.(((.((((.(((.....((((.(((.(((((........))))).))).))))))).)))).))).))).))).))))))))).))))))))) |
| miR395-y | m0013 | 102 | -42.7 | .(((..(((..(((((((((((.(((((((((.((((((..........................)))))).))))))))))))))))))))..)))..))) |
| miR157-x | m0014 | 106 | -47.8 | ((((((.((((((((((((((.((((((((((((((..(((((.(((......))).)))))..))))..))))))))))..))))))))))))))...)))))). |
| miR168-x | m0015 | 136 | -66.6 | ...(((((((((.((((((((((.((((((((((.(((((......((((((....))))))..........((((((((.....)))))))).))))).)))))))))).)))))))))).)))))))))..... |
| miR168-y | m0015 | 136 | -66.6 | ...(((((((((.((((((((((.((((((((((.(((((......((((((....))))))..........((((((((.....)))))))).))))).)))))))))).)))))))))).)))))))))..... |
| miR168-x | m0016 | 136 | -66.6 | ...(((((((((.((((((((((.((((((((((.(((((......((((((....))))))..........((((((((.....)))))))).))))).)))))))))).)))))))))).)))))))))..... |
| miR168-y | m0016 | 136 | -66.6 | ...(((((((((.((((((((((.((((((((((.(((((......((((((....))))))..........((((((((.....)))))))).))))).)))))))))).)))))))))).)))))))))..... |
| miR408-y | m0017 | 112 | -51.8 | ...(((((((((.((((((.(((.(((.(((.((((.(((((......(((((((.......)))))))..))))))))).))).))).))).)))))).)))))))))... |
| miR384-x | m0018 | 109 | -43.4 | ((((((((((((((((...(((((((((((....(((....(((....................))).....)))..)))))))))))...)))))).)))))))))). |
| miR164-x | m0019 | 86 | -47 | (((.((.(((((((((((..(((((((((((.((((((.((....)).)))))).)))))))))))..))))))))))).))))). |
| miR7972-y | m0020 | 288 | -76.9 | ..(((((((.(((((((((((((((((((((((((((....))).(((...........)))..........(((.(((((((......(((((((((((((.....)))))))))))))...........((((.((((.(((.(((........))).))).)))).))))....(((((....)))))((((....))))..................((.((((......)))))).))))))).)))..)))))))))))))))))))))))).))))))).. |
| miR171-z | m0021 | 107 | -40 | ......((((((.((((((((((.(((((((((.(((..(..(((((((((...)).)))))))..)..).)).))))))))).)))))))))).))))))...... |
| miR393-x | m0022 | 97 | -44.9 | (((((((((.(((.(((((((((((((.((((((((...((((........))))....)).)))))).))))))))))))).))).))))))))). |
| miR171-y | m0023 | 132 | -44.8 | ....(((((.(((((((((.(((((((.(((((....................((((((((..(((((((((....)))))))))...))))))))...))))).))))))).))))))))).))))).... |
| miR166-y | m0024 | 162 | -54.3 | ......(((.((((((((..((((((.((((.(((.((..(.(((((....(((.(((((((((.(((....))).))))))((((((....))))))..))).)))......)))))...)..)).))).)))).))))))..)))))))).)))...... |
| miR319-y | m0025 | 203 | -81 | ...((((((......(((.(((((((((((((((((..((((((.((((...(((...((((((.((.(((..((((((((....(((.(((.(((...((.((((.....)))).)).)))))).)))))))))))..))).)).))))))....)))))))..))))))..))))))))))))))))).)))))))))... |
| miR319-y | m0026 | 188 | -83 | .......(((((.(((((((((((((((((...((((((((((....(((((((((.(((.(((..((((((((.((..((((((((..............))))))))..)).))))))))..))).)).).)))))))))...))))))))))...))))))))))))))))).)))))....... |
| miR8558-y | m0027 | 110 | -55.3 | .(((((((((...((.(((((((((.(((.(((((((.((((((......))))))...((((....))))..))))))).))).)).))))))).))...))))))))) |
| miR472-y | m0027 | 110 | -55.3 | .(((((((((...((.(((((((((.(((.(((((((.((((((......))))))...((((....))))..))))))).))).)).))))))).))...))))))))) |
| miR156-x | m0028 | 111 | -54.4 | (((((((.((((((((((((((((((((((((((((..(((((((((((......)))))))))))..).)).)))))))))).)))))))))))))))....))))))). |
| miR166-y | m0029 | 147 | -55.7 | ......(((.((((((((..((((((.((((.((((.((((((..((.((((..((((((((((((.(.....).))))))))))...))..))))..))..))))))..).))).)))).))))))..)))))))).)))...... |
| miR396-x | m0030 | 133 | -62.6 | ....(((((((((((((.(((((((((((((((((((.((((((((((((((..(((..(........)..)))))))))).....))))))).))))))))))))))))))).)))))).....))))))). |
| miR396-y | m0030 | 133 | -62.6 | ....(((((((((((((.(((((((((((((((((((.((((((((((((((..(((..(........)..)))))))))).....))))))).))))))))))))))))))).)))))).....))))))). |
| miR482-x | m0031 | 92 | -43.7 | ((((((((.((((((((((.(.((((((((((.(((.(((((......))))).))).))).))))))).).)))))))))).)))))))). |
| miR2118-y | m0032 | 226 | -71.3 | (((((((((((((((((....((((((.((.((((((((.((((((.(((((.(((((((((.((.((((.((((((((((....((((.((((......)))).)))).........((......))..............)))))))))))))))).)))))))))))))).))...)))).)))))))).)).)))).))..))))).))))..)))))))). |
| miR2118-x | m0032 | 226 | -71.3 | (((((((((((((((((....((((((.((.((((((((.((((((.(((((.(((((((((.((.((((.((((((((((....((((.((((......)))).)))).........((......))..............)))))))))))))))).)))))))))))))).))...)))).)))))))).)).)))).))..))))).))))..)))))))). |
| miR403-y | m0033 | 145 | -45.8 | .((((..(((.(.((((((((((.(((((.(((.((.(((.....((((.......((((((.....(((.......)))))))))...........)))).....))).)).))).))))).)))))))))).).)))..)))) |
| miR159-x | m0034 | 205 | -83 | .(((((.(((((((((((((((..(((((((.(.((((((..((.(((..(((..((((..((((.((((.(..((.((...(((((((((....((............))..)))))))))...)).))..).)))).))))..))))..)))..)))...))..)))))).).)))))))..)))))))))))).)))))))) |
| miR159-y | m0034 | 205 | -83 | .(((((.(((((((((((((((..(((((((.(.((((((..((.(((..(((..((((..((((.((((.(..((.((...(((((((((....((............))..)))))))))...)).))..).)))).))))..))))..)))..)))...))..)))))).).)))))))..)))))))))))).)))))))) |
| miR172-y | m0035 | 109 | -50.4 | .(((.((((.(((((((((((((((((((((.(((.(((((.....(((((.......)))))...)))))....))).))))))))))))))))))))).)))).))) |
| miR170-x | m0036 | 142 | -43.8 | ..((((((.((.((((..(((((((((.(((((((.(((((..(((((...((((((...........(((((.......))))))))))).)))))..))))).))))))).)))))))))..))))))))))))...... |
| miR171-y | m0036 | 142 | -43.8 | ..((((((.((.((((..(((((((((.(((((((.(((((..(((((...((((((...........(((((.......))))))))))).)))))..))))).))))))).)))))))))..))))))))))))...... |
| miR395-x | m0037 | 172 | -63.9 | ..(((((.((.(..((((((((((((.((((((((((................((((((((....))))))))((.(((((((....)))).))).))((((..(((.............)))..))))...)))))))))).))))))))))))..).)).)))))..... |
| miR395-y | m0037 | 172 | -63.9 | ..(((((.((.(..((((((((((((.((((((((((................((((((((....))))))))((.(((((((....)))).))).))((((..(((.............)))..))))...)))))))))).))))))))))))..).)).)))))..... |
| miR395-y | m0038 | 104 | -48 | .(((..(((.((((.((((((.(((((((((.(((((......(((.((((....)))).))).....))))).))))))))).)))))).)))).)))..))) |
| miR395-x | m0039 | 171 | -66.2 | ..(((((.((.(..((((((((((((.((((((((((...((.((((((((((((((((((....)))))))).)))).))))))))...........((((..(((............)))..))))...)))))))))).))))))))))))..).)).)))))..... |
| miR395-y | m0039 | 171 | -66.2 | ..(((((.((.(..((((((((((((.((((((((((...((.((((((((((((((((((....)))))))).)))).))))))))...........((((..(((............)))..))))...)))))))))).))))))))))))..).)).)))))..... |
| miR395-y | m0040 | 109 | -47.3 | ..((((.(((..(((((((((((..(((((((((.(((((((....)))))))...(((((....)))))........)))))))))..)))))))))))))))))).. |
| miR156-x | m0041 | 103 | -53.4 | ...((..(((.((((((((((((((((((((.(((((((....(((((((....)))))))..))))))).)))))))).)))))))))))).)))..))... |
| miR399-y | m0042 | 126 | -51.8 | ....((((((((((((.(((((((.(((((((((((((..(((((..((.(((((.((.....)).))))).))....)))))....))).)))))))))).))))))).)))))))))))).... |
| miR6300-y | m0043 | 288 | -62 | ..((((.((((((.((.((.(((((((.(((.(((((((((.......)))))....(((((.......)))))....(((((....))))).......(((((((.((((((......))))))((((..(((..((((.....)))).....)))..))))..............(((((....))))).)))))))(((((...(((((((....)))))))....)))))((((........))))..))))))).)))))))..)).)).)))))).)))).. |
| miR171-z | m0044 | 111 | -42.8 | ......((((((.((((((((((.(((((((((.((.((((..((((((((......)).).)))))...)).)))).))))))))).)))))))))).))))))...... |
| miR166-y | m0045 | 152 | -49.3 | ..((((((....)))(((.((.(((((..((((((.((((((.(((...(((.(((((((((......(((((................))))).......))))))))).))).))).)))))).))))))..))))).)).))).))).. |
| miR395-x | m0046 | 170 | -70.4 | ..(((((.((((..((((((((((((.((((((((((.((((((((((.(((((((((.((....)).))))).)))).)))))........(((.((((.(((........))))))).))).))))).)))))))))).))))))))))))..)))).)))))..... |
| miR395-y | m0046 | 170 | -70.4 | ..(((((.((((..((((((((((((.((((((((((.((((((((((.(((((((((.((....)).))))).)))).)))))........(((.((((.(((........))))))).))).))))).)))))))))).))))))))))))..)))).)))))..... |
| miR157-x | m0047 | 109 | -52.4 | (((((.((((.(((((((((((((((((((((((.(((((.(((((((...))))))).))).)))))))))))))).))))))))))).......))))...))))). |
| miR157-x | m0048 | 109 | -52.4 | (((((.((((.(((((((((((((((((((((((.(((((.(((((((...))))))).))).)))))))))))))).))))))))))).......))))...))))). |
| miR164-x | m0049 | 176 | -55.9 | .((((((.(((((.((((..(((((((((...(((((((((((.((...(((........((((.((((.....(((...((((((......)))).)).)))....))).).)))).....)))..)))))))))))))...)))))))))..)))).))))).))....)))). |
| miR396-x | m0050 | 118 | -42.5 | ((((((..((((((((((.((((((((((((((...((((((.(((............................))).)))))))))))))))))))).))))))))))..)))))). |
| miR171-y | m0051 | 96 | -46.2 | ......(((.(((((((((((((((((((.((((((.((.((((((....)))))).)).)))))).))))))))))))))))))).)))...... |
| miR390-x | m0052 | 135 | -65.4 | ((((((((((((((((((((.((((((((((((((....((((.((((..((((......((((......))))......))))..))))))))....))).))))))))))).)))))))))))))))))))). |
| miR168-x | m0053 | 135 | -55.3 | ........((((.((((.(((((.((((((((((.(((((.((...(((((...)))))((((((.((((((.....))))))..))))))))))))).)))))))))).))))).)))).)))).......... |
| miR168-y | m0053 | 135 | -55.3 | ........((((.((((.(((((.((((((((((.(((((.((...(((((...)))))((((((.((((((.....))))))..))))))))))))).)))))))))).))))).)))).)))).......... |
| miR395-y | m0054 | 288 | -84.9 | .(((((.(((.((((((((((((((((((((((.((..(((((....((((((((............((((((((.((.(((((((........))))....((((((((..(((((.(((.((((...))))...))).)))))))))).)))......))).)).))).)))))(((...)))......))))))))..(((((..(..((((........))))..)..))))).........)))))..)).)))))))))))))))))))))).)))))))). |
| miR395-y | m0055 | 288 | -84.9 | .(((((.(((.((((((((((((((((((((((.((..(((((....((((((((............((((((((.((.(((((((........))))....((((((((..(((((.(((.((((...))))...))).)))))))))).)))......))).)).))).)))))(((...)))......))))))))..(((((..(..((((........))))..)..))))).........)))))..)).)))))))))))))))))))))).)))))))). |
| miR395-y | m0056 | 211 | -63.5 | .......(((((((((((((((((((((((((....((((..(((((....)))))..))))................((((((((............))))))))...((((...((.(((.(.(((........))).)..))).))...))))((.((((((...)))))).)))))))))))))))))))))))..))))....... |
| miR395-y | m0057 | 105 | -52.8 | .((.(..((((((((((((.((((((((((.(((((((....((((((((....)))).))))....))))))).)))))))))).))))))))))))..).)). |
| miR393-x | m0058 | 157 | -50.2 | ....((...(((((((((.((((((((((((.((((.(((((.((.(((((..................(((.........)))...((((((....)))))).))).)).)).))))))))).)))))))))))).)))))))))...))...... |
| miR393-y | m0058 | 157 | -50.2 | ....((...(((((((((.((((((((((((.((((.(((((.((.(((((..................(((.........)))...((((((....)))))).))).)).)).))))))))).)))))))))))).)))))))))...))...... |
| miR168-x | m0059 | 143 | -74.6 | ...((((.(((((((((.(((((.((((((((((.(((((.(((.((((((((((.(...(((.((...........)).))).).)))))))))).))).))))).)))))))))).))))).))))))))).))))..... |
| miR168-y | m0059 | 143 | -74.6 | ...((((.(((((((((.(((((.((((((((((.(((((.(((.((((((((((.(...(((.((...........)).))).).)))))))))).))).))))).)))))))))).))))).))))))))).))))..... |
| miR172-x | m0060 | 131 | -67.4 | ...((((((((.((((((((((((((((((((((((((.(((.(((((..(((((((((((...)))))))))))....)))))...))).)))))))))))))))))))))))))).))))))))..... |
| miR172-y | m0060 | 131 | -67.4 | ...((((((((.((((((((((((((((((((((((((.(((.(((((..(((((((((((...)))))))))))....)))))...))).)))))))))))))))))))))))))).))))))))..... |
| miR160-x | m0061 | 122 | -45 | .................((((((.(((((((.(((((((((((((..(((.(((.((......)).))).)))..))))))))))))).))))))).))))))..(((......)))..... |
| miR160-y | m0061 | 122 | -45 | .................((((((.(((((((.(((((((((((((..(((.(((.((......)).))).)))..))))))))))))).))))))).))))))..(((......)))..... |
| miR384-x | m0062 | 95 | -48.3 | ((((((.((((((((((((((.((((((((((((((....(((....)))....)))))....))))))))).)))))))))))))).)))))). |
| miR166-x | m0063 | 110 | -50.5 | .(((((((((((((((..(.((((.(((((((((((((..(((((.(........).))))).........))))))))))))).)))).)..)))))))))).))))). |
| miR166-y | m0064 | 82 | -47.3 | ...((((((.((((((((..((((((((((.((((.(((....))))).)).))))))))))..)))))))).))))))... |
| miR395-x | m0065 | 171 | -60.4 | ..(((((.((.(..((.(((((((((.((((((((((.((((((((((((..(((((((.(....).)))))))....))))))).(((((((.......................)))).))).))))).)))))))))).))))))))).))..).)).)))))..... |
| miR395-y | m0065 | 171 | -60.4 | ..(((((.((.(..((.(((((((((.((((((((((.((((((((((((..(((((((.(....).)))))))....))))))).(((((((.......................)))).))).))))).)))))))))).))))))))).))..).)).)))))..... |
| miR535-x | m0066 | 112 | -59.2 | ..........((((((((((((((((((((((.((..(((.((((((.....)))))).)))..)).))))))))))))))))))))))...((((((((....)))))))) |
| miR395-x | m0067 | 148 | -61.3 | ....(((((((((((((((((((((((.(((((((((((((((((((((((....)))))))).)))).)))))).(((((((......((((....)))).....)))).))).))))).))))))))))))))))))))))).... |
| miR166-y | m0068 | 108 | -59.4 | .....(((((((((((((..((((((((((((((((((((((..(((.((((...)))))))..))..))))))))))))))))))))..)))))))))))))..... |
| miR384-x | m0069 | 106 | -37.05 | (((((.((((((((((...(((((((((((.((......................................)).)))))))))))...)))))).)))).))))). |
| miR396-x | m0070 | 108 | -54 | (((((((((((((((((((((((((((((((((...........((((..........))))............))))))))))))))))))))))))))))))))). |
| miR166-y | m0071 | 102 | -48.7 | ..((.(((((.((((((((..(((((((((((((.(((.((..(((.((....))..)))..)).))).)))))))))))))..)))))))).))))))).. |
| miR159-x | m0072 | 205 | -83 | .(((((.(((((((((((((((..(((((((.(.((((((..((.(((..(((..((((..((((.((((.(..((.((...(((((((((....((............))..)))))))))...)).))..).)))).))))..))))..)))..)))...))..)))))).).)))))))..)))))))))))).)))))))) |
| miR159-y | m0072 | 205 | -83 | .(((((.(((((((((((((((..(((((((.(.((((((..((.(((..(((..((((..((((.((((.(..((.((...(((((((((....((............))..)))))))))...)).))..).)))).))))..))))..)))..)))...))..)))))).).)))))))..)))))))))))).)))))))) |
| miR4414-x | m0073 | 110 | -45 | ...(((.(((.(((.(((..(((((((.(((..(((((((......(((((.(((((...)))))))))))))))))..))).)))))))..))).))).))).)))... |
| miR168-x | m0074 | 114 | -45.1 | ((.((((.(((((.((((((((((.(((((.((..((((((.....((((((((......)))))..)))..))))))..)).))))).)))))))))).))))).)))).)). |
| miR169-x | m0075 | 271 | -77.4 | .((((((.(.((((((((((.((.((((((((.(((.....((((((((.((((((.......)))))).))).............(((.(((((((.((((.(.(((((.((((.(((((((((((((..((((((..............))))))..))))))))))))).)))).....))))).).))))))))))).))).....((((........))))))))).....)))..)))))))))))))))))))).).)))))). |
| miR171-y | m0076 | 96 | -46.7 | .((.(((..(((((((((.(((((((((((((((.(((...(((((....)))))...))).))))))))))))))).)))))))))..))).)). |
| miR1871-y | m0077 | 73 | -35.3 | (((..((((((((((.((((((((.((((((((......)))))))).)))))))).))))))))))..))). |
| miR2111-x | m0078 | 87 | -48.5 | (((((..((((((((((((.(((((((.(((((((((((.......))))).)))))).))))))).))))))))))))..))))). |
| miR162-x | m0079 | 131 | -47 | .......(((.(.((((.(((((.((((.((((.(((((((.(((((.((..(((..(((..........)))..)))..))))))).))))))).)))).)))).))))).)...)))).)))....... |
| miR162-y | m0079 | 131 | -47 | .......(((.(.((((.(((((.((((.((((.(((((((.(((((.((..(((..(((..........)))..)))..))))))).))))))).)))).)))).))))).)...)))).)))....... |
| miR156-x | m0080 | 114 | -56.3 | (((((((...((((((((((((((((((((((((((..(((((.((((((....)))))).)))))...))).)))))))))).)))))))))))))....)).....))))). |
| miR319-y | m0081 | 202 | -90.2 | ..((..((((((((..(((((((((((((((((((..((.((((((.....((((((..((.(((((.(((..(((.(((.((((((((..((((..((.............)).))))..)))))))).))).)))..))).))))).))))))))...)))))).))..))))))))))))))))))))))))))))).. |
| miR7122-x | m0082 | 122 | -45.2 | .(((((((.((((.(((((((((.(((((((((((....((..((..((..(((...((.......))...)))..))..))..))..)))))))))))))))))))).)))).))))))). |
| miR171-y | m0083 | 107 | -42.7 | .((((((.........(((.(((((((((.(((((((((..((((((((((..((((....))))))))).)))))..))))))))).))))))))).))))))))) |
| miR162-x | m0084 | 130 | -47.6 | ..((((.(((...((((.(((((.((((.((((.(((((((.(((((.((..(((...((.........))...)))..))))))).))))))).)))).)))).))))).)...)))..)))))))... |
| miR162-y | m0084 | 130 | -47.6 | ..((((.(((...((((.(((((.((((.((((.(((((((.(((((.((..(((...((.........))...)))..))))))).))))))).)))).)))).))))).)...)))..)))))))... |
| miR162-x | m0085 | 131 | -47 | .......(((.(.((((.(((((.((((.((((.(((((((.(((((.((..(((..(((..........)))..)))..))))))).))))))).)))).)))).))))).)...)))).)))....... |
| miR162-y | m0085 | 131 | -47 | .......(((.(.((((.(((((.((((.((((.(((((((.(((((.((..(((..(((..........)))..)))..))))))).))))))).)))).)))).))))).)...)))).)))....... |
| miR159-x | m0086 | 193 | -87.5 | .....(((.((((((((((((..(((((((...(((((.((.((((((..(((..((((.(((((.((((((..((.(((.(((((((((.((......))...))))))))).))).))..)))))).))))).))))..))).))).))).)).)))))..)))))))..)))))))))))).)))..... |
| miR159-y | m0086 | 193 | -87.5 | .....(((.((((((((((((..(((((((...(((((.((.((((((..(((..((((.(((((.((((((..((.(((.(((((((((.((......))...))))))))).))).))..)))))).))))).))))..))).))).))).)).)))))..)))))))..)))))))))))).)))..... |
| miR166-z | m0087 | 204 | -66.5 | .((...((((((((.((((((..((((((.(((((((((.(((((...))))).))))..(((((.(((.(((((.(.(((((((((((.......((((((((((.(((((((((....))))))))).)))))))))))))).)))))))).))))).))).)))))))))).))))))..)))))).))))))))...)). |
| miR166-y | m0088 | 133 | -51.4 | ....(((((.(.((((((..((((((.((((((((((..(((((((((((..((.((((.....)))))).))))))))...........)))..))))).))))).))))))..)))))).).))))).... |
| miR530-x | m0089 | 125 | -53.3 | (((((((.(((((((((((.(.((((((((.(((..((((..((((((((..((((.(((....))).))))..))))))))...)))).))).)))))))).).))))))))))).))))))). |
| miR319-y | m0090 | 158 | -53.7 | ..(((.(((((.(((((((((((((((((...(((((((((((..((((...)))).))))).))))))...........((((....)))).((((((((....((((...))))..))))))))..))))))))))))))))).)))))..))).. |
| miR319-x | m0091 | 219 | -86.9 | .((((.(.((......(((.(((((((((((.(((((..((((((.((((...(((((..((((((.((.(((..((((((((.(((((((..(((((.((((..............)))).)))))))))))).))))))))..))).)).))))))..)))))))))..))))))..))))).))))))))))).)))..))).))))......... |
| miR319-y | m0091 | 219 | -86.9 | .((((.(.((......(((.(((((((((((.(((((..((((((.((((...(((((..((((((.((.(((..((((((((.(((((((..(((((.((((..............)))).)))))))))))).))))))))..))).)).))))))..)))))))))..))))))..))))).))))))))))).)))..))).))))......... |
| miR390-x | m0092 | 155 | -57.2 | (((((((((.((((((((((.(((((((((((((((((((.(((((.(....(((..(((.((...............)).)))..))).....).))))).))........)))))....)))))))))))).)))))))))).))))))))). |
| miR157-x | m0093 | 104 | -42.7 | .....((.((.(((((((((((((((((((((.(.(((((.(((((.((....)).))))).))).)).).)))))))))))).))))))))).)).))..... |
| miR160-x | m0094 | 100 | -54.5 | .....(((.((((.(((((((.((((((((((.((..((((((((((........)))))))))).)).)))))))))).))))))).)))))))..... |
| miR171-y | m0095 | 217 | -83.6 | ....(((((((.(((.((((.((((((((((..(((.(((.(((((........))))).))).)))..)))))))))).)))).))).)))))...(((((..((((((((((........)))))))))))))))(((((((......)))))))((((((....(((((((((.((.......)).)))))))))..)))))).....)).... |
| miR167-x | m0096 | 142 | -59.84 | ...((((.((((((((..(((((.(((((.(((((((((((((...(((((((................................)))))))...)))))))))))))..).)))).)))))..))))))..)).))))... |
| miR167-y | m0096 | 142 | -59.84 | ...((((.((((((((..(((((.(((((.(((((((((((((...(((((((................................)))))))...)))))))))))))..).)))).)))))..))))))..)).))))... |
| miR396-x | m0097 | 130 | -46.5 | (((.((.(((((((((((((((((((((((((..(((((...(((.(((((........)))))))).)))))...(((((...)).))).......))))))))))))))))))))))))).)).))). |
| miR384-x | m0098 | 115 | -42.4 | ((.(((...((((((((((((.((.((((((.(((((((................)))))))...((((.......))))...)))))).)).))))))))))))...))).)). |
| miR166-y | m0099 | 132 | -45.9 | ...((..((((((((((((..((((((.(((((........((.(((((((((...........................))))))))).))........))))).))))))..)))))))))))).))... |
